# Computational Study on the Proton Reduction Potential of Co, Rh, and Ir Molecular Electrocatalysts for the Hydrogen Evolution Reaction

**DOI:** 10.1021/acsomega.4c03260

**Published:** 2024-11-26

**Authors:** Murugesan Panneerselvam, Madhavan Jaccob, Luciano T. Costa

**Affiliations:** † MolMod-CS—Instituto de Química, Universidade Federal Fluminense, Campos de Valonginho s/n, Centro, Niterói, Rio de Janeiro 24020-14, Brazil; ‡ Programa de Engenharia Química (PEQ/COPPE), Universidade Federal do Rio de Janeiro (UFRJ), Moniz Aragão, Rio de Janeiro 21941-594, Brazil; § Department of Chemistry and Computational Chemistry Laboratory, Loyola Institute of Frontier Energy (LIFE), Loyola College, Chennai, Tamil Nadu 600 034, India

## Abstract

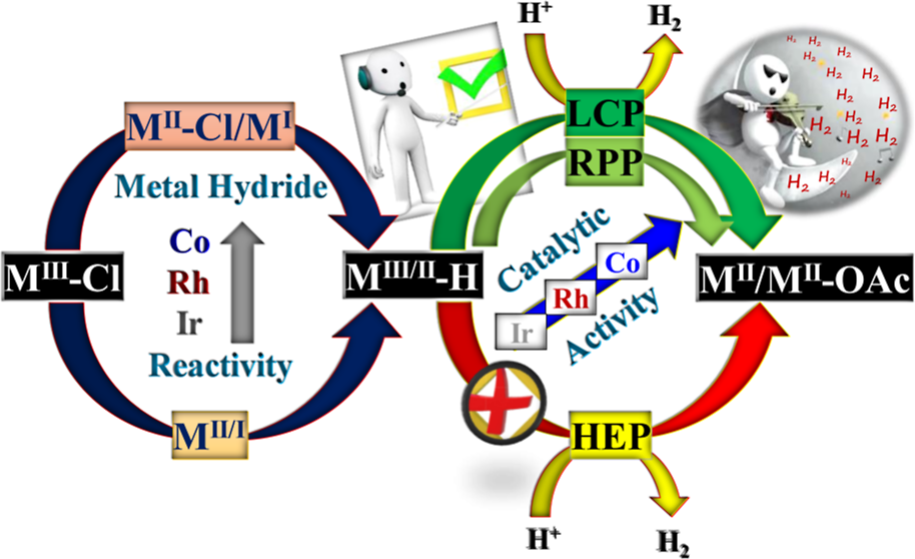

In this study, comprehensive density functional theory calculations were conducted to investigate the molecular mechanism of electrocatalytic proton reduction using group 9 transition metal bpaqH (2-(bis(pyridin-2-ylmethyl)amino)-*N*-(quinolin-8-yl)acetamide) complexes. The goal was to explore how variations in the structural and electronic properties among the three metal centers might impact the catalytic activity. All three metal complexes were observed to share a similar mechanism, primarily characterized by three key steps: heterolytic cleavage of H_2_ (HEP), reduction protonation (RPP), and ligand-centered protonation (LCP). Among these steps, the heterolytic cleavage of H_2_ (HEP) displayed the highest activation barrier for cobalt, rhodium, and iridium catalysts compared to those of the RPP and LCP pathways. In the RPP pathway, hydrogen evolution occurred from the M^II^–H intermediate using acetic acid as a proton donor at the open site. Conversely, in the LCP pathway, H–H bond formation took place between the hydride and the protonated bpaqH ligand, while the open site acted as the spectator. The enhanced activity of the cobalt complex stemmed from its robust σ-bond donation and higher hydride donor ability within the metal hydride species. Additionally, the cobalt complex demonstrated a necessary negative potential in the first (M^III/II^) and second (M^II/I^) reduction steps in both pathways. Notably, M^III/II^–H exhibited a more crucial negative potential for the cobalt complex compared to those of the other two metal complexes. Through an examination of kinetics and thermodynamics in the RPP and LCP processes, it was established that cobalt and rhodium catalysts outperformed the iridium ligand scaffold in producing molecular hydrogen after substituting cobalt metal with rhodium and iridium centers. These findings distinctly highlight the lower-energy activation barrier associated with LCP compared to alternative pathways. Moreover, they offer insights into the potential energy landscape governing hydrogen evolution reactions involving group 9 transition metal-based molecular electrocatalysts.

## 1 Introduction

Arguably, one of the most significant challenges we face in the 21st century revolves around mitigating the impact of energy consumption and production. The role of energy production in climate change varies based on the method used, with fossil fuel-based approaches being the largest contributors to human-driven climate change.^1−5^ These urgent global concerns stem from intense anthropogenic activities resulting from global economic growth, lifestyle changes, population surges, and technological advancements.^6^ Moreover, the escalating energy demands and the scarcity of conventional fossil fuels (oil, natural gas, and coal) have spurred advancements in sustainable clean energy technologies like solar, wind, and biofuels.^7^ Despite these advancements, fossil fuels continue to be extensively utilized for energy production and are anticipated to remain the predominant energy source until at least 2050.^8,9^ In this context, beyond its role as a carbon-free renewable fuel, hydrogen stands out as an alternative energy carrier applicable to the chemical industry. Notably, hydrogen production through the electrocatalytic hydrogen evolution reaction (HER; 2H^+^ + 2e^–^ = H_2_) has played a crucial role in the energy economy, and the future hinges on the development of sustainable and clean energy technologies.^10−12^ However, achieving efficient and long-term stable systems for water-splitting using solar light poses a significant challenge.^13−15^ Transition metal-based complexes for HER catalysts exhibit promising prospects for large-scale applications. Consequently, recent research endeavors have centered on developing earth–abundant transition metal catalysts for hydrogen production.^16−21^ In this vein, efficient mononuclear cobalt-based electrocatalysts with pentadentate or tetradentate polypyridyl ligand systems have been developed, showcasing long-term stability for homogeneous HER electrocatalysis in aqueous solutions.^12,22−27^ Additionally, numerous studies have delved into transition metal-based hydrogen evolution catalysts (HECs) for water reduction, employing elements from the platinum group, cobalt,^28−30^ iron,^31^ nickel,^32^ and molybdenum^33^ as central metal ions. Notably, cobalt and nickel stand out as the most commonly used metals for electrocatalytic water and proton reduction.^13,34^ Various strategies have been reported for synthesizing transition metal-based pyridine-containing macrocyclic complexes, demonstrating their potential across diverse catalytic applications, including stereoselective C–C and C–O bond-forming reactions and oxidation and reduction reactions.^35−38^ Furthermore, a diverse array of ligand skeletons paired with earth–abundant transition metal-based catalysts have shown promise in producing hydrogen from water, albeit at higher overpotentials, and demonstrating efficiency under both acidic and alkaline conditions.^39,40^ Conversely, ligand modification in metal catalysts has played a significant role in reducing overpotentials. Earlier reports on HECs have explored various ligand architectures, such as glyoxime complexes,^13,41−43^ N4 macrocyclic complexes,^44^ dithiolene complexes,^45,46^ cobalt complexes of base-containing diphosphines,^47,48^ and other polypyridine complexes.^49,50^

Even subtle changes in the catalyst structure and reaction conditions exert a significant influence on the reaction kinetics and process efficiency. Nippe et al.^27^ highlighted the pentadentate, redox-active ligand bpy2PYMe’s capacity to effectively stabilize low-valent metal species, thereby exhibiting noteworthy electrocatalytic proton reduction activity. Similarly, Karumban et al.^51^ successfully synthesized and characterized monoanionic amido pentadentate ligands, specifically ([Co(bpaqH)Cl]Cl) and [Co(bpaqH)(OH_2_)](ClO_4_)_2_), which displayed enhanced catalytic stability and demonstrated hydrogen production at substantially lower overpotentials (0.412 and 0.394 V) in aqueous solutions.^51^ Additionally, minimal shifts and insignificant spectral changes were observed at controlled potentials during electrocatalysis. As we theoretically reported in 2018, the involvement of reduced M(I) species in the catalytic cycle becomes apparent close to the M^II^/M^I^ redox couple.^30^ Drawing from available experimental^26^ and theoretical reports,^30−38,52,53^ protonation of low-valent M(I) species leads to the transient formation of M^III/II^-hydride species, a pivotal intermediate in molecular hydrogen evolution.^28−30^ The pathway for obtaining molecular hydrogen from cobalt-hydride species is primarily governed by two steps: (i) heterolytic pathway (HEP) for heterolytic cleavage of M(III)-hydride and (ii) reduction protonation pathway (RPP) by reducing M(III)-hydride to M(II)-hydride, followed by hydride cleavage from M–H to form H_2_, utilizing acetic acid as the proton source. This process significantly influences the stability, redox potential, acidity/basicity, and oxidation state of the metal center. Gray et al. established the necessity of additional redox properties for Co-triphos and glyoxime complexes through experimental electrochemical and theoretical studies, emphasizing the role of ligand design in molecular electrocatalysts.^54,55^ Notably, few reports have delved into the structural and spectral features of metal-hydride complexes. Rahman et al.^56^ crystallized hydridotetraamine cobalt(III) complexes, exploring their absorption spectral features. Similarly, Karumban and co-workers^51^ investigated the structural, redox, and electrocatalytic proton reduction properties of the bpaqH (2-(bis(pyridin-2-ylmethyl)amino)-*N*-(quinolin-8-yl)acetamide) complex. Despite extensive research on homogeneous systems for hydrogen production, there are relatively few reported instances involving rhodium and iridium catalysts.^17,18,21,57,58^

**Scheme 1:**
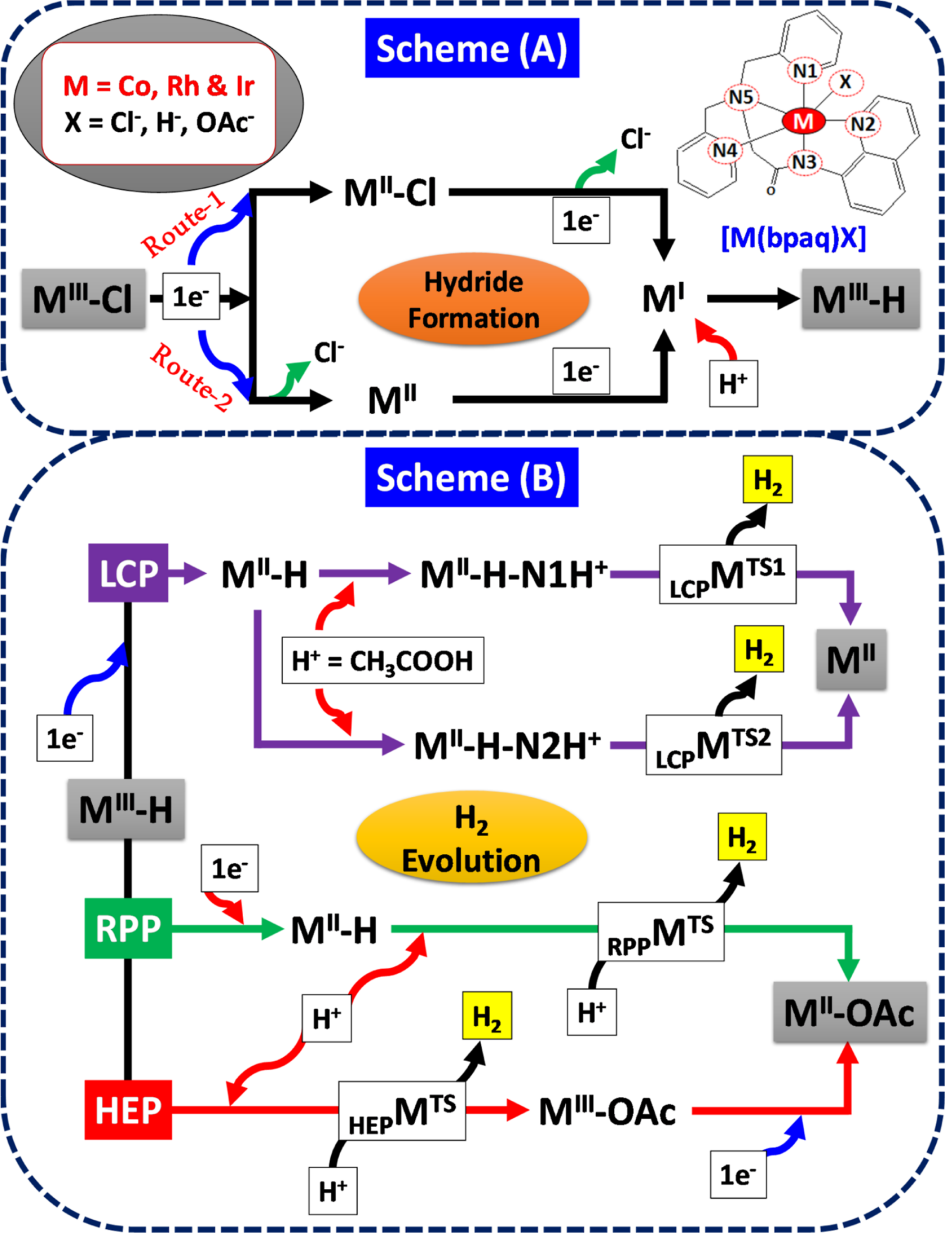
(A) Proposed Reaction Mechanism for the Formation of the metal-hydride (M^III^–H) Species from the M^III^–Cl Species through Route-1 and Route-2; (B) proposed Reaction Mechanism for the HER from metal-hydride (M^III^–H) Species through the LCP Pathway, RPP, and HEP, Involving Reduction and Protonation Steps Facilitated by a Monoanionic Amido Pentadentate (bpaqH) Ligand

The present study focuses on theoretical mechanistic investigations of bioinspired homogeneous catalysts to explore the HER facilitated by bpaqH ligand-based transition metal complexes (Co, Rh, and Ir). Building upon earlier experimental and theoretical studies,^27^Scheme 1 proposes the mechanism for HER involving amido-pentadentate ligand-based Co, Rh, and Ir complexes by exploring all potential pathways. With this context, density functional theory (DFT) calculations were conducted to achieve the following objectives: (i) to determine the reactivity of M^III/II^H species (where M = Co, Rh, and Ir), and (ii) to analyze the structural reactivity of M^III/II^–H species concerning hydrogen production through heterolytic and RPP for HER. Consequently, this work aims to present a comprehensive understanding of the mechanisms within group 9 transition metal-based bpaqH complexes, employing state-of-the-art methods, with a potential to significantly advance the field of HER catalysis.

## 2 Computational Methodology

All theoretical calculations were conducted using the ORCA 5.0.3 program.^59,60^ Validation of our theoretical calculations against available experimental data was performed utilizing both ORCA and Gaussian 09 software.^61^ This validation process has been comprehensively discussed in the Supporting Information, specifically in Sections S1.1 and S1.2 (see the Supporting Information, Figure S1, Tables S1 and S2). The widely recognized B3LYP^62,63^ hybrid functional was employed to analyze bonding properties, with comparisons to single-crystal data provided in Table S1 and redox properties in Table S2 (see Supporting Information). This functional is known for its reliability in predicting structural and redox properties, as well as energetic values.^28−30^ For H, C, N, and O atoms, the 6-31G(d) basis set^64^ was used, while the Los Alamos effective core potential (LANL2DZ)^65,66^ was applied to the metal centers (Co, Rh, and Ir). The choice of the B3LYP functional, along with the 6-31G(d), LANL2DZ, TZVP, and def2-SVP basis sets, is well supported by studies on organometallic compounds, including those with polypyridyl ligand frameworks.^12,22−27,30^ Details on the use of these basis sets are provided in Supporting Information Sections S1.1 and S1.2. The gas-phase-optimized geometries were scrutinized to exhibit real harmonic vibrational frequencies for all intermediates, including possible spin states (see the Supporting Information, Table S3). Transition state geometries were identified with one imaginary frequency, and thermodynamic corrections for free-energy calculations were derived at room temperature. To ensure that the specific transition states connect reactants and products, intrinsic reaction coordinate calculations were conducted.^30^ Solvation effects in acetonitrile were considered through single point calculations performed on gas-phase optimized structures. This was accomplished using the CPCM and C–PCM solvation models with the COSMO epsilon function (CPCMC).^67−70^ Electronic energies were computed using the same functional, employing a larger basis set at the B3LYP/TZVP level.^30,71^ Furthermore, redox properties were calculated from the reduced and oxidized species by using Born–Haber Cycle.^30,72−74^

I. Calculated reduction potentials (from eq 1) and p*K*_a_ values (from eq 2)
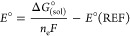
1
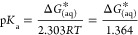
2where Δ*G*° = −*nFE*_0_, with *n* being the number of transferring electrons, *F* is Faraday’s constant (*F* = 96.484 kJ/mol or 23.06 kcal/vol gram), and Δ*G*° is the free energy of reduction with 1 M standard states. Various practical methods are considered for adjusting free energies computed with the ideal gas approximation to better estimate the solution-phase free energies. To account for the 1 M standard state in acetonitrile solution, a concentration correction of 1.89 kcal/mol [RT ln(24.5)] was applied to the computed reaction profiles at room temperature (298 K) based on the gas-phase optimized geometries. All reduction potentials were determined with respect to the saturated calomel electrode (SCE) in acetonitrile.^51^ The Δ*G*° = −[ln(10)RT] p*K*_a_ was used to calculate p*K*_a_’s, where Δ*G*° is the free energy of protonation. The standard state aqueous free energy of a proton, *G*_aq_*(H^+^), was calculated, in which the gas-phase free energy was *G*_g_^°^(H^+^) = −6.29 kcal/mol. Experimentally, the measured hydration free energy [*G*_aq_, solv (H^+^) = −265.9 kcal/mol] was taken from the literature.^75^ Similarly, the SCE reference value (4.522 V) was considered as the reference electrode.^76^ Experimentally, the value of −1.90 V vs SCE was used as the applied potential,^51^ which has also been included for constructing free energy profiles. Furthermore, the calculated heterolytic bond dissociation of six-coordinated monohydride [M(bpaqH)H] complexes were calculated for hydricity values from M^III^–H species in acetonitrile.

II Calculated hydricity values (from eqs 3–7)
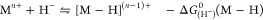
3
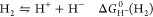
4
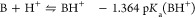
5
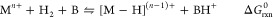
6
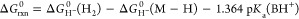
7

Thermodynamic hydricity, (Δ*G*_H^–^_^0^), can be determined by the following method,^77^ where B = base, BH^+^ = conjugate acid of B. The strength of the base necessary to facilitate H_2_ production is determined by hydricity of the metal hydrides^78^ in which the hydricity of H_2_ is Δ*G*_H^–^_^0^(H_2_) = 76.0 kcal/mol in acetonitrile. It is clear that for a metal hydride (M–H) which is capable of undergoing hydride transfer to acetic acid (Δ*G*_H^–^_^0^ = 44 kcal/mol), the added base must have p*K*_a_(BH^+^) > 23.5 in acetonitrile for H_2_ cleavage to be thermodynamically favorable under standard conditions.^79−81^ We calculated for the vertical excitations through the TD-DFT method^82^ to determine the absorption spectra, which was quite comparable with experimental available data (see the Supporting Information, Table S1). In order to study the bonding nature of M^III/II^–hydride species, Mulliken charges from natural bond orbital^83^ and the quantum theory of atoms in molecules (QTAIM) analyses were performed at the same level of theory used for optimization. All QTAIM calculations were performed by using AIM 2000 package.^84^

## 3 Results and Discussion

### 3.1 Explored Catalytic Pathways

The proposed mechanism for proton reduction involving monoanionic amido pentadentate-based molecular electrocatalysts (M = Co, Rh, and Ir) is depicted in Scheme 1A,B. Scheme 1A illustrates potential pathways for transient metal hydride formation from metal(III) chloro [M(bpaqH)Cl] complexes. The formation of M(III) hydride species can occur in two ways: (i) by generating a metal(I) species followed by one electron reduction (Co^III/II^–Cl) and subsequent chloride dissociation or (ii) through one electron reduction coupled with chloride dissociation. These pathways may occur in a concerted or stepwise manner. Moreover, M(III)–H can also be produced via protonation of M(I) species from acetic acid. Once the metal hydride forms, it can follow three predominant pathways: ligand-centered protonation (LCP), RPP, and HEP, directing hydrogen evolution. The entire process can be categorized into three major steps: ligand dissociation, reduction, and protonation, as illustrated in Scheme 1B. The first pathway LCP, (i) LCP pathway, involves H_2_ evolution (_LCP_M^TS^), preceded by protonation to form M(II)–H–N1H^+^ and M(II)–H–N2H^+^, as part of the ligand centered protonation steps. The second pathway, (ii) RPP, entails the evolution of H_2_ (_RPP_M^TS^) preceded by a one-electron reduction step (from M^III^–H to M^II^–H) facilitated by protonation from acetic acid acting as the proton donor. Lastly, (iii) the HEP = Heterolytic pathway involves H_2_ evolution through the heterolytic cleavage of M^III^–H species, preceded by protonation via acetic acid. This section will encompass discussions on the structural and energetic aspects of all species involved in both Scheme 1A,B.

### 3.2 Energetic Analysis of the Main Intermediates toward Metal(III)-Hydride Formation

**Figure 1:**
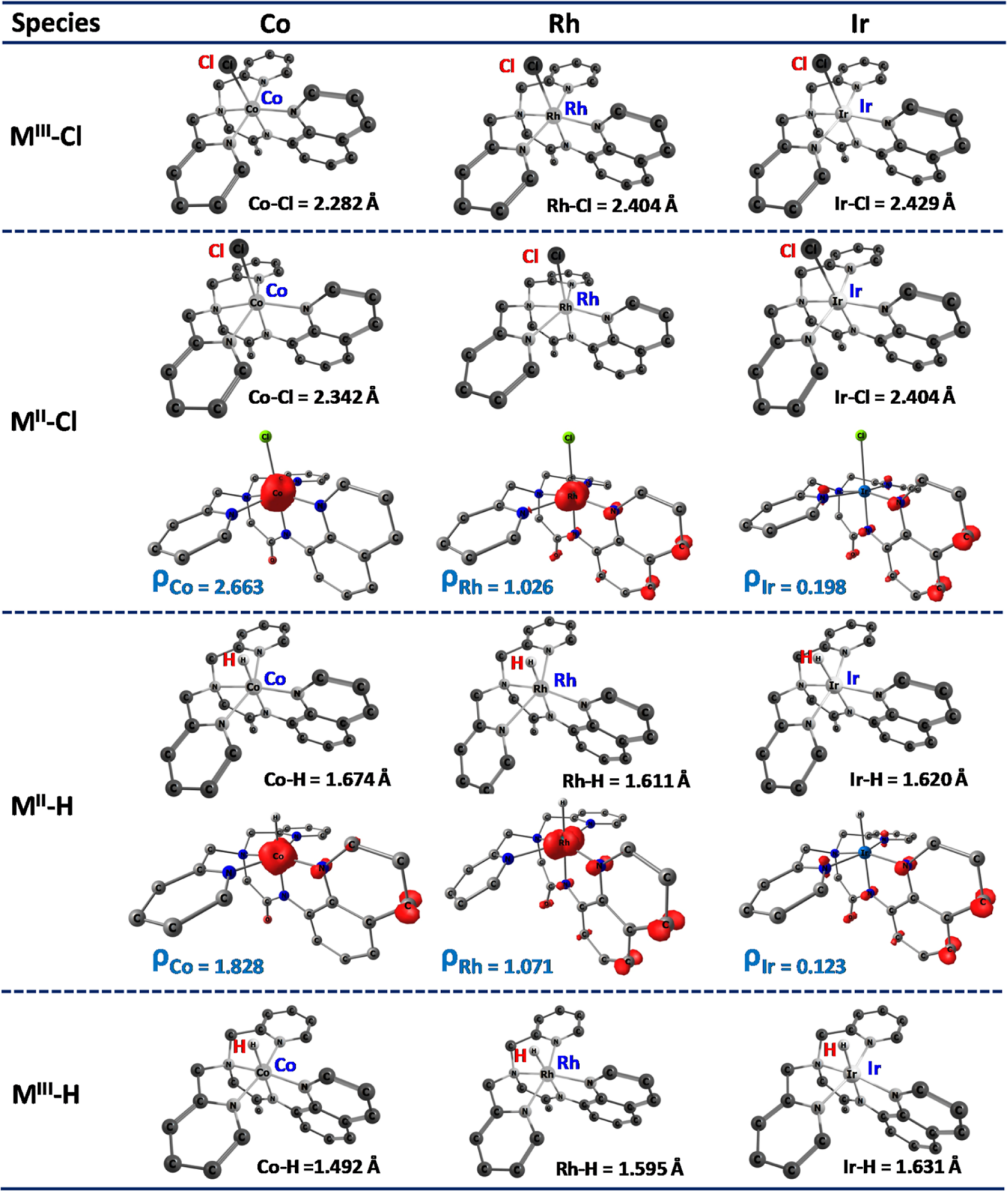
Selected optimized geometries of key intermediates and their spin density plots for both Scheme 1A (metal hydride formation) and Scheme 1B (hydrogen production) of the chosen species, using three different metal systems (Co, Rh, and Ir). For clarity, hydrogen atoms are omitted.

**Figure 2:**
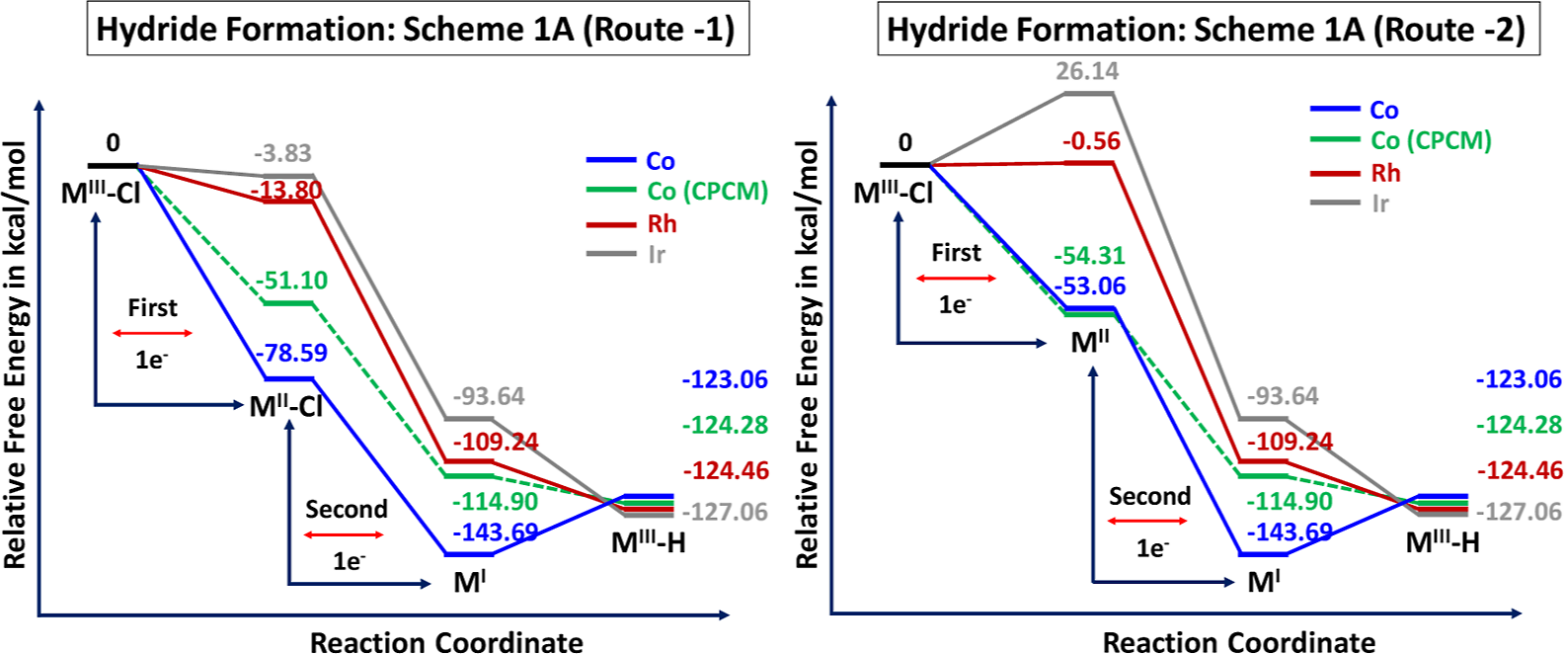
Comparative relative free energies for the first and second reduction steps, as well as protonation steps, involved in the formation of three metal hydride species (M^III^–H, where M = Co, Rh, and Ir) via Route-1 and Route-2 in Scheme 1A from M^III^–Cl species. The estimated energies (kcal/mol) are referenced to the ground state energy of the [M^III^(bpaqH)Cl] complex.

In this discussion, the mechanistic aspects and the impact of the chloride anion on the formation of M(III)–hydride species within metal (Co, Rh, and Ir) chloro [M(bpaqH)Cl] complexes are examined. Optimized geometries alongside their corresponding geometrical parameters (selected bond lengths in Å and spin density) are presented in Figures 1, S2 and S3 (see the Supporting Information). Analysis of the M^III^–Cl complex indicates that the Co–Cl axial bond length (2.282 Å) is longer compared to those of Rh–Cl (2.404 Å) and Ir–Cl (2.429 Å). Calculated bond lengths of M^II^-Cl and M^II^–N_5_ were found to be 2.342 and 2.064 Å for Co, 2.402 and 1.973 Å for Rh, and 2.404 and 1.969 Å for Ir complexes, respectively. Geometrically, all intermediates exhibit contraction of M–N_1–4(equatorial)_ and elongation of M–N_5(axial)_ bond lengths for Co compared to Rh and Ir (see the Supporting Information, Table S4). As we move down the group from 3d^7^ to 4d^7^ and 5d^7^, there is a notable shift in the axial bond lengths of M^II^–Cl, which are shorter compared to M^III^–Cl. Additionally, in the 3d^7^ case, the axial M–N_5_ bonds (in M^II^–Cl species) are significantly elongated compared to 4d^7^ and 5d^7^. However, this trend is reversed in the M^III^–Cl species due to reactivity differences. Figures 1 and S3 (see the Supporting Information) illustrate the spin density plots for all calculated intermediate species from Scheme 1A,B. This pattern is reflected in their spin density values of 2.663 for Co, 1.026 for Rh, and 0.198 for Ir complexes, signifying regular high spin M^II^–Cl (*S* = 3/2) complexes. The substantial reduction of spin density from 2.663 to 0.198 (for M^II^–Cl) demonstrates considerable spin delocalization on the ligand skeleton when moving down the groups (shown in Figure 1), indicating an increased covalency. A similar trend was observed for other selected intermediate species. Moving from 3d^7^ to 5d^7^, the elongation of bond lengths and higher spin density associated with Co^II^–Cl compared to Rh^II^–Cl and Ir^II^–Cl complexes indicates higher reactivity attributed to the ionic nature of the Co–Cl complexes. Figure 2 displays the computed relative free energies for metal hydride formation involving 2 steps (Route 1 & Route 2) (Refer to Scheme 1A). All energies are derived from B3LYP/def2-SVP calculations, incorporating free energy corrections in both gas and acetonitrile (in the CPCM model). The low spin M^III^–Cl complex serves as the initial geometry, whereas the penta-coordinated ([M^II^(bpaqH)]) and hexa-coordinated ([M^II^(bpaqH)Cl]) species are considered more stable complexes. These coordinated species exist in a high spin state and can undergo one-electron reduction to yield reactive M(I) intermediates. According to our findings, the reduction of [Co^III^(bpaqH)Cl] to [Co^II^(bpaqH)Cl] exhibits significant exothermicity, resulting in an energy decrease of −78.59 kcal/mol.

Moreover, comparing this reduction process to the solvent phase (acetonitrile) using the CPCM model reveals an even greater exothermicity. In Figure 2, we observe an additional exothermicity of 27.49 kcal/mol for Route-1 and −1.25 kcal/mol for Route-2 between the gas and solvent models (CPCM) specifically for cobalt. Additionally, the exoergic nature of the reduction from [M^III^(bpaqH)Cl] to [M^II^(bpaqH)Cl] becomes less pronounced as we move down the group. This is clearly depicted in Figure 2 (Route 1), showcasing energy decreases of −13.80 and −3.83 kcal/mol for Rh and Ir, respectively. The calculated Co^III^/Co^II^ couple value stands at −0.324 V vs SCE, closely aligning with the experimental value of −0.330 V. However, the computed redox potential tends to overestimate the experimental range (ranging from 0.150 to 0.200 eV) for small organic molecules and organometallic complexes relevant to electrocatalysis. This discrepancy primarily arises from computational errors being systematically nullified.

The computed reduction potentials for the M^III^ to M^II^ couples are −3.236 and −3.658 V for Rh and Ir, respectively. In Figure 2 (Route-2), the subsequent chlorine dissociation followed by 1e^–^ reduction results in thermodynamically driven formations of Co(I) species at energies of −53.06 and −143.69 kcal/mol, while the formation of Ir(I) species remains endothermic (26.14 kcal/mol). Uncertainty exists regarding the formation of [M^I^(bpaqH)Cl] intermediate from [M^III^(bpaqH)Cl], whether it occurs solely through concomitant 1e^–^ and ligand dissociation or by sequential 1e^–^ reduction followed by ligand dissociation. Between the two intermediates (M^II^–Cl and M^II^), the formation of transient M^II^–Cl species is more exoergic than that of M^II^ species. However, both intermediates are essential in the reduction process, warranting special attention to their electronic structure. Computed results (see the Supporting Information in Table S2) show the reduction potential for the Co^II^–Cl/Co^I^ couple at −1.758 V (vs experimental value of −1.55 V vs SCE), −0.481 V for Rh^II^–Cl/Rh^I^, and −0.549 V for Ir^II^–Cl/Ir^I^ couples, respectively. Similarly, the reduction potentials for Co^III^|Co^II^ and Co^II^|Co^I^ couples tend to be overestimated by 0.006 and 0.205 V, respectively, in the context of monoanionic amido pentadentate ligand systems. These data indicate that the formation of M(I) from M(II)–Cl complexes occurs feasibly either through ligand dissociation or ligand dissociation followed by 1e^–^ reduction. Regarding the energy of protonation steps, corresponding to the reaction [M] + HA → M – H + A^–^, for Co, Rh, and Ir, the protonation of M(I) species leads to the formation of a more reactive Co^III^–H (−123.06 kcal/mol) than the Rh^III^–H and Ir^III^–H intermediates (−124.46 and −127.06 kcal/mol, respectively).

The computed p*K*_a_ values, provided in Table S2 (see the Supporting Information), offer qualitative insights into the acidity of metal-hydride species. For the protonation of Co(I) species, the p*K*_a_ value stands at 6.86, while it is notably higher for Rh(I) and Ir(I) at 34.88 and 45.28, respectively. These values indicate the need for strong acidic conditions in complex systems characterized by lower p*K*_a_ values for efficient hydrogen evolution.^30,85−92^ These findings align with experimental observations, emphasizing that Co(I) catalysts require stronger acids compared with Rh(I) and Ir(I) catalysts for hydrogen production. Subsequently, the reduction of M(III)–H species to form an M(II)–H intermediate occurs through a one-electron reduction with a negative reduction potential (−1.288 V). Rh(III)–H/Rh(II)–H and Ir(III)–H/Ir(II)–H couples exhibit more negative potentials (−4.522 and −4.020 V for Rh and Ir, respectively (see the Supporting Information, Table S2). This suggests that while Co^III/II^–H with a sufficiently negative potential can form a M(II)–H complex, the more negative potentials associated with Rh and Ir complexes make this formation less favorable. Minimizing the overpotential for catalysis requires the difference between the two redox couples (M^III^–Cl/M^II^, M^II/I^ & M^III/II^–H) to be as small as possible.^12,28,39−41^ The computed results show a lower reduction potential difference between the two redox couples for Co compared to Rh and Ir centers (1.431 V and −0.985 V between Co^III/II^ & Co^II/I^ and Co^III/II^ & Co^III/II^–H couples). Consequently, cobalt-based electrocatalysts are more efficient for hydrogen evolution compared with their Rh and Ir counterparts.

### 3.3 Mechanistic Pathway for Proton Reduction

#### 3.3.1 Heterolytic Pathway (M^III^–H) toward H_2_ Evolution

**Figure 3:**
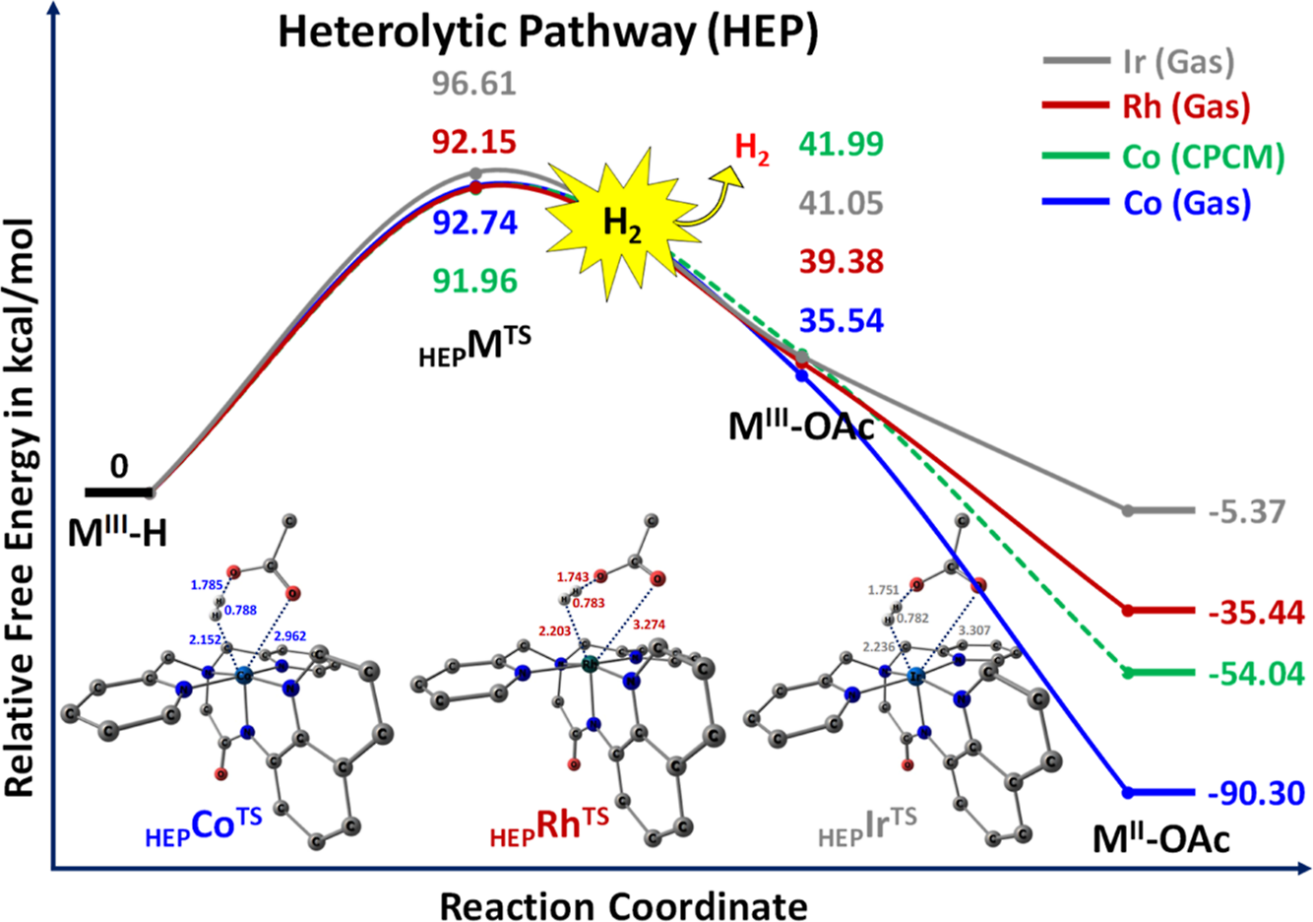
Computed free energy profiles for proton reduction from metal (M^III^–H, M = Co, Rh, and Ir) hydride via HEP along with structural parameters of their corresponding transition state. All the energies are reported here in kcal/mol.

The computed Gibbs free energy profile depicting the heterolytic cleavage of M^III^–H species by acetic acid to yield hydrogen molecules (H_2_) through six-membered (_HEP_M^TS^) transition states is presented in Figure 3. Analyzing the profile, it is evident that the reaction involving Co^III^–H with a proton from acetic acid, leading to H_2_ via heterolytic cleavage of Co^III^–H, presents an endergonic barrier of 92.74 kcal/mol (91.96 kcal/mol in CPCM). Shifting focus to Rh^III^–H and Ir^III^–H systems, the cleavage of hydride (H−) from Rh^III^–H and Ir^III^–H, in concert with H^+^ provided by acetic acid, leads to the formation of H_2_ and _HEP_Rh^TS^, _HEP_Ir^TS^, both endergonic by 92.15 and 96.61 kcal/mol for Rh and Ir, respectively. Interestingly, for cobalt, there is minimal disparity observed between the gas and solvent (acetonitrile) mediums using the CPCM model. Ir-hydride species can also engage with a proton to produce H_2_ through heterolytic rupture.

In Figure 3, the bond length of the coordinated H_2_ in _HEP_Co^TS^ measures 0.788 Å, elongated by 0.044 Å, compared with the standard dihydrogen bond length (0.740 Å). Moreover, _HEP_Co^TS^ was identified as a late “product-like” transition state, showcasing initial H–H and Co–OAc bond distances of 0.788 and 2.962 Å, respectively. Similar trends were observed for Rh and Ir systems in Figure 3, indicating comparable transition states for the metal hydride (M–H) bond, H–H formation, and O–H bond distances in acetic acid across Co, Rh, and Ir. This analysis highlights similar activation barriers in the HEP pathway for all transition states. Subsequently, acetate attachment forms Co^III^–OAc (35.54 kcal/mol), which then reduces to a more stable intermediate of Co^II^–OAc (−90.30 kcal/mol). Overall, the computed results indicate that M^III^–H species exhibit high activation barriers for proton reduction involving the bpaqH scaffold via heterolytic means compared to those of Rh and Ir systems. Hence, hydrogen production occurs with less facile barriers for all Co, Rh, and Ir complexes in the HEP pathway.

#### 3.3.2 RPP (M^III/II^–H) toward H_2_ Evolutions

**Figure 4:**
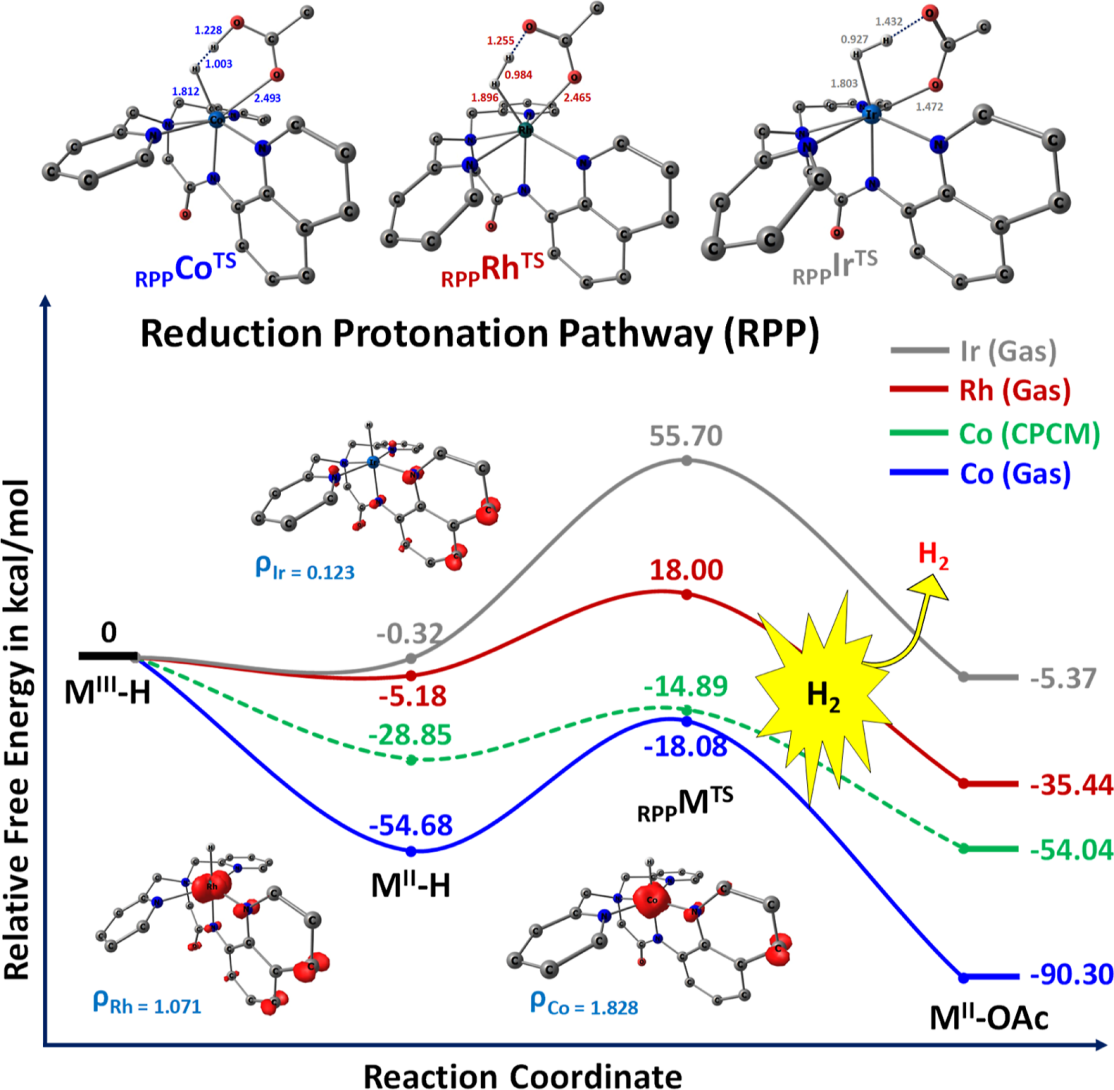
Computed free energy profile for proton reduction from metal hydride (M^III^–H) intermediates via RPP. All energies are reported in kcal/mol. Inset shows the structural bond parameters depiction with the transition (_RPP_M^TS^) state geometries. _RPP_M^TS^: suffix RPP implies RPP, superscript TS is transition state, and M indicates metals (Co, Rh, and Ir).

Figure 4 presents an energetic outline of electrocatalytic hydrogen evolution via the RPP in bpaqH ligand-based Co, Rh, and Ir complexes. The reaction initiates with one-electron reduction of M^III^–H, leading to the formation of the exoergic M^II^–H species, which facilitates the evolution of H_2_ molecules. The computed reduction potential values for M^III^–H/M^II^–H couples are detailed in Table S2 (see the Supporting Information). Despite the absence of experimental values, the redox potential aligns with values related to other ligand structures. Furthermore, subsequent reduction steps exhibit even more negative potential, substantiated by the electronic distribution of the Frontier MOs in the quartet state of [M(bpaqH)H]^1+^ compared to the singlet state of [M(bpaqH)H]^2+^ (see the Supporting Information, Figures S4 and S5). In this study, M^II^–H (quartet state) is considered the ground state, with an energy difference of 4.31 kcal/mol between high (quartet) and low (doublet) spin states. Notably, M^II^–H prefers a high spin state (quartet) with distinct spin density values for cobalt, rhodium, and iridium complexes (see Figure 4 and Supporting Information, Table S5). Irrespective of the system, iridium exhibits lower spin density on its hydride moiety (0.123), indicating a comparatively reduced metal hydride cleavage ability compared to cobalt and rhodium. The higher activity of cobalt and rhodium complexes is attributed to weak σ-bond contributions and lower hydride donor abilities, as expounded in Section 3.3.4. The computed energy profiles depict the exoergic nature of the reduction from Co^II^I–H to Co^II^–H by −54.68 kcal/mol with a one-electron reduction potential of −1.288 V vs SCE. The Rh^III^–H to Rh^II^–H reduction appears energetically more stable than Ir^II^–H by −5.18 kcal/mol, the latter being slightly less exoergic by −0.32 kcal/mol, exhibiting a reduction potential of −4.020 V. Following this, protonation of M(II)–H species using acetic acid reveals an estimated activation barrier of 36.60 kcal/mol (13.96 kcal/mol in CPCM), resulting in the formation of the highly exoergic Co^II^–OAc species (−90.30 kcal/mol). Although the activation barrier is relatively lower for Rh and Ir metal centers, the stability of the M^II^–OAc species (M = Rh, Ir) is slightly inferior to that of the Co^II^–OAc species (−35.44 and −5.37 kcal/mol, respectively).

Consequently, among the three metals, Co–H and Rh–H demonstrate superior efficiency in the RPP compared to that of Ir–H. As per Figure 4, the transition states for the metal hydride (M–H) bond distances are determined to be 1.812 Å for Co, 1.896 Å for Rh, and 1.803 Å for Ir. This analysis demonstrates that CO and Rh exhibit more favorable M–H cleavage compared to Ir. Additionally, upon protonation of M–H by acetic acid, the formation of H–H bonds reveals bond lengths of 1.003 Å for Co, 0.984 Å for Rh, and 0.927 Å for Ir. In terms of the bonding nature of M^III/II^-hydride species, it is observed that the Co–H and Rh–H bonds are significantly weaker compared with the Ir–H species. Remarkably, for a similar O–H bond distance (0.973 Å) from CH_3_COOH, the RPP mechanism shows smaller distances, indicating its higher efficacy in bringing protons (from CH_3_COOH) together to form H_2_. Subsequently, M^II^–H undergoes protonation, leading to the thermodynamically favorable formation of M^II^–OAc intermediates. The hydride donor ability of M(III/II)–H is directly correlated with more negative or less positive Mulliken charges on hydride species.77–79 Calculated Mulliken charges of hydrides (from Co–H, Rh–H, and Ir–H) are −0.308, −0.145, and −0.089, respectively. Thus, the trend of decreasing negative charges on MII-H follows the order Co–H > Rh–H > Ir–H, as detailed in Table 1. The hydride (H^–^) ion of M^II^–H, being less electropositive than M^III^–H, enhances the nature of heterolytic cleavage of the M–H bond to release hydride ions. Quantitative analysis of Mulliken charges underscores their direct relationship with the cleaving nature of the metal-hydride (M–H) bonds, impacting various other properties like hydricity, dipole moment, redox properties, and electronic structure. Likewise, the redox process might be facilitated through multiconfiguration states, as demonstrated in the computational analysis of redox couples involving metal-oxo/hydroxo complexes and other species.^93^

**Table 1:** Computed Mulliken Atomic Charges (in Atomic Units) on Selected Species of the Metal Hydride (Co–H, Rh–H, and Ir–H) Species

	Co			Rh			Ir		
species	metal	hydride	M–H	metal	hydride	M–H	metal	hydride	M–H
M^III^–H	0.334	–0.103	0.231	0.315	–0.113	0.202	0.398	–0.071	0.327
M^II^–H	0.495	–0.308	0.187	0.289	–0.145	0.144	0.467	–0.089	0.378

#### 3.3.3 LCP Pathway (M^III/II^–H) toward H_2_ Evolution

**Figure 5:**
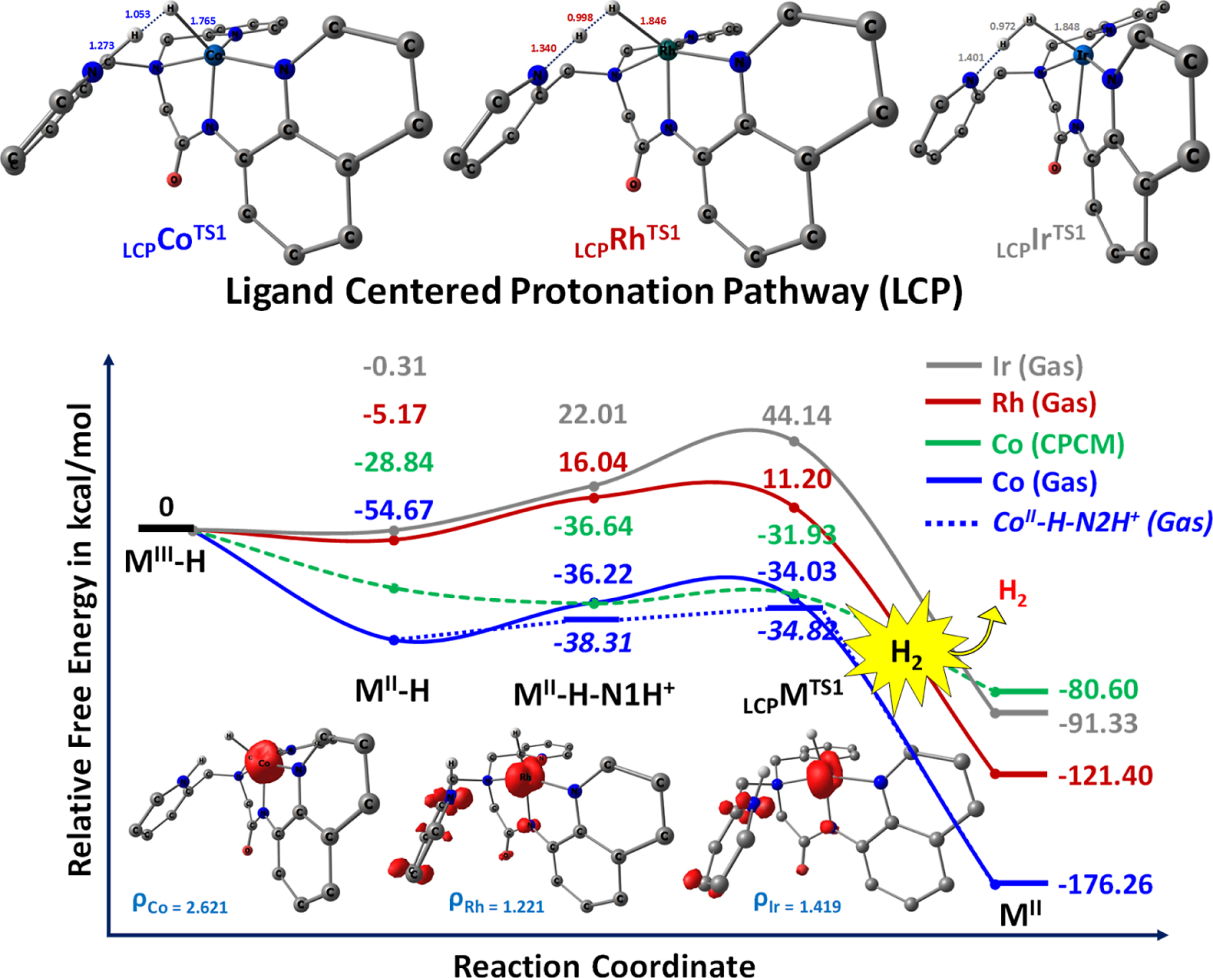
Computed free energy profiles for ligand centered protonation from metal hydride (M^III^–H) intermediates via LCP pathway. All energies are reported in kcal/mol. Inset shows the structural bond parameters depiction with the optimized transition (_LCP_M^TS^) states and the spin density plots for their respective intermediate (M^II^–H) species. _LCP_M^TS^: suffix LCP implies ligand centered protonation pathway, superscript TS is transition state, and M indicates metals (Co, Rh and Ir).

During the LCP pathway, which triggers the evolution of H2 (LCPMTS), protonation initiates the creation of M^II^–H–N1H^+^ and MII–H–N2H^+^ species. This step precedes LCP and has been a focus of previous studies aimed at to delving into its mechanisms across various systems for deeper insights.^94−97^ The free energy profile diagram delineating proton reduction through the LCP process via two equatorial nitrogens from pyridine nitrogen (N1) and aminoquinoline (N2) atoms for protonation in the [M^III^(bpaqH)H] complexes is displayed in Figure 5. The formation energies of the LCP process stand at −36.22 kcal/mol for MII–H–N1H^+^ and −38.31 kcal/mol for MII–H–N2H^+^ complexes, fairly comparable across both ligand-centered protonated systems. It is noted that the formation of Co^II^–H–N1H^+^ is thermodynamically more favorable by approximately −31.93 kcal/mol (_LCP_Co^TS^) compared to that of the Rh and Ir species.

Following this, M^II^–H–N1H^+^ interacts with a proton from acetic acid to produce the H_2_ molecule and form M^II^ species, with corresponding p*K*_a_ values of 5.37 for Co^II^–H–N2H^+^ and 5.72 for Co^II^–H–N1H^+^ species. In Figure 5, the kinetic barrier for the _LCP_M^TS1^ step is estimated as −34.03, 11.20, and 44.14 kcal/mol for Co, Rh, and Ir, respectively. The nature of transition states is confirmed to possess only one imaginary frequency of 943.0*i*, 1009.9*i*, and 1085.4*i* cm^–1^ for Co, Rh, and Ir, respectively, along with their corresponding force constants of 0.54 mdyn Å^–1^ for Co, 0.62 mdyn Å^–1^ for Rh, and 0.82 mdyn Å^–1^ for Ir (see the Supporting Information, Table S6). Evidently, the activation barrier associated with the LCP mechanism is notably lower for Co than for Rh and Ir. However, the computed free energy profile of the reaction at 298 K displays a slightly lower activation barrier height of 3.49 kcal/mol for _LCP_Co^TS^ concerning the M^II^–H–N2N^+^ complex. This notably indicates the thermodynamic favorability of H_2_ formation for proton reduction under these conditions. Overall, these computed results underscore the existence of a ligand-centered pathway originating from two protonation steps leading to H_2_ formation. Thus, the LCP pathway can also be considered a favorable route over HEP for hydrogen production involving bpaqH ligand systems.^51^ This implies that the LCP pathway in the HER is not sequential but concerted, thus avoiding high-energy intermediates. Based on the activation barriers in the LCP process, it is observed that Co and Rh catalysts are more efficient than Ir catalysts in this specific pathway.

#### 3.3.4 Thermodynamic Hydricity and p*K*_a_ Values of Transition Metal (Co, Rh, and Ir) Hydrides

**Figure 6:**
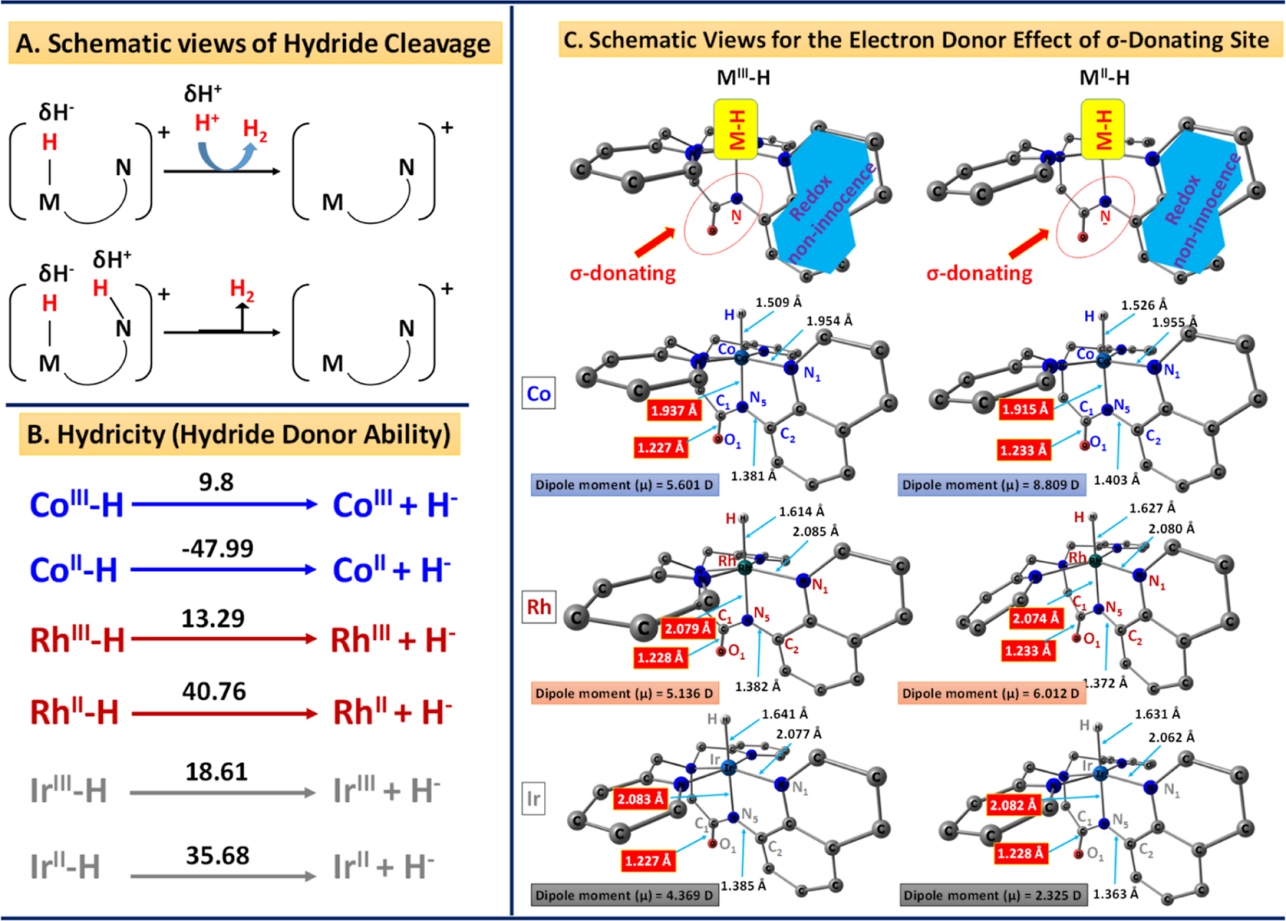
Schematic views of the (A) cleavage mechanism involving metal-hydride species (M as the metal site and N as the nitrogen atom), (B) hydricity values in kcal/mol), and (C) electron donor effect from σ-donating site along with the selected geometrical bond lengths (Å) and dipole moments (μ) for metal-hydride (M^III/II^–H) species for hydrogen evolution.

Following the methodology outlined by DuBois and colleagues, experimental thermodynamic values (Δ*G*°H^–^) for the heterolytic cleavage of an M–H bond were provided.^39,52,53,77−79^ The magnitude of the hydricity value (Δ*G*°H^–^) signifies the energy required for the M–H bond cleavage. Higher values of Δ*G*°H^–^ suggest weak donating ability of hydrides, while smaller values indicate strong hydride donating ability of Metal-Hydrides. In this context, M(I) necessitates at least 30 kcal/mol less energy for hydride cleavage, and metal hydride must have Δ*G*H^–^ greater than 44 kcal/mol, crucial for releasing H_2_ using acetic acid as the proton source.^77−79^ The calculated hydricity of M^III^–H species stands at 9.87, 13.29, and 18.61 kcal/mol for Co, Rh, and Ir, respectively (see Figure 6B). Therefore, the Co^III^–H intermediate exhibits greater donor ability than Rh and Ir hydride species due to its smaller values of Δ*G*°H^–^. Similar trends in the donating ability were observed in the hydricity values of MII–H species. The Δ*G*°H^–^ for Co^II^–H species is estimated at −47.99 kcal/mol, whereas the other two metal hydrides (Rh and Ir) have positive Δ*G*°H^–^ values of 40.76 and 35.68 kcal/mol for Rh^II^–H and Ir^II^–H species, respectively. Lower values of Δ*G*°H^–^ correspond to an abundance of hydride donors and lower p*K*_a_ values, indicating stronger acids.^53^ This suggests that the hydride-donating ability is stronger in Co(II)–H than in Co(III)–H species, enabling hydrogen generation with lower activation barriers through Co(II)–H intermediates rather than in Co(III)–H species. Protonation seems to precede respective reduction potentials in the strong acidic medium employed for the experiments.^98^ Furthermore, the acidity of group 9 transition elements diminishes as the group moves down, evident in the computed p*K*_a_ value of protonated M(I) species. The calculated p*K*_a_ value of protonation for Co(I) species is 6.86 in acetonitrile medium, while it stands at 34.88 and 45.28 for Rh(I) and Ir(I) species, respectively (see the Supporting Information, Table S2). Additionally, the acidity of metal hydrides with 6 coordination numbers follows the order of the first row > second row > third row, consistent with earlier reports.^53,80,99,100^ The π-acidic nature of the σ-donating C=O group augments the cleavage ability of the Co–hydride bond over Rh–H and Ir–H bonds. This is evident from the higher dipole moment values of Co–H species compared to the other two metal hydrides^101^ (see Figure 6C). This charge separation/polarity is likely mitigated through the electron-donating nature of the C=O group and noninnocence redox-active aminoquinoline ligand moiety. The discernible charge separation is evident in the distorted octahedral geometry, which is distinctly reflected in their structural parameters. Notably, the M–N_5_, M–H, and C_1_–O_1_ bond lengths appeared more elongated for Rh–H and Ir–H compared to Co–H species (see the Figure 6C). The influence of the σ-donating C=O group in cobalt hydride species amplifies the thermodynamic feasibility of hydride cleavage compared with Rh and Ir hydrides. The distinctive presence of the C=O group in the bpaqH ligand framework significantly facilitates proton reduction, particularly in the more acidic cobalt-hydride species compared to the Rh and Ir hydride species. In summary, the interplay of electronic effects from the ligand skeleton, along with hydricity and p*K*_a_ values, provides a rationale for the heightened reactivity of cobalt-based electrocatalysts over their Rh and Ir counterparts.

#### 3.3.5 Cleaving Ability of Co^III^–H vs Co^II^–H and Co^II^–H–NH^+^ Species toward Hydrogen Evolution in the QTAIM Study

**Figure 7:**
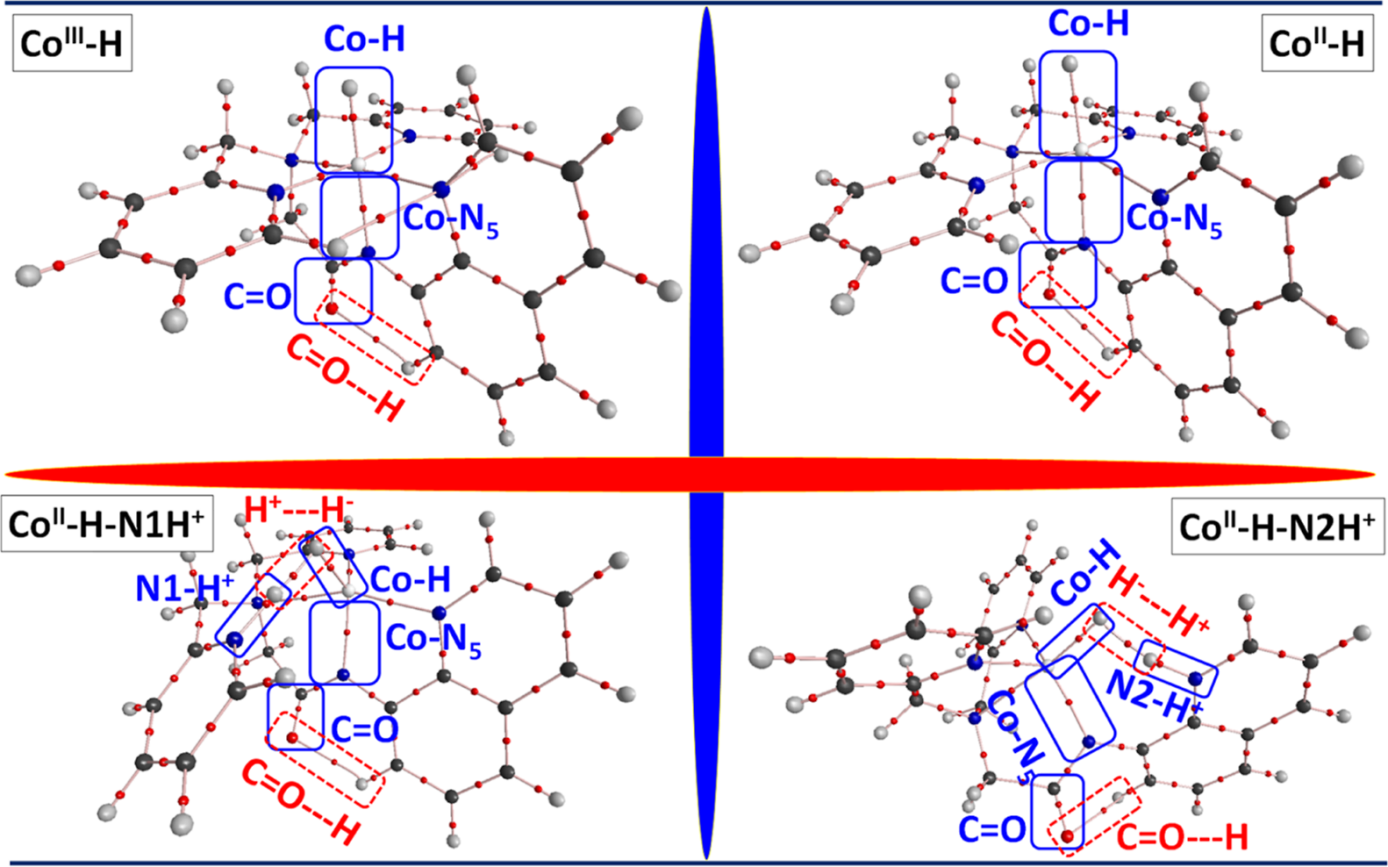
Molecular graph along with QTAIM based topological properties at the selected BCP for the four cobalt-hydride intermediates.

A comprehensive topological analysis using Bader’s QTAIMs^102,103^ was conducted on cobalt hydride species to provide further insights into the strength of metal hydride bonds and associated weak interactions.^30,104−106^ This involved measuring key topological properties such as electron density [ρ(r)] and Laplacian electron density [*L*(r)] at the bond critical point (BCP), aimed at assessing the nature and strength of the Co-Hydride bond. Additionally, other topological parameters such as Hessian values (λ1, λ2), local potential energy density *V*(*r*), and local gradient kinetic energy density at the BCPs were computed to understand the intermolecular interactions within the Co^III/II^–H species (see Figure 7 and Table 2). The computed electron density ρ(*r*) value at the Co^III/II^–H BCP highlights that the CoIII–H σ-bond is stronger than the CoII–H σ-bond, indicated by the highest ρ(*r*) value (0.1322), less negative *L*(*r*), and more positive *G*(*r*) values (−0.469 and 0.1016) [conversely, the ρ(*r*), *L*(*r*), and *G*(*r*) values for the Co^II^–H bond are determined as −0.0496, 0.0722, and 0.0948, respectively]. Additionally, a robust H-bonding interaction (C=O···H) was observed between the aminoquinoline and the π-acidic nature of the σ-donating C=O group.

**Table 2:** Calculated Topological Properties for all the Cobalt-Hydride Complexes Such as Electron Density ρ(*r*), Laplacian of Electron Density *L*(*r*), Hessian Values (λ_1_, λ_2_), *V*(*r*), and *G*(*r*) from QTAIM Analysis^a^

complexes	bonds	ρ(*r*)	*L*(*r*)	*G*(*r*)	*V*(*r*)	λ_1_	λ_2_	λ_1_/λ_2_-1	*V*(*r*)/*G*(*r*)
Co^III^–H	Co–H	0.1322	–0.0469	0.1016	0.1564	–0.2194	–0.2102	0.0436	1.5386
	Co–N_5_	0.0995	–0.1289	0.1421	0.1553	–0.1128	–0.1043	0.0818	1.0929
	C=O	0.4032	–0.0342	0.7452	1.4563	–1.0861	–0.9946	0.0920	1.9541
	C=O···H	0.0136	–0.0110	0.0105	0.0099	–0.0135	–0.0117	0.1588	0.9459
Co^II^–H	Co–H	0.0867	–0.0496	0.0722	0.0948	–0.1253	–0.1031	0.2153	1.3133
	Co–N_5_	0.0662	–0.0850	0.0858	0.0866	–0.0852	–0.0656	0.3002	1.0092
	C=O	0.3931	–0.0193	0.7079	1.3964	–1.0400	–0.9693	0.0730	1.9728
	C=O···H	0.0194	–0.0154	0.0151	0.0147	–0.0207	–0.0189	0.0950	0.9759
Co^II^–H–N1H^+^	Co–H	0.0813	–0.0561	0.0750	0.0940	–0.1101	–0.0978	0.1251	1.2524
	Co–N_5_	0.0872	–0.1163	0.1230	0.1298	–0.1136	–0.1051	0.0805	1.0551
	C=O	0.3994	–0.0337	0.7355	1.4374	–1.0730	–0.9875	0.0866	1.9542
	N1–H^+^	0.2391	0.2262	0.0541	0.3344	–0.8873	–0.8715	0.0181	6.1829
	C=O···H	0.0193	–0.0156	0.0151	0.0146	–0.0208	–0.0189	0.1038	0.9681
	H^+^···H^–^	0.0698	–0.0021	0.0251	0.0481	–0.1261	–0.1191	0.0593	1.9156
Co^II^–H–N2H^+^	Co–H	0.0863	–0.0584	0.0802	0.1019	–0.1128	–0.1073	0.0516	1.2709
	Co–N_5_	0.0683	–0.0898	0.0906	0.0914	–0.0749	–0.0688	0.0882	1.0087
	C=O	0.4037	–0.0383	0.7504	1.4625	–1.0861	–1.0062	0.0794	1.9490
	N2–H^+^	0.2771	0.3043	0.0508	0.4059	–1.1055	–1.0856	0.0183	7.9903
	C=O···H	0.0177	–0.0181	0.0159	0.0137	–0.0165	–0.0094	0.7576	0.8623
	H^+^···H^–^	0.0552	–0.0095	0.0230	0.0365	–0.0894	–0.0094	8.4940	1.5864

a All topological parameters are given in atomic units (a.u).

This interaction was reflected in higher electron density ρ(*r*) values observed for Co^II^–H (0.0194), Co^II^–H–N1H^+^ (0.0193), and Co^III^–H–N2H^+^ (0.0177) species compared to those of the Co^III^–H (0.0136) species. Furthermore, the electron density ρ(r) values of the incipient H^+^···H^–^ bond in Co^II^–H–N1H^+^ and Co^III^–H–N2H^+^ species were computed at 0.0698 and 0.0552, respectively. Moreover, the other two metal-hydride species (Co^II^–H–N1H^+^ and Co^II^–H–N2H^+^) displayed lower values of ρ(*r*), *L*(*r*), *G*(*r*), and *V*(*r*) compared to the Co^III^–H species. These variations contribute to stabilizing the transition states, facilitating robust hydrogen production through a thermodynamically driven LCP pathway. Upon evaluation of the bonding nature of Co^III/II^–hydride species, it is evident that the Co^II^–H bond is weaker than the Co^III^–H bond, indicating the prominent heterolytic cleaving ability of the Co^II^–H bond to form H_2_. Similarly, the heterolytic cleavage of the Co^II^–H–N1H^+^ and Co^II^–H–N2H^+^ species also appears to be more favorable.

#### 3.3.6 Essential Factors Favoring Catalytic Transformations: Structural Differences, Specific Ligand–Metal Interactions, and Practical Implications

The unique catalytic behavior of Co, in contrast to Rh and Ir, can primarily be attributed to differences in ligand–metal interactions, particularly regarding the bond strength, coordination geometry, and electronic properties.

##### • Bond Strength

Cobalt typically forms weaker metal–ligand bonds than Rh and Ir, which promotes faster ligand exchange and facilitates catalytic transformations that require rapid ligand turnover.

##### • Coordination Geometry

Co complexes exhibit greater flexibility in coordination geometry due to their smaller atomic radius and preference for lower coordination numbers. This flexibility provides access to intermediate structures that are often less attainable for Rh and Ir.

##### • Electronic Properties

Cobalt demonstrates a higher degree of electron-sharing with ligands compared to Rh and Ir, enhancing interactions such as σ-back-bonding with specific ligands, which are crucial for its catalytic activity. Overall, these factors collectively contribute to cobalt’s distinctive reactivity, allowing for catalytic transformations that can be less favorable for Rh and Ir.

## 4 Conclusions

The current investigation delves into the mechanistic intricacies of proton reduction using monoanionic amido pentadentate ligand-based metal [M^III^(bpaqH)Cl]^1+^ complexes in an acetonitrile environment (M = Co, Rh, and Ir). Various redox and protonated states of the monoanionic amido pentadentate ligand were explored, focusing on key transition states and intermediates across conceivable pathways for generating metal hydride (M–H) species and proton reduction through HEP, RPP, and LCP routes. The findings include the following.

(i) Protonation of the five-coordinated Co^I^ species appears more feasible within the [Co(bpaqH)]^1+^ species compared to the Rh and Ir complexes.
(ii) The first reduction potential of the M^III^–L/M^II^ couple shows a less negative potential for Co than for Rh and Ir complexes.
(iii) The rapid protonation of Co^I^ followed by the one-electron reduction leads to the highly reactive Co^III^–hydride, demonstrating higher reactivity than Rh^III^–H and Ir^III^–H species.
(iv) The computed results indicate a barrier-less process of H_2_ evolution toward Co^II^–H species for proton reduction in RPP and LCP pathways.
(v) The subsequent steps involve H_2_ release and catalyst regeneration via a thermodynamically favorable pathway.

Moreover, the computational findings emphasize the role of proton relay in facilitating the HER through RPP and LCP catalytic pathways. Comparing cobalt, rhodium, and iridium catalysts in RPP and LCP processes demonstrates that cobalt and rhodium catalysts exhibit higher catalytic performance in molecular hydrogen production than the iridium ligand scaffold. The proton insertion method employed herein enhances the catalytic HER, offering a promising approach to design and optimize homogeneous electrocatalysts for renewable energy applications. This study reveals the thermodynamic and kinetic aspects of transition metal (nd^7^) based molecular electrocatalysts, providing valuable insights for future catalyst design endeavors.

## Supporting Information Available

Comparison between the calculated and available structural experimental data and conceptual MO-diagram of electronic distribution in the singlet state of [Co(bpaqH)Cl]^2+^, additional analyses of the three redox species by considering all possible catalytic pathways, computed optimized geometries of Co, Rh, and Ir complexes along with structural parameters, computed spin density plot, natural atomic charges and stretching frequencies for all the transition states involving with HEP, RPP, and LCP pathways, electronic distribution in the Frontier MOs in [M(bpaqH)H]^2+^ and [M(bpaqH)H]^1+^ metal hydride species, and Cartesian coordinates for all the species of bpaqH based group 9 transition metal complexes (PDF)
