# Supplementary material for: Computational Study on the Proton Reduction Potential of Co, Rh, and Ir Molecular Electrocatalysts for the Hydrogen Evolution Reaction
Source: ACS Omega. 2024 Nov 26;9(49):48766–80. doi: 10.1021/acsomega.4c03260 (PMC11635521; doi:10.1021/acsomega.4c03260)
Supplement: Supplementary file 1 — ao4c03260_si_001.pdf [file ao4c03260_si_001.pdf]

Supporting Information

Computational Study on the Proton Reduction Potential of Co, Rh, and Ir Molecular Electrocatalysts for the Hydrogen Evolution Reaction

Murugesan Panneerselvam[1], *Madhavan Jaccob*[2*] *and Luciano T. Costa*[1*]

[1]MolMod-CS - Instituto de Química, Universidade Federal Fluminense, Campos de Valonginho s/n, Centro, Niterói 24020-14, Rio de Janeiro, Brazil.

[2]Department of Chemistry and Computational Chemistry Laboratory, Loyola Institute of Frontier Energy (LIFE), Loyola College, Chennai – 600 034, Tamil Nadu, India.

Email: ltcosta@id.uff.br & madhavanjack05@gmail.com

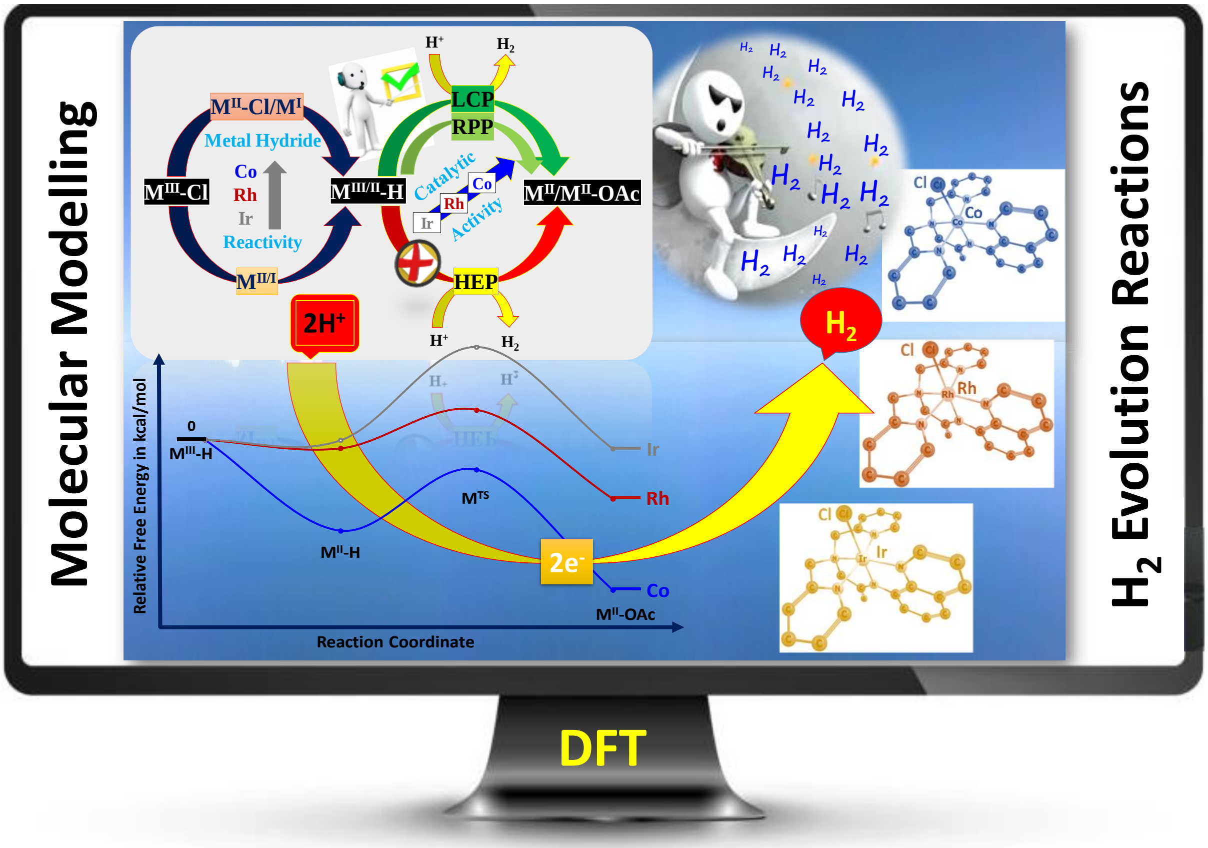

This study explores proton reduction mechanisms in $[M^{III}(bpaqH)Cl]^{1+}$ complexes (M = Co, Rh, Ir) using density functional theory. Key findings include cobalt's superior catalytic performance through efficient protonation and H_2 release, highlighting favorable thermodynamics in reduction-protonation and ligand-centered pathways. Insights inform future catalyst designs for renewable energy applications.

Section S1.1 Comparison between the Calculated and available structural Experimental data for a starting geometry of $[Co(bpaqH)Cl]^{2+}$

The starting geometry of the $[Co(bpaqH)Cl]^{2+}$ complex was structurally identified by single-crystal X-ray diffraction and also analyzed by various spectroscopic studies experimentally.[1] Our computed structural and spectroscopic parameters are in good agreement with the X-ray crystallographic geometrical data and experimental infrared (FT-IR) and UV-visible spectral studies. **Table S1** shows an evaluation of crystallographic Co-N distances, Cl-Co-N bond angles and metric data considered with the B3LYP functional by using ORCA and Gaussian programs. The B3LYP functional[2] was found to be in good agreement with the experimental crystal data and spectroscopic studies (**Table S1**). The structural comparison has been performed in terms of bond lengths (Å) and bond angles (θ, triangles) between Co-N bound distance and Cl-Co-N triangles and they correspond to Cs symmetry compared with experimental results, which is clearly given in **Table S1**. A comparison of the results of the present work show that the B3LYP functional slightly overestimates bond distances (using Gaussian 09 and ORCA programs) when compared with experimental XRD results. It should be noted that the geometry in solid state is not essentially the same as in a solution medium. At the same time, the angle θ is quite reliably reproduced by the B3LYP functional while using ORCA than Gaussian 09. Among the two functionals such as B3LYP/6-31G(d)/LANL2DZ and B3LYP/def2-SVP is in reasonable agreement with experiment results and delivers a good geometric description of $[Co(bpaqH)Cl]^{2+}$ complex. Consequently, the B3LYP functional was employed to run the calculations of geometry optimizations and frequencies, with the chosen two basis sets B3LYP/6-31G(d)/LANL2DZ (using Gaussian 09) and B3LYP/def2-SVP (using ORCA). Also, the choice of B3LYP functional and LANL2DZ & def2-SVP basis sets are better choice to be supported by several studies of organometallic compounds including polypyridyl ligand skeletons.[2-9] Additionally, from the FT-IR spectra of $[Co(bpaqH)Cl]^{2+}$ ground state geometry at gas phase, the C=O stretching vibrational peak was observed at 1625.4 cm^{-1} when computed (at B3LYP/6-31G(d)/LANL2DZ) using Gaussian 09 and 1624.64 cm^{-1} (at B3LYP/def2-SVP) using ORCA, which were quite similar with experimental vibrational band at 1624 cm^{-1}. Moreover, the UV-Visible spectra of $[Co(bpaqH)Cl]^{2+}$ was found to show an intense peak at λ_{max} = 394.78 nm (f_0 = 0.002) and a broad peak at 461.06 nm (f_0 = 0.099) in acetonitrile solvent.

Table S1. Comparison between computed and available experimental data's (Structural XRD, FT-IR and UV-Visible for spectral studies) for a starting geometry of $[Co(bpaqH)Cl]^{2+}$ and performed by using ORCA at B3LYP/def2-SVP and Gaussian at B3LYP/6-31G(d)/LANL2DZ) values are given in brackets [].

Studies	Computation				Experimental		
**Optimized Geometry vs XRD**	**Bond distances [Å]**						
	Co-Cl	2.282 [2.282]			**2.264**		
	Co-N_1	1.976 [1.972]			**1.936**		
	Co-N_2	1.998 [1.991]			**1.945**		
	Co-N_3	1.902 [1.895]			**1.883**		
	Co-N_4	1.965 [1.966]			**1.928**		
	Co-N_5	1.977 [1.972]			**1.942**		
	**Bond Angles [°]**						
	Cl-Co-N_1	89.60 [89.70]			**89.66**		
	Cl-Co-N_2	94.10 [93.98]			**94.81**		
	Cl-Co-N_3	179.30 [179.20]			**178.09**		
	Cl-Co-N_4	95.50 [95.31]			**94.49**		
	Cl-Co-N_5	89.63 [89.70]			**89.55**		
**FT-IR**	**Frequencies**	**Peak Intensity**		**Force Constant**	**Frequencies**	**Peak Intensity**	
	3245.8 [3268.9]	2.96		6.87	**3359**	**Broad**	
	1624.6 [1625.4]	35.10		9.29	**1624**	**Strong (C=O)**	
	1504.9 [1498.6]	27.91		2.63	**1500**	**Week**	
	1458.0 [1439.7]	91.69		2.17	**1459**	**Medium**	
	1410.7 [1400.1]	38.12		4.19	**1400**	**Medium**	
	1243.7 [1237.5]	0.01		1.19	**1234**	**Week**	
	1114.4 [1108.7]	1.98		2.37	**1110**	**Week**	
	1080.6 [1074.1]	0.30		1.98	**1084**	**Week**	
	942.6 [944.4]	0.56		2.92	**939**	**Week**	
	839.0 [837.3]	10.14		1.88	**828**	**Medium**	
	782.9 [784.9]	60.08		0.48	**773**	**Strong**	
**UV-Visible**	**λ_{max}**	**eV**	**f_0**	**% Assignment**	**λ_{max}**	**Peaks**	**Transition**
	394.78	3.14	0.002	95.2%	**388**	**Intense**	**LMCT**
	461.06	2.68	0.099	33.6%	**485**	**Broad**	**d-d**

These peaks were found to arise from the ligand to metal charge transfer (LMCT) and d-d transitions, respectively. These results are concurrent with the experimental evidences and are presented in **Table S1,** which indicates that, from a computational perspective, the trend in choice of two basis sets were not affected by using both the programs (Gaussian 09 and ORCA). Therefore, the full computational study of the overall mechanisms (Scheme A and B) were performed considering the two basis sets such as B3LYP/6-31G(d)/LANL2DZ (using Gaussian

09) and B3LYP/def2-SVP (ORCA). **Table S1** could be clearly indicating that these computed results are in good agreement with an experimental available data. The starting geometry of three metal $[M(bpaqH)Cl]^{2+}$ complexes create a distorted-octahedral coordination environment to give an electronic structure involving three nonbonding t_{2g} -like molecular orbitals (MO) and two antibonding e_g -like MOs.

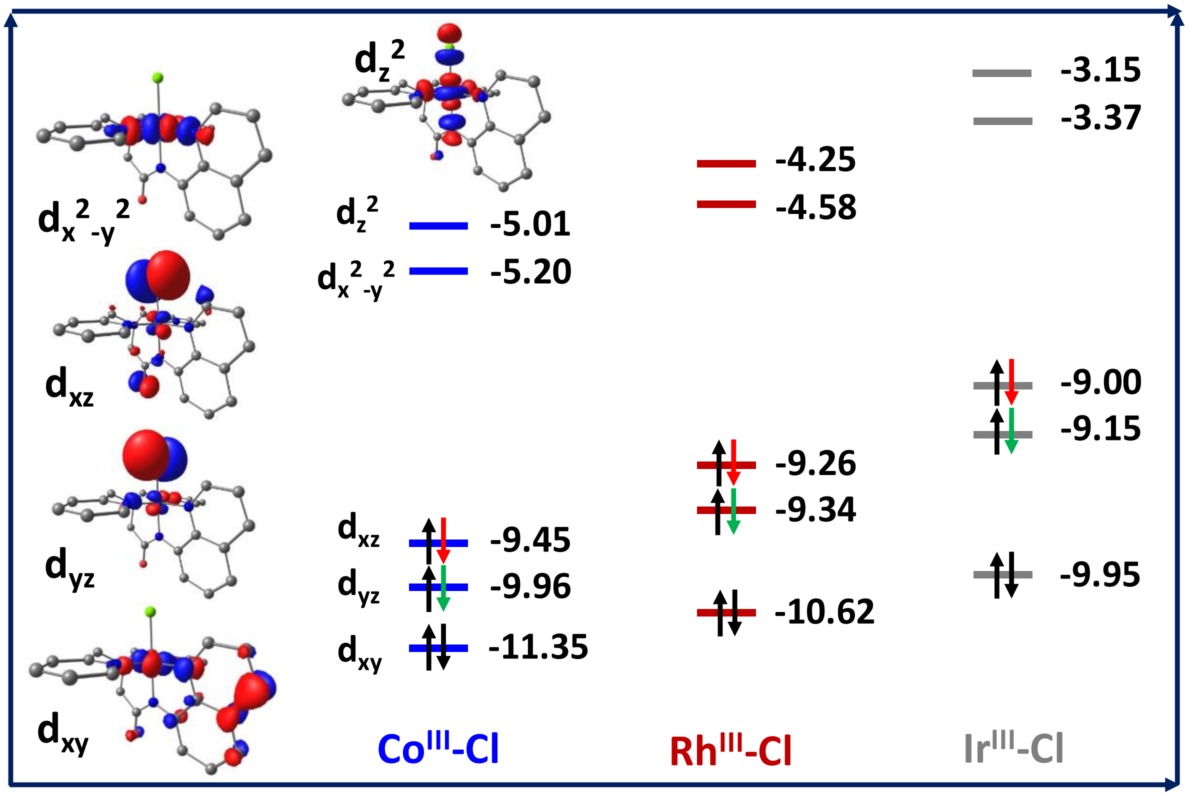

Figure S1. Conceptual MO-diagram of Electronic distribution in the singlet state of $[M(bpaqH)Cl]^{2+}$ (M=Co, Rh and Ir). H-atoms are omitted for clarity.

Besides, comparison of the frontier MOs of the Co^{III}–Cl, Rh^{III}–Cl, and Ir^{III}–H indeed shows that an electronic distribution in the singlet state of $[M(bpaqH)Cl]^{2+}$ (M=Co, Rh and Ir), which is easy to prepare and is an 18-electron Co(III)–d^6 complex adopting a low spin (LS) state (**Figure S1**). Upon comparing the pairing energy, there is an increase in going from Co to Ir, while the pairing energy differences (between t_{2g} and e_g) was estimated to be in the order of 4.25 eV for Co < 4.68 eV for Rh < 5.63 eV for Ir. This allows us to envision that reduction leads to the occupation of the LUMO and lowering the d_{z2}-based σ*–MO to afford a 19-electron LS–Co(II)–d^7 species. To remain as a more stable structure, the reduction of d^6 to d^7 of 2-(bis(pyridin-2-ylmethyl)amino)-N-(quinolin-8-yl)acetamide based Co, Rh and Ir complexes takes place. Thus, pairing energy helps

to stabilize the reduced state and M(II)–d^7 species renders the complex less reactive, as the electronic stress is delocalized over all the Co-N_{1-5} bonds.

Section S1.2: Analyses of the Three Redox States with Possible Catalytic Pathways

Our earlier theoretical study on ($[Co(PY5Me_2)(CH_3CN)]^{2+}$) was devoted to the detailed description of the geometrical and electronic structural characteristics of the systems.[2] With regard to the computational methodology used, the B3LYP functional and the Ahlrich's group all-electron triple-z basis set (TZVP) were utilized for all the atoms in single point calculations at solvent medium by using Gaussian at B3LYP/TZVP [in bracket] and ORCA at B3LYP/def2-SVP basis sets (**Table S2**). In the previous study,[2] we have considered and compared the stability of all spin configurations with their relative free energies and high spin for $[Co]^{2+}$ and low spin for $[Co]^{3+}$ configurations were considered in entire catalytic cycles, which were more stable intermediates with the other ligand skeletons.[2,9] Hence, in our present study as well, we have considered the configurations of high spin $[M]^{2+}$ and low spin $[M]^{3+}$ redox species. In the proposed reaction pathway, initial reduction followed by protonation and then reduction leads to generation of molecular hydrogen. So that, it is vital to determine the reduction potential and pK_a values for each step. It is also known that metal hydride complexes, M−H, can be formed through an electron-transfer proton-transfer (ETPT) reaction in the presence of acid, where upon one-electron reduction of M(II) in M-bpaqH can form a M(I) center, and the latter can be protonated to produce a terminal M^{III}−H hydride species.

Table S2 (A). Redox properties compared with available experimental results.

**Redox Properties**	**Co**		**Rh**	**Ir**
	**DFT**	**Exp.**	**DFT**	**DFT**
**$M^{III/II}$-Cl**	-0.324 [-0.059]	-0.33	-3.236 [-2.958]	-3.658 [-3.666]
**M^{II}-Cl/M^{I}**	-1.755 [-1.854]	-1.55	-0.481 [-0.626]	-0.549 [-0.539]
**M^{III}-H/M^{II}-H**	-1.288 [-1.954]	-	-4.522 [-4.710]	-4.020 [-3.781]
**pK_a**	6.86 [16.63]	-	34.88 [41.07]	45.28 [52.54]

Table S2 (B). Redox properties for Rh and Ir complexes with available experimental data with some other systems (See the reference, 10, 11, 12 and 13).

**Redox Properties**	**Rh**	**Ir**
	**DFT & (Experimental Available Data)**	**DFT & (Experimental Available Data)**
**$M^{III/II}$**	-3.236 & (-1.57[10],-1.26[11],-2.61[12])	-3.658 (-2.04[10], -2.10[11], 2.82[12])
**M^{II}/M^{I}**	-0.481 & (-0.80[11],-0.40,[13] -0.36[13])	-0.549 & (-1.70[11], -0.92, [13] -0.95[13])

The electrochemical reaction at the first case, $Co^{III/II}$–Cl first undergoes a one-electron reduction process that results are found to be -0.324 V (CPCM model), -0.308 V (CPCMC model) for cobalt, forming Co^{II}–Cl intermediate, which were good fit of the experimental peak of the first reduction (-0.33 V vs SCE). In that case, the formation of Co(II) that is further reduced by the one-electron ($1e^-$) to form a Co(I) species by -1.755 V which is consistent with experimental value of -1.55 V vs SCE for cobalt species. Furthermore, this particular step was protonated by directly with the appropriate pK_a value of 6.86, which leads to the formation of Co^{III}–H species. The computed pK_a values for the protonation of Rh(I) and Ir(I) species leading to the formation of M(III)–H species is found to be 34.88 and 45.28. Overall, Co(III) is characterized by a $3d^6$ configuration where unpaired electrons reside in the higher-energy eg orbitals due to crystal field splitting in octahedral complexes. This higher energy state makes Co(III) relatively unstable, leading to increased susceptibility to reduction to Co(II). Conversely, Ir(III) and Rh(III) exhibit configurations of $5d^6$ and $4d^6$, respectively. In these cases, the 5d and 4d orbitals experience a significantly stronger crystal field splitting compared to the 3d orbitals. This heightened splitting raises the energies of the e_g orbitals, as illustrated in Figure S1 (See the supporting information), making them energetically inaccessible for electrons. In essence, the significant difference in MIII/MII reduction potentials between Cobalt and Ir/Rh stems from a combination of factors, such as electronic configuration, d-orbital splitting, spin state, and other pertinent variables, working together.

Table S3. Comparison between different spin state energies (in kcal/mol) for all possible cobalt Co complexes (Here, more stable species are considering as a 0.0 kcal/mol).

**Species**	HS	IS	LS
Co^{II}	0.00	-	6.90
Co^{II}-Cl	0.00	-	3.87
Co^{II}-H	0.00	-	9.52
Co^{II}-OAc	0.00	-	3.97
Co^{III}-OAC	17.04	14.25	0.00
Co^{III}-H	65.46	14.49	0.00
Co^{III}-Cl	19.50	14.87	0.00

Table S4. Computed optimized geometries and their bond lengths of Co, Rh and Ir complexes.

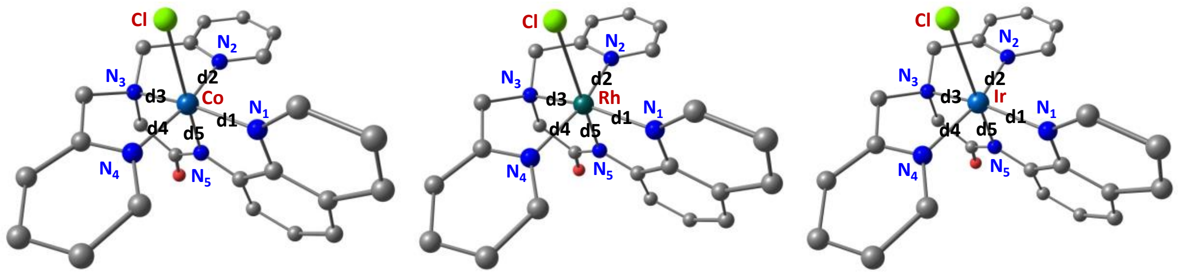

Species	d1 (Å)	d2 (Å)	d3 (Å)	d4 (Å)	d5 (Å)
**Co^{III}-Cl**	1.965	1.976	1.996	1.976	1.901
**Rh^{III}-Cl**	2.064	2.069	2.076	2.069	1.998
**Ir^{III}-Cl**	2.075	2.075	2.093	2.075	2.017
**Co^{II}-Cl**	2.129	2.250	2.265	2.250	2.064
**Rh^{II}-Cl**	2.046	2.458	2.178	2.458	1.973
**Ir^{II}-Cl**	2.045	2.533	2.181	2.533	1.969
**Co^{II}**	2.102	2.098	2.250	2.112	1.970
**Rh^{II}**	2.244	2.241	2.375	2.253	2.103
**Ir^{II}**	2.200	2.223	2.431	2.283	2.143
**Co^{I}**	2.071	2.078	2.291	2.025	2.029
**Rh^{I}**	2.264	2.193	2.394	2.198	2.173
**Ir^{I}**	2.073	2.066	2.111	2.058	2.131
**Co^{III}-H**	1.954	1.970	1.993	1.970	1.948
**Rh^{III}-H**	2.071	2.069	2.080	2.069	2.077
**Ir^{III}-H**	2.083	2.075	2.098	2.075	2.096
**Co^{II}-H**	2.147	2.238	2.307	2.309	2.121
**Rh^{II}-H**	2.064	2.380	2.255	2.406	2.061
**Ir^{II}-H**	2.066	2.065	2.112	2.066	2.094
**Co^{III}-OAc**	1.974	1.967	2.003	1.988	1.904
**Rh^{III}-OAc**	2.085	2.060	2.077	2.073	1.999
**Ir^{III}-OAc**	2.101	2.070	2.092	2.078	2.018
**Co^{II}-OAc**	2.151	2.214	2.276	2.245	2.086
**Rh^{II}-OAc**	2.303	2.408	2.388	2.376	2.293
**Ir^{II}-OAc**	2.060	2.550	2.188	2.442	1.972

Table S5. Computed spin density plot and natural atomic charges on selected species of Co, Rh and Ir complexes.

Species	Co		Rh		Ir	
	Spin Density	Atomic Charges	Spin Density	Atomic Charges	Spin Density	Atomic Charges
M^{I}	2.024	0.336	1.665	0.222	0.760	0.620
M^{II}	2.613	0.693	0.809	0.592	0.854	0.697
M^{II}-H	1.828	0.364	1.071	0.199	0.123	0.596
M^{II}-H_N1H	2.621	0.429	1.221	0.303	1.419	0.401
M^{II}-H_N2H	2.653	0.380	2.303	0.191	2.456	-0.031
M^{II}-OAc	2.684	0.560	1.019	0.623	0.185	0.983
M^{II}-Cl	2.663	0.439	1.026	0.464	0.198	0.719

Table S6. Computed stretching frequencies of all the transition states for three metal complexes.

Metal Hydride	${}_{HEP}M^{TS}$		${}_{RPP}M^{TS}$		${}_{LCP}M^{TS1}$	
	Frequency cm^{-1}	Force Constant mdyn $Å^{-1}$	Frequency cm^{-1}	Force Constant mdyn $Å^{-1}$	Frequency cm^{-1}	Force Constant mdyn $Å^{-1}$
Co	-484.8	0.15	-743.6	0.37	-943.0	0.54
Rh	-567.5	0.20	-848.0	0.49	-1009.9	0.62
Ir	-609.9	0.23	-932.7	0.72	-1085.4	0.82

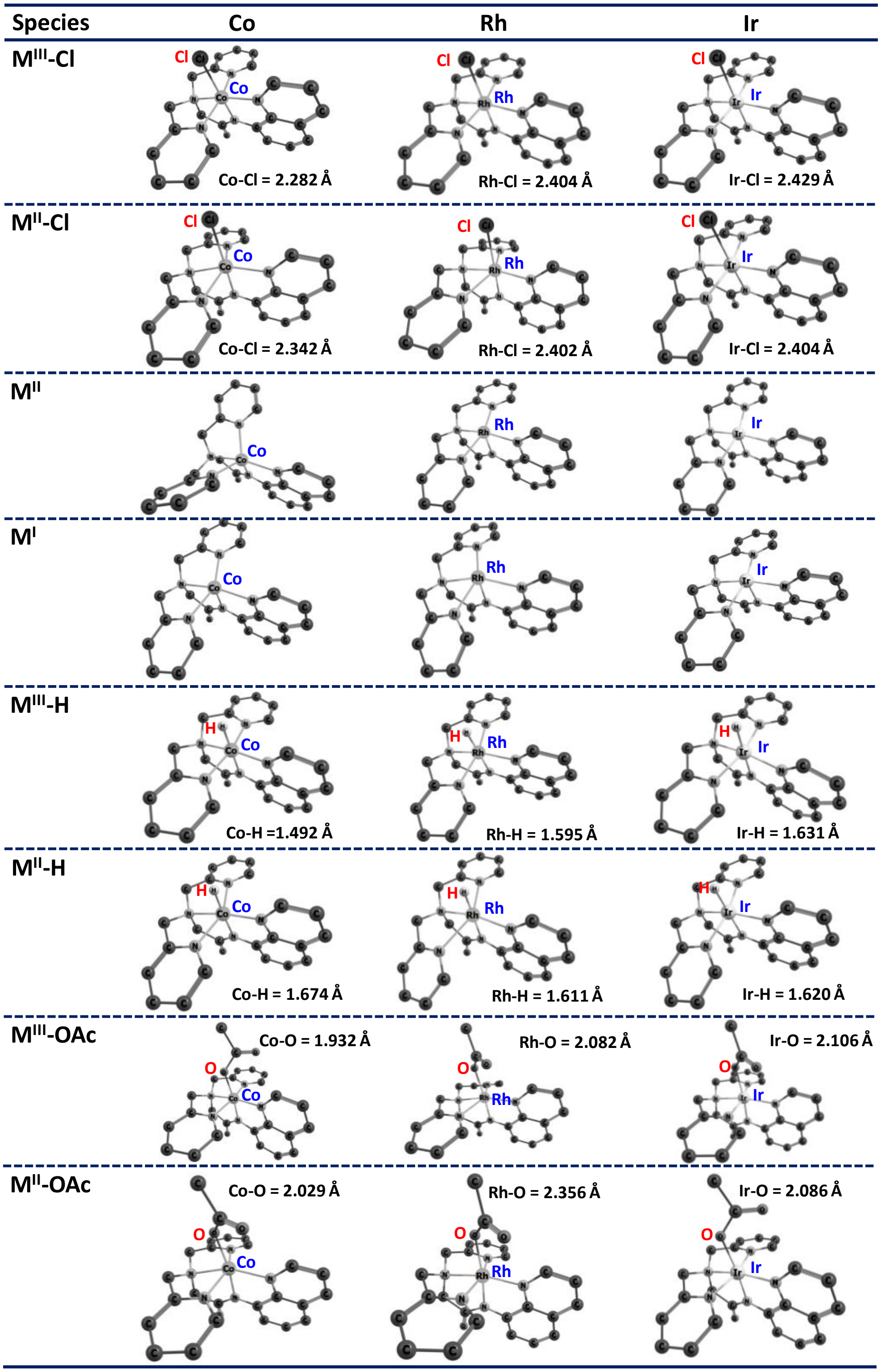

Figure S2. Optimized geometries of key intermediates involving both the schemes 1A for metal hydride formation) and Scheme 1B for hydrogen production of the selected species using three different (Co, Rh and Ir) metal systems. For clarity, hydrogen atoms are omitted.

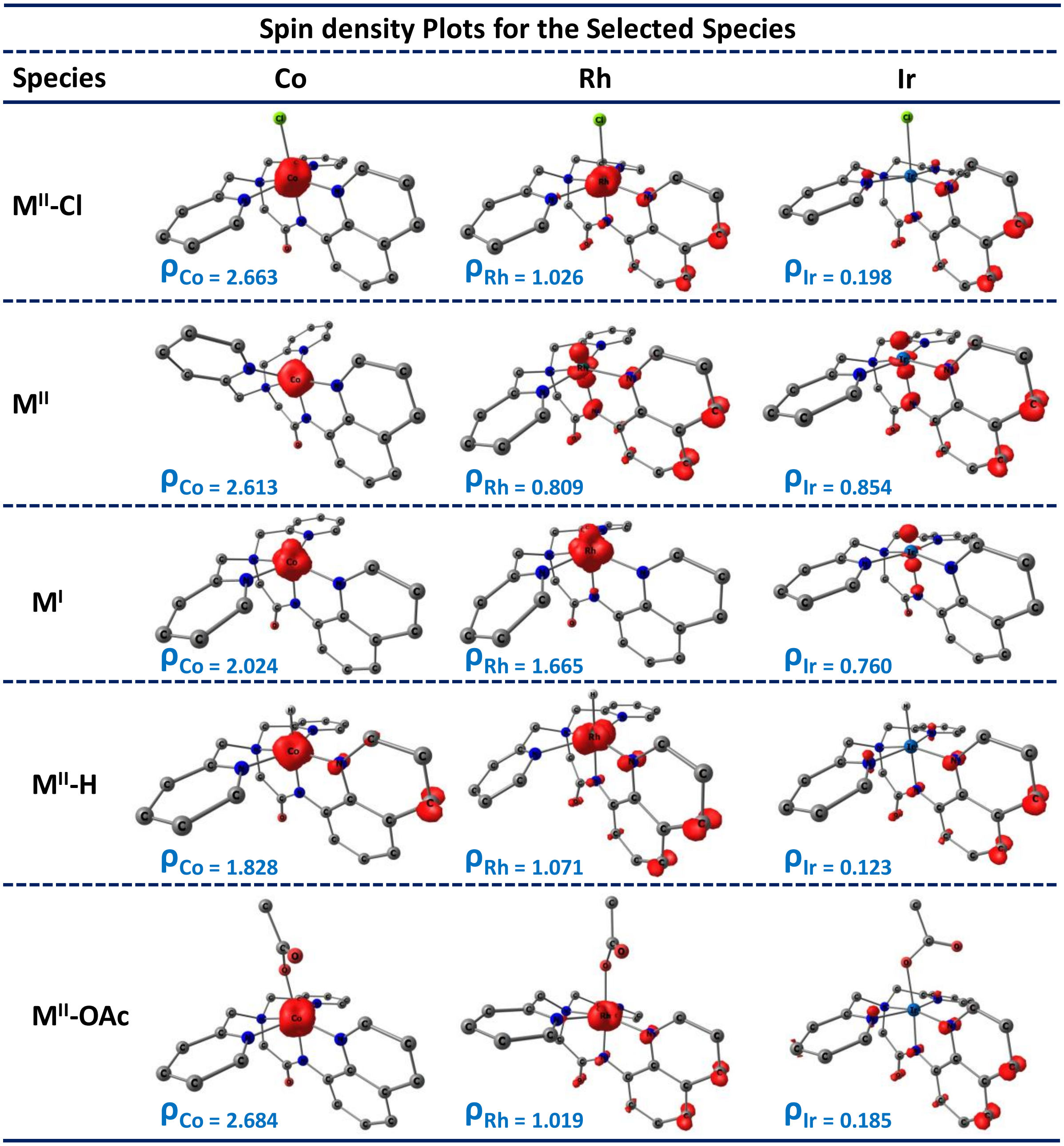

Figure S3. Spin density plots for selected optimized geometries of key intermediates shown in scheme 1 using three different (Co, Rh and Ir) metal systems. For clarity, hydrogen atoms are omitted.

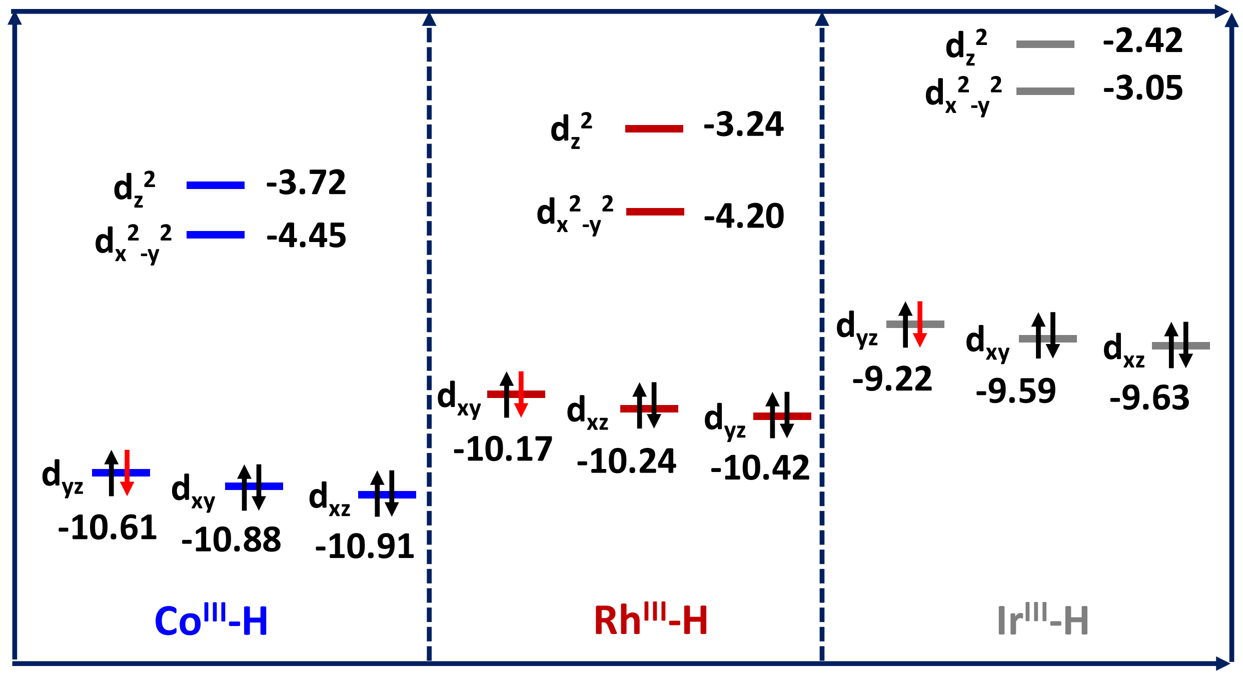

Figure S4. Electronic distribution of the frontier MOs in the singlet state of $[M(bpaqH)H]^{2+}$ metal hydride species.

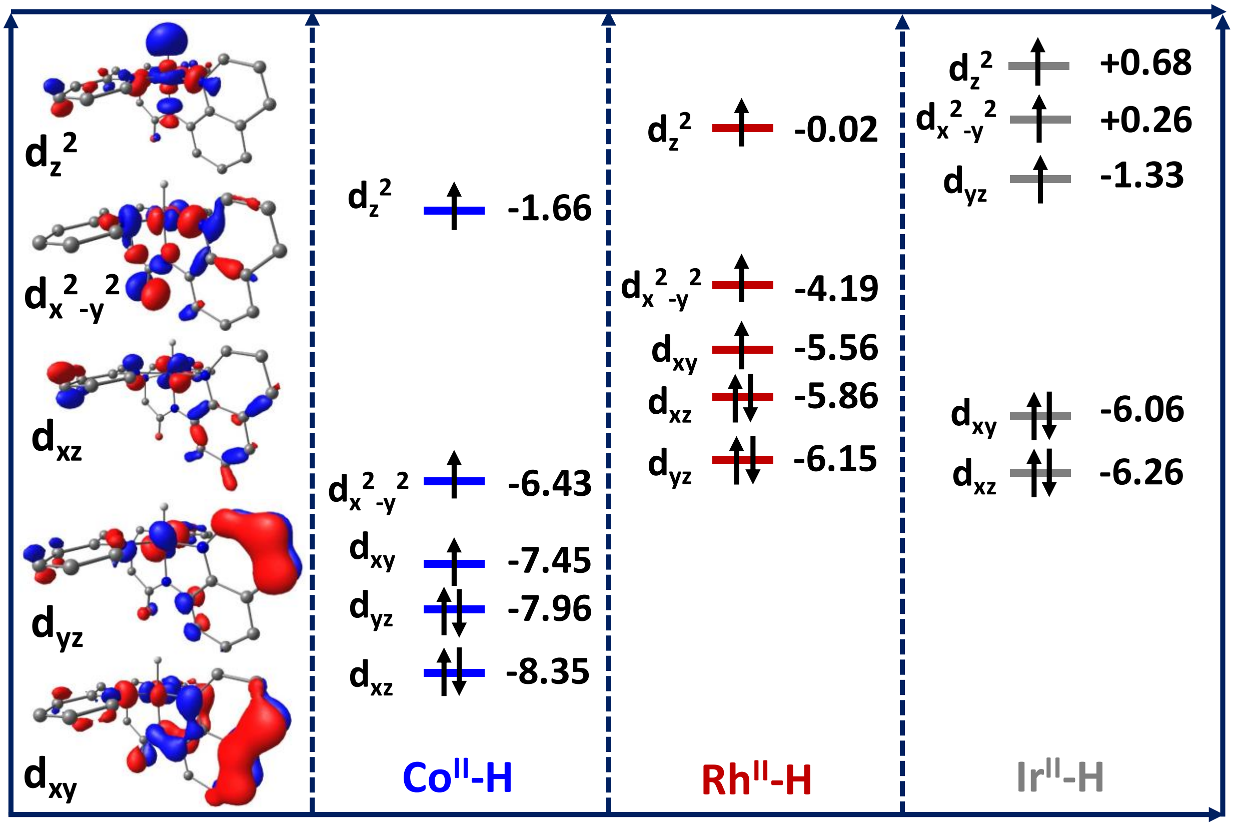

Figure S5. Electronic distribution of the frontier MOs in the quartet state of $[M(bpaqH)H]^{1+}$. H-atoms are omitted for clarity.

References

1. K. S. Karumban, A. Muley, B. Giri, S. Kumbhakar, T. Kella, D. Shee and S. Maji, Synthesis, characterization, structural, redox and electrocatalytic proton reduction properties of cobalt polypyridyl complexes. *Inorg. Chim. Acta*, **2022**, *529*, 120637–120645.
2. M. Panneerselvam and M. Jaccob, Role of Anation on the Mechanism of Proton Reduction Involving a Pentapyridine Cobalt Complex: A Theoretical Study. *Inorg. Chem*., **2018**, *57*, 8116–8127.
3. Yu, F.; Yu, L.; Mishra, I.K.; Yu, Y.; Ren, Z.F.; Zhou, H.Q. Recent developments in earth abundant and non-noble electrocatalysts for water electrolysis. *Mater. Today Phys.* **2018**, 7, 121–138.
4. Armstrong, J. E.; Crossland, P. M.; Frank, M. A.; VanDongen, M. J. McNamara, W. R. Hydrogen evolution catalyzed by a cobalt complex containing an asymmetric Schiff-base ligand. *Dalton Trans.*, **2016**, *45*, 5430–5433.
5. Rodenberg, A.; Orazietti, M.; Probst, B.; Bachmann, C.; Alberto, R.; Baldridge, K. K.; Hamm, P. 3d Element Complexes of Pentadentate Bipyridine-Pyridine-Based Ligand Scaffolds: Structures and Photocatalytic Activities. *Inorg. Chem.*, **2014**, *54*, 646–657.
6. a) Lewandowska, A. -A.; Baine, T.; Zhao, X.; Muckerman, J. T.; Fujita, E.; Polyansky, D. E. Mechanistic Studies of Hydrogen Evolution in Aqueous Solution Catalyzed by a Tertpyridine–Amine Cobalt Complex. *Inorg. Chem*., **2015**, *54,* 4310–4321. b) Kelly, C. P.; Cramer, C. J.; Truhlar, D. G. Single-Ion Solvation Free Energies and the Normal Hydrogen Electrode Potential in Methanol, Acetonitrile, and Dimethyl Sulfoxide. *J. Phys. Chem. B* **2007**, *111*, 408–422.
7. Singh, W. M.; Baine, T.; Kudo, S.; Tian, S.; Ma, X. A. N.; Zhou, H.; DeYonker, N. J.; Pham, T. C.; Bollinger, J. C.; Baker, D. L. Electrocatalytic and Photocatalytic Hydrogen Production in Aqueous Solution by a Molecular Cobalt Complex. *Angew. Chem. Int. Ed.*, **2012**, *51*, 5941–5944.
8. Nippe, M.; Khnayzer, R. S.; Panetier, J. A.; Zee, D. Z.; Olaiya, B. S.; Head-Gordon, M.; Chang, C. J.; Castellano, F. N.; Long, J. R. Catalytic protonreduction with transition metal complexes of the redox-active ligandbpy2PYMe. *Chem. Sci.*, **2013**, *4*, 3934–3945.
9. King, A. E.; Surendranath, Y.; Piro, N. A.; Bigi, J. P.; Longa, J. R.; Chang, C. J. A mechanistic study of protonreduction catalyzed by a pentapyridine cobalt complex: evidence for involvement of an anation-based pathway. *Chem. Sci*., **2013**, *4*, 1578–1587.
10. Kumar, A.; Comadoll, C. G.; King, D. S.; Oliver, A. G.; Day, V. W. Incorporation of [Cp*Rh] and [Cp*Ir] Species into Heterobimetallic Complexes via Protonolysis Reactivity and Dioximato Chelation and James D. Blakemore *Inorg. Chem.* **2021**, *60*, 14047−14059.
11. Holland, G. F.; Ellis, D. E.; Tyler, D. R.; Gray H. B.; Trogler. W. C. Theoretical, Spectroscopic, and Electrochemical Studies of Tetracobalt, Tetrarhodium, and Tetrairidium Dodecacarbonyl and Tris(dipheny1phosphino)methane-Substituted Derivatives *Am. Chem. SOC.* **1987**, *109*, 4276−4281.
12. Kaim, W.; Reinhardt, R.; Waldhor, E.; Fiedler. J. Electron transfer and chloride ligand dissociation in complexes [(CsMes)ClM(bpy)]÷/[(esMes)M(bpy)]" (M = Co, Rh, Ir; n = 2 +, +, O, -): A combined electrochemical and spectroscopic investigation. *Journal of Organometallic Chemistry* **1996**, *524*,195−202.

13. Bolinger, C. M.; Story, N.; Sullivan, B.P.; Meyer T. J. Electrocatalytic Reduction of Carbon Dioxide by 2,2'-Bipyridine Complexes of Rhodium and Iridium. *Inorg. Chem.* **1988**, *27*, 4582-4587

Cartesian coordinates of optimized structures of reactant, intermediates, transition states and products for all three metal (Co, Rh and Ir) complexes

Co^{III}-Cl

27 -0.491853000 0.000328000 -0.383728000
7 -0.680483000 1.966106000 -0.312814000
8 -0.139779000 -0.000834000 3.601494000
7 -2.242806000 0.001395000 0.575325000
7 0.321648000 -0.000735000 1.334943000
7 1.363563000 -0.000779000 -1.031470000
7 -0.683310000 -1.965187000 -0.313753000
6 -2.935469000 1.242671000 0.134398000
1 -3.334863000 1.058637000 -0.874626000
1 -3.768310000 1.501507000 0.808205000
6 2.259903000 -0.001545000 0.011141000
6 -0.477503000 -0.000346000 2.430725000
6 -1.930190000 2.357919000 0.022458000
6 4.527338000 -0.002900000 0.921783000
1 5.609552000 -0.003405000 0.774772000
6 0.272283000 2.886730000 -0.519489000
1 1.263865000 2.520536000 -0.785481000
6 1.708227000 -0.001551000 1.328515000
6 3.668934000 -0.002195000 -0.209707000
6 -1.967492000 0.000938000 2.056121000
1 -2.430905000 0.882383000 2.524163000
1 -2.432455000 -0.879815000 2.523933000
6 1.800117000 -0.000641000 -2.286231000
1 1.032163000 0.000014000 -3.063948000
6 2.582521000 -0.002249000 2.415658000
1 2.174142000 -0.002260000 3.424833000
6 0.268134000 -2.887062000 -0.520909000
1 1.260195000 -2.522165000 -0.786911000
6 -2.937216000 -1.238747000 0.133947000
1 -3.770429000 -1.496634000 0.807653000
1 -3.336325000 -1.053760000 -0.875018000
6 0.016788000 4.251704000 -0.403344000
1 0.819761000 4.968201000 -0.584884000
6 -2.258309000 -3.703059000 0.172461000
1 -3.273565000 -3.989300000 0.454773000
6 -1.933515000 -2.355367000 0.021559000
6 3.980460000 -0.002920000 2.192741000
1 4.643010000 -0.003455000 3.062006000
6 3.180309000 -0.001271000 -2.588011000
1 3.492849000 -0.001120000 -3.633749000
6 -1.272597000 -4.667604000 -0.048215000
1 -1.504644000 -5.729710000 0.059954000
6 -1.266047000 4.669258000 -0.046461000
1 -1.496637000 5.731652000 0.062010000
6 -2.253142000 3.706016000 0.173710000
1 -3.268052000 3.993582000 0.455922000
6 4.105600000 -0.002041000 -1.561665000
1 5.177311000 -0.002512000 -1.778886000
6 0.010748000 -4.251720000 -0.405210000
1 0.812687000 -4.969269000 -0.587159000
17 -1.448391000 0.001444000 -2.456486000

Co^{II}-Cl

27 -0.398665000 -0.000631000 -0.658801000
7 -0.814062000 2.187273000 -0.334846000
8 -0.109889000 0.000106000 3.503309000
7 -2.319553000 -0.003273000 0.543185000
7 0.422147000 0.000718000 1.235096000
7 1.682213000 0.002243000 -1.108975000
7 -0.807698000 -2.189489000 -0.334399000
6 -2.999222000 1.218523000 0.103977000
1 -3.317374000 1.052565000 -0.939655000
1 -3.895827000 1.449180000 0.713541000
6 2.464045000 0.003388000 0.012500000
6 -0.398159000 -0.000371000 2.307411000
6 -2.070371000 2.416208000 0.079127000
6 4.662368000 0.006402000 1.113693000
1 5.753519000 0.007840000 1.049875000
6 0.041763000 3.206190000 -0.450390000
1 1.052843000 2.953850000 -0.784341000
6 1.796715000 0.002604000 1.298770000
6 3.892607000 0.005273000 -0.079615000
6 -1.915663000 -0.002612000 1.968902000
1 -2.343273000 0.874068000 2.483837000
1 -2.340708000 -0.880497000 2.483912000
6 2.233894000 0.002865000 -2.312429000
1 1.536652000 0.001858000 -3.157641000
6 2.605640000 0.003797000 2.443735000
1 2.117526000 0.003239000 3.416508000
6 0.051181000 -3.205864000 -0.449673000
1 1.061513000 -2.950586000 -0.783655000
6 -2.995660000 -1.227096000 0.104093000
1 -3.891715000 -1.460220000 0.713537000
1 -3.314091000 -1.062232000 -0.939629000
6 -0.316880000 4.524530000 -0.164540000
1 0.412542000 5.329398000 -0.276786000
6 -2.495834000 -3.712785000 0.404810000
1 -3.518736000 -3.874386000 0.753302000
6 -2.063333000 -2.422072000 0.079580000
6 4.013934000 0.005663000 2.335861000
1 4.605062000 0.006543000 3.256713000
6 3.635695000 0.004688000 -2.496905000
1 4.048396000 0.005123000 -3.508170000
6 -1.604458000 -4.779736000 0.275435000
1 -1.920957000 -5.797044000 0.520585000
6 -1.618535000 4.775278000 0.274455000
1 -1.938056000 5.791686000 0.519417000
6 -2.506705000 3.705697000 0.404108000
1 -3.530067000 3.864297000 0.752630000
6 4.454824000 0.005878000 -1.385496000
1 5.543673000 0.007290000 -1.494374000
6 -0.303536000 -4.525204000 -0.163540000
1 0.428295000 -5.327915000 -0.275560000
17 -1.448982000 -0.002167000 -2.752657000

Co^{II}

27 -0.237292000 0.004901000 -0.195464000
7 -1.224191000 1.862502000 -0.386715000
8 0.903500000 -1.023785000 3.609257000
7 -1.817234000 -0.144112000 1.400575000
7 0.896736000 -0.398557000 1.364649000
7 1.624090000 0.153598000 -1.160986000
7 -1.415871000 -1.578941000 -0.907818000
6 -2.039743000 1.242638000 1.821264000

1	-2.968556000	1.364762000	2.409848000
1	-1.207579000	1.525235000	2.488511000
6	2.627723000	0.009928000	-0.233830000
6	0.343915000	-0.774830000	2.553573000
6	-2.029132000	2.196412000	0.644188000
6	5.012187000	-0.020525000	0.378091000
1	6.062012000	0.079145000	0.093567000
6	-1.102613000	2.702759000	-1.428363000
1	-0.426875000	2.393360000	-2.230152000
6	2.262538000	-0.285674000	1.130160000
6	4.004110000	0.138942000	-0.609642000
6	-1.186776000	-0.966497000	2.452506000
1	-1.631181000	-0.787602000	3.446992000
1	-1.355643000	-2.029022000	2.212791000
6	1.931362000	0.406545000	-2.429150000
1	1.097044000	0.501349000	-3.132906000
6	3.290935000	-0.438967000	2.061628000
1	3.027655000	-0.670986000	3.091768000
6	-1.016292000	-2.433996000	-1.865178000
1	-0.056057000	-2.217526000	-2.341834000
6	-2.986938000	-0.765546000	0.770802000
1	-3.676062000	-1.213077000	1.511084000
1	-3.560817000	0.019847000	0.248124000
6	-1.787051000	3.913676000	-1.499273000
1	-1.657774000	4.565640000	-2.365134000
6	-3.379090000	-2.905381000	-0.564478000
1	-4.310078000	-3.071423000	-0.017776000
6	-2.578924000	-1.801315000	-0.259690000
6	4.646120000	-0.303679000	1.678265000
1	5.417570000	-0.431379000	2.442075000
6	3.256733000	0.547071000	-2.883643000
1	3.450158000	0.754548000	-3.937668000
6	-2.971448000	-3.787039000	-1.567970000
1	-3.583904000	-4.656096000	-1.820760000
6	-2.631022000	4.262354000	-0.441900000
1	-3.187644000	5.202602000	-0.461028000
6	-2.754303000	3.391217000	0.642489000
1	-3.403991000	3.635083000	1.486070000
6	4.286563000	0.416008000	-1.970909000
1	5.327917000	0.519877000	-2.288307000
6	-1.766960000	-3.547900000	-2.233782000
1	-1.406058000	-4.216254000	-3.017680000

Co^{I}

27	-0.095630000	0.080498000	-0.187478000
7	0.110080000	1.925371000	-0.999097000
8	-0.101826000	0.056792000	3.981529000
7	-1.739881000	1.194426000	0.955312000
7	0.482637000	-0.209905000	1.736207000
7	1.651836000	-0.971148000	-0.548852000
7	-1.772287000	-1.079002000	-0.592849000
6	-1.277774000	2.584302000	0.872170000
1	-2.064505000	3.314126000	1.150048000
1	-0.463198000	2.688076000	1.609502000
6	2.339735000	-1.202959000	0.619505000
6	-0.312624000	0.141889000	2.773836000
6	-0.719785000	2.892333000	-0.495993000
6	4.296264000	-2.054295000	1.843996000
1	5.280931000	-2.528914000	1.839235000
6	0.702774000	2.162514000	-2.199557000
1	1.386107000	1.387101000	-2.558336000
6	1.721639000	-0.803458000	1.864494000
6	3.629293000	-1.829712000	0.613715000
6	-1.705224000	0.642249000	2.314491000
1	-2.078261000	1.359399000	3.069233000
1	-2.374729000	-0.232869000	2.355816000
6	2.194908000	-1.344231000	-1.711133000
1	1.605552000	-1.151228000	-2.613972000
6	2.422481000	-1.057634000	3.054491000
1	1.957406000	-0.767651000	3.994418000
6	-1.737692000	-2.320616000	-1.115059000
1	-0.741926000	-2.729923000	-1.309698000
6	-2.953060000	0.908518000	0.198953000
1	-3.881702000	1.123935000	0.765976000
1	-2.962832000	1.584318000	-0.678308000
6	0.499258000	3.324619000	-2.929072000
1	1.015800000	3.459001000	-3.882460000
6	-4.168857000	-1.213234000	-0.526754000
1	-5.120515000	-0.742095000	-0.267882000
6	-2.975575000	-0.519868000	-0.307223000
6	3.689490000	-1.671350000	3.029611000
1	4.203695000	-1.847040000	3.979308000
6	3.457379000	-1.959941000	-1.805413000
1	3.849592000	-2.237742000	-2.786581000
6	-4.131960000	-2.497294000	-1.074586000
1	-5.055891000	-3.052458000	-1.253068000
6	-0.358589000	4.315100000	-2.419768000
1	-0.542795000	5.240588000	-2.968973000
6	-0.972225000	4.076486000	-1.178897000
1	-1.642715000	4.817151000	-0.734768000
6	4.174646000	-2.199604000	-0.648621000
1	5.158978000	-2.674718000	-0.685683000
6	-2.883986000	-3.061361000	-1.378647000
1	-2.799479000	-4.066665000	-1.796437000

Co^{III}-H

27	-0.542210000	0.000600000	-0.624800000
7	-0.740237000	1.960727000	-0.612958000
8	-0.587274000	-0.000926000	3.420224000
7	-2.369145000	0.001823000	0.173687000
7	0.120809000	-0.000775000	1.207540000
7	1.359716000	-0.000707000	-1.076500000
7	-0.743430000	-1.959243000	-0.614448000
6	-3.016466000	1.238115000	-0.331551000
1	-3.343250000	1.048341000	-1.366793000
1	-3.908502000	1.505696000	0.259393000
6	2.158290000	-0.001702000	0.045720000
6	-0.779194000	-0.000311000	2.214467000
6	-2.008438000	2.356021000	-0.359430000
6	4.343042000	-0.003403000	1.148604000
1	5.433753000	-0.004037000	1.092831000
6	0.225502000	2.885521000	-0.726016000
1	1.234482000	2.522253000	-0.921647000
6	1.494566000	-0.001756000	1.318368000
6	3.583608000	-0.002521000	-0.050947000
6	-2.230036000	0.001310000	1.679463000
1	-2.740164000	0.882012000	2.098405000
1	-2.742089000	-0.878458000	2.098023000
6	1.912414000	-0.000569000	-2.284896000
1	1.230509000	0.000253000	-3.138036000
6	2.280245000	-0.002616000	2.473348000
1	1.786611000	-0.002651000	3.444428000
6	0.220816000	-2.885501000	-0.728213000
1	1.230318000	-2.523738000	-0.923960000

6	-3.018441000	-1.233175000	-0.332178000
1	-3.910852000	-1.499673000	0.258683000
1	-3.345024000	-1.042323000	-1.367288000
6	-0.034283000	4.249312000	-0.599216000
1	0.782875000	4.965412000	-0.702705000
6	-2.346451000	-3.697509000	-0.206098000
1	-3.378934000	-3.980464000	0.009331000
6	-2.012181000	-2.352655000	-0.360768000
6	3.690489000	-0.003431000	2.369788000
1	4.278911000	-0.004097000	3.291033000
6	3.313001000	-0.001398000	-2.465389000
1	3.719651000	-0.001247000	-3.478248000
6	-1.345890000	-4.663932000	-0.333178000
1	-1.582655000	-5.724283000	-0.218758000
6	-1.338545000	4.666247000	-0.330771000
1	-1.573691000	5.726926000	-0.216043000
6	-2.340655000	3.701343000	-0.204404000
1	-3.372775000	3.985832000	0.010753000
6	4.140170000	-0.002353000	-1.358123000
1	5.227234000	-0.002967000	-1.476826000
6	-0.041075000	-4.248939000	-0.601929000
1	0.774921000	-4.966277000	-0.706014000
1	-1.039915000	0.001653000	-2.032207000

Co^{II}-H

27	-0.432077000	-0.020216000	-1.014875000
7	-0.442765000	2.264818000	-0.676775000
8	-0.436109000	0.073767000	3.223404000
7	-2.396653000	0.401049000	0.120087000
7	0.241155000	-0.079186000	0.996169000
7	1.684552000	-0.313789000	-1.230961000
7	-1.273198000	-2.052597000	-0.599464000
6	-2.794520000	1.721438000	-0.364729000
1	-3.093107000	1.604522000	-1.420740000
1	-3.663840000	2.132894000	0.188353000
6	2.359268000	-0.393803000	-0.046009000
6	-0.635052000	0.088627000	2.007770000
6	-1.656425000	2.724957000	-0.338446000
6	4.438238000	-0.660551000	1.243218000
1	5.520707000	-0.808960000	1.274905000
6	0.589420000	3.109711000	-0.735859000
1	1.556553000	2.673462000	-1.005270000
6	1.589749000	-0.269831000	1.176157000
6	3.779016000	-0.586919000	-0.011607000
6	-2.101493000	0.343975000	1.567721000
1	-2.404956000	1.285113000	2.056899000
1	-2.707670000	-0.442079000	2.048854000
6	2.336169000	-0.413165000	-2.378448000
1	1.728130000	-0.335750000	-3.286135000
6	2.295294000	-0.349605000	2.387386000
1	1.731747000	-0.258202000	3.313658000
6	-0.616719000	-3.216272000	-0.611077000
1	0.448873000	-3.168014000	-0.856074000
6	-3.260468000	-0.677687000	-0.358485000
1	-4.229518000	-0.719755000	0.180264000
1	-3.473904000	-0.478672000	-1.423008000
6	0.463126000	4.474755000	-0.469704000
1	1.334192000	5.130641000	-0.532359000
6	-3.275130000	-3.202527000	0.026537000
1	-4.334682000	-3.154453000	0.289349000
6	-2.579214000	-2.029905000	-0.289441000
6	3.693343000	-0.542292000	2.403392000
1	4.198264000	-0.599051000	3.372711000
6	3.734894000	-0.607816000	-2.440624000
1	4.228908000	-0.686571000	-3.411722000
6	-2.595653000	-4.422315000	0.002513000
1	-3.118146000	-5.351430000	0.245007000
6	-0.794694000	4.964828000	-0.112158000
1	-0.936526000	6.024726000	0.115094000
6	-1.869380000	4.076249000	-0.038762000
1	-2.865430000	4.422304000	0.248293000
6	4.447288000	-0.693727000	-1.261411000
1	5.531176000	-0.843762000	-1.272690000
6	-1.237773000	-4.432984000	-0.323896000
1	-0.663954000	-5.361892000	-0.346420000
1	-1.092838000	0.123642000	-2.546524000

Co^{II}-OAc

27	-0.370159000	-0.126468000	-0.383387000
7	-1.561245000	1.530720000	0.552148000
8	1.114909000	-1.070474000	3.431607000
7	-1.711598000	-1.238400000	1.082280000
7	0.876617000	-0.181218000	1.288270000
7	1.310756000	1.040762000	-1.046842000
7	0.214323000	-2.237388000	-0.710397000
6	-2.914322000	-0.403698000	1.159246000
1	-3.456894000	-0.531799000	0.209225000
1	-3.589630000	-0.709820000	1.982679000
6	2.327595000	1.069239000	-0.129797000
6	0.488222000	-0.846117000	2.396318000
6	-2.592970000	1.076449000	1.281971000
6	4.587296000	1.746273000	0.574094000
1	5.529561000	2.258140000	0.362047000
6	-1.280813000	2.836066000	0.537908000
1	-0.435770000	3.140791000	-0.085447000
6	2.113034000	0.409951000	1.140328000
6	3.560538000	1.741802000	-0.407126000
6	-0.974244000	-1.356704000	2.359064000
1	-1.505889000	-0.798476000	3.147797000
1	-0.953579000	-2.405100000	2.702443000
6	1.444670000	1.663883000	-2.210648000
1	0.563735000	1.642298000	-2.862546000
6	3.158971000	0.444930000	2.072941000
1	3.007659000	-0.049372000	3.030151000
6	1.345583000	-2.686510000	-1.261258000
1	2.008242000	-1.930249000	-1.692801000
6	-1.930559000	-2.518165000	0.399651000
1	-2.435296000	-3.260892000	1.049100000
1	-2.576614000	-2.316247000	-0.469671000
6	-2.020656000	3.768945000	1.267702000
1	-1.760551000	4.828650000	1.226501000
6	-0.374745000	-4.477923000	-0.117907000
1	-1.080614000	-5.161889000	0.359050000
6	-0.642443000	-3.104829000	-0.145759000
6	4.372161000	1.105532000	1.779762000
1	5.160175000	1.107313000	2.539011000
6	2.630953000	2.348962000	-2.564152000
1	2.693825000	2.843979000	-3.535717000
6	0.801179000	-4.952802000	-0.703037000
1	1.030567000	-6.021579000	-0.693461000
6	-3.083656000	3.308411000	2.046329000
1	-3.679941000	4.003950000	2.642909000
6	-3.374055000	1.941964000	2.056121000
1	-4.198537000	1.547187000	2.654762000

6	3.680517000	2.381152000	-1.670383000
1	4.611155000	2.902159000	-1.915531000
6	1.681585000	-4.041345000	-1.288461000
1	2.615904000	-4.366147000	-1.750841000
6	-2.158068000	0.499366000	-2.680784000
8	-1.532271000	1.523722000	-2.955209000
8	-1.835631000	-0.359097000	-1.767531000
6	-3.430407000	0.147800000	-3.454021000
1	-3.645592000	0.910727000	-4.214105000
1	-3.313507000	-0.835784000	-3.937633000
1	-4.285386000	0.063840000	-2.762723000

Co^{III}-OAc

27	-0.393533000	0.193863000	-0.099057000
7	0.215886000	2.073285000	-0.320887000
8	0.919921000	0.410174000	3.680802000
7	-1.712636000	0.994890000	1.179006000
7	0.757104000	0.006390000	1.407294000
7	1.123666000	-0.600659000	-1.081449000
7	-1.289997000	-1.499827000	0.348014000
6	-1.969371000	2.364797000	0.661304000
1	-2.643411000	2.271452000	-0.201685000
1	-2.450096000	3.000018000	1.423157000
6	2.187614000	-0.849609000	-0.243770000
6	0.323137000	0.434726000	2.618136000
6	-0.677407000	2.961859000	0.167105000
6	4.461090000	-1.630735000	0.205475000
1	5.405256000	-2.053052000	-0.145558000
6	1.371733000	2.509621000	-0.841773000
1	2.061191000	1.752327000	-1.215214000
6	2.007266000	-0.527989000	1.135233000
6	3.412406000	-1.400652000	-0.724434000
6	-1.099152000	1.008057000	2.551931000
1	-1.065198000	2.037558000	2.939280000
1	-1.727190000	0.433639000	3.249452000
6	1.226355000	-0.876322000	-2.376895000
1	0.344180000	-0.708323000	-2.992174000
6	3.058885000	-0.768355000	2.020133000
1	2.930507000	-0.527573000	3.073890000
6	-0.846155000	-2.739917000	0.102895000
1	0.095960000	-2.829532000	-0.435729000
6	-2.908271000	0.115052000	1.110672000
1	-3.572200000	0.269966000	1.976818000
1	-3.466000000	0.378762000	0.200240000
6	1.687869000	3.865756000	-0.905506000
1	2.638648000	4.178922000	-1.340449000
6	-3.225071000	-2.402017000	1.431963000
1	-4.166412000	-2.228414000	1.957466000
6	-2.464305000	-1.320124000	0.990518000
6	4.271313000	-1.317577000	1.539307000
1	5.078862000	-1.496431000	2.253819000
6	2.407571000	-1.418461000	-2.930942000
1	2.437477000	-1.629031000	-4.001416000
6	-2.765153000	-3.697508000	1.184185000
1	-3.344000000	-4.562197000	1.517356000
6	0.776452000	4.793607000	-0.399589000
1	0.997188000	5.863398000	-0.425843000
6	-0.422435000	4.333247000	0.149503000
1	-1.157025000	5.028944000	0.560401000
6	3.490480000	-1.678835000	-2.115069000
1	4.410859000	-2.101747000	-2.527256000
6	-1.558298000	-3.867703000	0.505343000
1	-1.164276000	-4.860905000	0.283630000
6	-2.091407000	-0.279031000	-2.430877000
8	-1.715379000	-1.435474000	-2.563142000
8	-1.637667000	0.561562000	-1.530715000
6	-3.144068000	0.334044000	-3.344055000
1	-3.502580000	-0.416335000	-4.059843000
1	-3.992078000	0.722127000	-2.757402000
1	-2.712902000	1.189543000	-3.888018000

$_{HEP}Co^{TS}$

27	-0.201680000	0.300076000	-0.093867000
7	0.956624000	1.831237000	-0.705743000
8	1.798226000	0.847773000	3.275190000
7	-1.019309000	1.690350000	1.071243000
7	1.050312000	0.015749000	1.263160000
7	0.753154000	-1.102469000	-1.041735000
7	-1.471511000	-0.889970000	0.815676000
6	-0.905119000	2.969119000	0.320682000
1	-1.686092000	2.969954000	-0.457377000
1	-1.080477000	3.835649000	0.978983000
6	1.861597000	-1.502162000	-0.337371000
6	0.996541000	0.818509000	2.361071000
6	0.451729000	3.030569000	-0.331482000
6	3.884738000	-2.832246000	-0.052424000
1	4.589661000	-3.581328000	-0.419756000
6	2.135752000	1.773265000	-1.343418000
1	2.499300000	0.783524000	-1.622337000
6	2.055483000	-0.891156000	0.935642000
6	2.756417000	-2.485945000	-0.843624000
6	-0.243188000	1.718297000	2.359861000
1	0.073893000	2.744900000	2.596427000
1	-0.892095000	1.390135000	3.185677000
6	0.451956000	-1.661008000	-2.208705000
1	-0.498261000	-1.353528000	-2.657258000
6	3.172287000	-1.253293000	1.686181000
1	3.335935000	-0.794182000	2.659535000
6	-1.443148000	-2.230472000	0.837497000
1	-0.642781000	-2.719036000	0.284131000
6	-2.434239000	1.260882000	1.273455000
1	-2.901172000	1.809944000	2.106843000
1	-2.982916000	1.448399000	0.325498000
6	2.867766000	2.920412000	-1.644676000
1	3.820457000	2.830567000	-2.169542000
6	-3.435188000	-0.906782000	2.185219000
1	-4.213408000	-0.344058000	2.704602000
6	-2.447523000	-0.225336000	1.474067000
6	4.072731000	-2.219902000	1.173782000
1	4.940871000	-2.488655000	1.780831000
6	1.292221000	-2.646701000	-2.777069000
1	1.016272000	-3.081397000	-3.739450000
6	-3.411719000	-2.301961000	2.210895000
1	-4.175715000	-2.857557000	2.759883000
6	2.361325000	4.162821000	-1.259826000
1	2.914285000	5.080450000	-1.474266000
6	1.136752000	4.217271000	-0.589015000
1	0.712256000	5.170805000	-0.267999000
6	2.434244000	-3.047780000	-2.109797000
1	3.091847000	-3.807314000	-2.541711000
6	-2.397060000	-2.973484000	1.526054000
1	-2.340099000	-4.063179000	1.516299000
6	-3.279658000	-0.127156000	-1.973785000
8	-2.344913000	-0.935025000	-1.723437000

8	-3.305945000	1.069736000	-1.548701000
6	-4.415921000	-0.577762000	-2.882885000
1	-4.519492000	-1.671787000	-2.878982000
1	-5.358762000	-0.095639000	-2.586236000
1	-4.194142000	-0.254472000	-3.914347000
1	-0.775643000	1.190070000	-1.967284000
1	-1.545628000	1.229746000	-1.803922000

$_{RPP}Co^{TS}$

27	-0.372714000	0.069747000	-0.543121000
7	1.292176000	1.613994000	-1.304526000
8	1.024726000	1.664985000	3.152182000
7	-1.064042000	2.183515000	0.171071000
7	0.683115000	0.292389000	1.286417000
7	1.131204000	-1.504517000	-0.626607000
7	-2.247810000	-0.230713000	0.702019000
6	-0.545926000	3.137434000	-0.819006000
1	-1.121406000	2.994292000	-1.740883000
1	-0.684231000	4.181035000	-0.492181000
6	1.895298000	-1.603629000	0.502492000
6	0.495503000	1.375141000	2.070940000
6	0.914177000	2.894691000	-1.148977000
6	3.665911000	-2.681054000	1.825061000
1	4.427090000	-3.449885000	1.928108000
6	2.555400000	1.354165000	-1.659265000
1	2.810782000	0.303822000	-1.763162000
6	1.661934000	-0.646131000	1.555268000
6	2.900881000	-2.613057000	0.634240000
6	-0.519780000	2.406492000	1.534440000
1	-0.028723000	3.385545000	1.592093000
1	-1.344681000	2.442229000	2.254774000
6	1.324543000	-2.352139000	-1.626200000
1	0.665467000	-2.239926000	-2.479693000
6	2.447190000	-0.757515000	2.708230000
1	2.290434000	-0.045329000	3.505516000
6	-2.609683000	-1.379150000	1.288637000
1	-1.971129000	-2.231679000	1.085241000
6	-2.532220000	2.110114000	0.162424000
1	-2.998886000	3.005275000	0.605464000
1	-2.857791000	2.044283000	-0.882622000
6	3.504077000	2.352147000	-1.881345000
1	4.517082000	2.087642000	-2.167423000
6	-4.152859000	0.844491000	1.677142000
1	-4.735985000	1.750892000	1.810982000
6	-3.001365000	0.865032000	0.887461000
6	3.427631000	-1.764726000	2.827922000
1	4.010274000	-1.810415000	3.745167000
6	2.302620000	-3.367572000	-1.587793000
1	2.420608000	-4.032223000	-2.437902000
6	-4.529869000	-0.350809000	2.287480000
1	-5.419165000	-0.392887000	2.910217000
6	3.118466000	3.680174000	-1.712140000
1	3.828370000	4.488819000	-1.862016000
6	1.803830000	3.954991000	-1.337039000
1	1.467919000	4.977108000	-1.188267000
6	3.080530000	-3.496918000	-0.458620000
1	3.838515000	-4.274144000	-0.389598000
6	-3.742990000	-1.485547000	2.092750000
1	-3.995112000	-2.434663000	2.554595000
6	-2.459338000	-1.757911000	-2.293459000
8	-1.549531000	-1.930676000	-1.455812000
8	-2.740308000	-0.620236000	-2.836394000
6	-3.322648000	-2.925443000	-2.745016000
1	-3.034366000	-3.841038000	-2.224829000
1	-4.377297000	-2.702678000	-2.549871000
1	-3.218088000	-3.064595000	-3.826563000
1	-1.097846000	0.688425000	-2.084897000
1	-1.858034000	0.132044000	-2.430176000

$_{LCP}Co^{TS1}$

27	-0.592551000	-0.309315000	-0.772869000
7	0.971394000	2.352403000	-1.489729000
8	0.848805000	1.527059000	2.656877000
7	-1.612399000	1.728996000	0.043539000
7	0.500928000	0.080639000	0.859768000
7	0.690473000	-1.972418000	-0.865817000
7	-2.532373000	-0.901419000	-0.259808000
6	-1.209351000	2.991671000	-0.595113000
1	-1.617265000	3.000334000	-1.618406000
1	-1.640537000	3.862309000	-0.064243000
6	1.661434000	-1.873842000	0.096585000
6	0.196765000	1.094278000	1.714907000
6	0.286508000	3.199755000	-0.694408000
6	3.702554000	-2.703212000	1.193616000
1	4.509635000	-3.436537000	1.258632000
6	2.292022000	2.442594000	-1.691382000
1	2.737808000	1.685423000	-2.341543000
6	1.587350000	-0.774459000	1.030658000
6	2.720213000	-2.834324000	0.176106000
6	-1.211625000	1.676950000	1.462862000
1	-1.268846000	2.667857000	1.947920000
1	-1.912084000	1.019628000	2.003028000
6	0.734074000	-2.956977000	-1.753996000
1	-0.055446000	-2.973609000	-2.512833000
6	2.580148000	-0.690685000	2.010134000
1	2.536312000	0.127951000	2.725426000
6	-2.855210000	-2.199461000	-0.127055000
1	-2.078090000	-2.922885000	-0.387988000
6	-3.043700000	1.484638000	-0.174684000
1	-3.683466000	2.153074000	0.434535000
1	-3.266787000	1.712912000	-1.231934000
6	3.027626000	3.455889000	-1.085227000
1	4.102978000	3.528041000	-1.255975000
6	-4.703443000	-0.300198000	0.549686000
1	-5.413313000	0.483883000	0.822392000
6	-3.436848000	0.043244000	0.068283000
6	3.616886000	-1.651118000	2.082654000
1	4.367618000	-1.546718000	2.870543000
6	1.740370000	-3.944145000	-1.749154000
1	1.726934000	-4.735799000	-2.500634000
6	-5.045071000	-1.648629000	0.673912000
1	-6.031904000	-1.936491000	1.044832000
6	2.356647000	4.357845000	-0.254809000
1	2.906654000	5.158310000	0.245984000
6	0.977053000	4.228871000	-0.056877000
1	0.438373000	4.919919000	0.593649000
6	2.726150000	-3.879772000	-0.783421000
1	3.521335000	-4.629820000	-0.748454000
6	-4.103619000	-2.619608000	0.326279000
1	-4.323592000	-3.685264000	0.413200000
1	-0.339770000	0.477052000	-2.242243000
1	0.229559000	1.270588000	-2.163396000

$_{LCP}Co^{TS2}$

27	0.519030000	-0.084494000	-0.395236000
7	1.771133000	-1.805957000	-0.338197000
8	-1.116322000	0.036684000	3.329220000
7	1.843925000	0.348372000	1.304712000
7	-0.893824000	0.140485000	0.999893000
7	-2.463720000	-1.020343000	-1.138215000
7	1.503882000	1.666605000	-1.054724000
6	3.112123000	-0.369342000	1.092323000
1	3.732382000	0.228209000	0.411991000
1	3.680094000	-0.483837000	2.026845000
6	-3.054391000	-0.219010000	-0.198783000
6	-0.448139000	0.035762000	2.303238000
6	2.861935000	-1.720386000	0.451286000
6	-5.101326000	0.872831000	0.651779000
1	-6.176215000	1.018030000	0.599507000
6	1.510966000	-2.959064000	-0.979603000
1	0.619476000	-2.967173000	-1.598639000
6	-2.274417000	0.417539000	0.825849000
6	-4.476134000	-0.017165000	-0.253274000
6	1.070830000	-0.163128000	2.453677000
1	1.256586000	-1.239020000	2.539938000
1	1.382319000	0.296382000	3.399800000
6	-3.179377000	-1.657083000	-2.052643000
1	-2.616218000	-2.241472000	-2.777924000
6	-2.943638000	1.309079000	1.662428000
1	-2.384428000	1.816477000	2.436533000

Rh^{III}-Cl

45	-0.477652000	0.000360000	-0.480390000
7	-0.722991000	2.051839000	-0.363447000
8	-0.102069000	-0.000886000	3.582737000
7	-2.270859000	0.001413000	0.566686000
7	0.387857000	-0.000750000	1.321172000
7	1.493842000	-0.000815000	-1.094001000
7	-0.725902000	-2.050822000	-0.364371000
6	-2.966588000	1.250845000	0.140946000
1	-3.375000000	1.067232000	-0.865326000
1	-3.796126000	1.501094000	0.822515000
6	2.352162000	-0.001575000	-0.012975000
6	-0.426278000	-0.000386000	2.408605000
6	-1.977759000	2.389396000	0.027065000
6	4.605012000	-0.002985000	0.945974000
1	5.689555000	-0.003517000	0.816846000
6	0.197962000	3.006349000	-0.561904000
1	1.190142000	2.672317000	-0.869067000
6	1.778198000	-0.001566000	1.302284000
6	3.767341000	-0.002271000	-0.200752000
6	-1.930308000	0.000866000	2.042447000
1	-2.381862000	0.882316000	2.521861000
1	-2.383314000	-0.880000000	2.521563000
6	1.966090000	-0.000727000	-2.335111000
1	1.218258000	-0.000077000	-3.133384000
6	2.636997000	-0.002283000	2.401965000
1	2.209630000	-0.002281000	3.403530000
6	0.193717000	-3.006540000	-0.563186000
1	1.186356000	-2.673786000	-0.870261000
6	-2.968344000	-1.246866000	0.140425000
1	-3.798243000	-1.496206000	0.821885000
1	-3.376481000	-1.062256000	-0.865777000
6	-0.093171000	4.358295000	-0.388018000
1	0.682406000	5.105237000	-0.565820000

6	1.525848000	2.122628000	-2.320127000
1	0.991056000	1.523343000	-3.050756000
6	2.007334000	1.811390000	1.321285000
1	1.102607000	2.236909000	1.770750000
1	2.855671000	2.127785000	1.945260000
6	2.327503000	-4.078763000	-0.862382000
1	2.080402000	-4.990242000	-1.395614000
6	2.838234000	3.532532000	-0.372886000
1	3.351206000	4.066076000	0.421261000
6	2.143015000	2.358731000	-0.087266000
6	-4.330897000	1.540044000	1.575325000
1	-4.789281000	2.239946000	2.267458000
6	-4.579946000	-1.568342000	-2.120749000
1	-5.126701000	-2.122701000	-2.875419000
6	2.858594000	4.007071000	-1.684861000
1	3.392498000	4.920892000	-1.927568000
6	3.453244000	-3.997418000	-0.041963000
1	4.109856000	-4.853336000	0.081307000
6	3.722270000	-2.802911000	0.626370000
1	4.586069000	-2.709959000	1.277251000
6	-5.214216000	-0.728264000	-1.231851000
1	-6.291996000	-0.593727000	-1.271214000
6	2.189688000	3.291331000	-2.678493000
1	2.182695000	3.625902000	-3.710098000
1	-0.319556000	-0.547200000	-1.903715000
1	-1.146126000	-0.775909000	-1.515710000

6	-2.334906000	-3.719686000	0.232101000
1	-3.348182000	-3.968640000	0.554304000
6	-1.981120000	-2.386761000	0.026088000
6	4.037809000	-0.002984000	2.207322000
1	4.684156000	-0.003528000	3.088716000
6	3.354330000	-0.001402000	-2.596368000
1	3.699900000	-0.001293000	-3.631631000
6	-1.383789000	-4.720639000	0.016761000
1	-1.644522000	-5.770639000	0.169243000
6	-1.377185000	4.722446000	0.018547000
1	-1.636468000	5.772757000	0.171362000
6	-2.329705000	3.722745000	0.233496000
1	-3.342654000	3.972997000	0.555726000
6	4.244234000	-0.002163000	-1.540176000
1	5.322512000	-0.002673000	-1.721654000
6	-0.099290000	-4.358140000	-0.389748000
1	0.675240000	-5.106099000	-0.567839000
17	-1.505122000	0.001600000	-2.653916000

Rh^{II}-Cl

45	-0.255292000	-0.000485000	-0.662350000
7	-0.815936000	2.376242000	-0.374776000
8	-0.098910000	0.000733000	3.403904000
7	-2.184800000	-0.003194000	0.347948000
7	0.513547000	0.000905000	1.155263000
7	1.729892000	0.002240000	-1.160694000
7	-0.809202000	-2.378851000	-0.374461000
6	-2.923290000	1.214411000	-0.071058000
1	-3.171445000	1.076547000	-1.136014000
1	-3.863213000	1.322241000	0.501801000
6	2.532801000	0.003614000	-0.046235000
6	-0.371355000	-0.000094000	2.212147000
6	-2.085927000	2.471708000	0.045535000
6	4.705863000	0.006951000	1.063814000

1	5.798029000	0.008491000	1.007738000
6	-0.024082000	3.449289000	-0.350696000
1	1.003500000	3.300331000	-0.697933000
6	1.895701000	0.002969000	1.228874000
6	3.963887000	0.005622000	-0.148630000
6	-1.861023000	-0.002632000	1.810020000
1	-2.316525000	0.875572000	2.295597000
1	-2.313464000	-0.882443000	2.295549000
6	2.306769000	0.002780000	-2.412452000
1	1.602510000	0.001611000	-3.246273000
6	2.685992000	0.004391000	2.429405000
1	2.181283000	0.003910000	3.392610000
6	-0.014319000	-3.449652000	-0.350187000
1	1.012847000	-3.297830000	-0.697414000
6	-2.919839000	-1.222924000	-0.070923000
1	-3.859450000	-1.333399000	0.501943000
1	-3.168372000	-1.085880000	-1.135896000
6	-0.470748000	4.698096000	0.092143000
1	0.206193000	5.555048000	0.093902000
6	-2.601441000	-3.688147000	0.515642000
1	-3.636889000	-3.744020000	0.860428000
6	-2.078929000	-2.477840000	0.045834000
6	4.065061000	0.006342000	2.323082000
1	4.673623000	0.007443000	3.231457000
6	3.672623000	0.004658000	-2.577656000
1	4.085985000	0.005008000	-3.588903000
6	-1.776587000	-4.816860000	0.533644000
1	-2.160315000	-5.774425000	0.895324000
6	-1.790172000	4.811650000	0.532981000
1	-2.176593000	5.768183000	0.894525000
6	-2.611838000	3.680605000	0.515168000
1	-3.647438000	3.733622000	0.859949000
6	4.532058000	0.006125000	-1.446252000
1	5.617938000	0.007620000	-1.564760000
6	-0.457472000	-4.699644000	0.092831000
1	0.221887000	-5.554681000	0.094752000
17	-1.224083000	-0.001876000	-2.860415000

Rh^{II}

45	-0.211396000	0.066626000	-0.412673000
7	-1.322998000	2.026638000	-0.430777000
8	0.922677000	-0.985134000	3.508059000
7	-1.823017000	-0.112397000	1.323054000
7	0.979658000	-0.401761000	1.256376000
7	1.859874000	0.187171000	-1.269728000
7	-1.525307000	-1.654894000	-0.989882000
6	-2.049623000	1.264553000	1.776114000
1	-2.951434000	1.349553000	2.412015000
1	-1.192549000	1.548124000	2.410651000
6	2.796695000	-0.001371000	-0.283277000
6	0.382721000	-0.750239000	2.439550000
6	-2.120901000	2.257884000	0.633177000
6	5.148444000	-0.099633000	0.457702000
1	6.213298000	-0.023669000	0.226181000
6	-1.283820000	2.916762000	-1.436199000
1	-0.612575000	2.684799000	-2.267783000
6	2.357035000	-0.303996000	1.059367000
6	4.196254000	0.095444000	-0.578538000
6	-1.149179000	-0.940032000	2.340855000
1	-1.571705000	-0.780997000	3.348336000
1	-1.312131000	-2.000992000	2.090843000
6	2.249809000	0.453260000	-2.511529000
1	1.461026000	0.589678000	-3.258546000
6	3.335262000	-0.485969000	2.040847000
1	3.016172000	-0.716873000	3.054542000
6	-1.184863000	-2.568021000	-1.915567000
1	-0.260809000	-2.378831000	-2.468661000
6	-3.008219000	-0.741952000	0.731616000
1	-3.693423000	-1.143254000	1.502404000
1	-3.572058000	0.033617000	0.184191000
6	-2.046142000	4.083722000	-1.434005000
1	-1.980370000	4.780612000	-2.271684000
6	-3.456510000	-2.957665000	-0.440676000
1	-4.350834000	-3.087838000	0.172838000
6	-2.645506000	-1.834189000	-0.257151000
6	4.711392000	-0.382468000	1.734145000
1	5.436719000	-0.534719000	2.537455000
6	3.603153000	0.560255000	-2.885161000
1	3.864694000	0.779372000	-3.922016000
6	-3.109548000	-3.903404000	-1.408385000
1	-3.732422000	-4.787588000	-1.564579000
6	-2.882830000	4.328560000	-0.343121000
1	-3.499668000	5.229781000	-0.304875000
6	-2.921943000	3.401528000	0.700957000
1	-3.565531000	3.563111000	1.568681000
6	4.570526000	0.382955000	-1.915220000
1	5.632299000	0.459544000	-2.165375000
6	-1.951667000	-3.705286000	-2.163033000
1	-1.636960000	-4.420485000	-2.925275000

Rh^{I}

45	-0.460673000	0.187721000	-1.015480000
7	0.171511000	2.287313000	-1.172476000
8	-0.320467000	0.384297000	3.259994000
7	-2.169541000	1.331901000	0.211180000
7	0.229339000	-0.039039000	1.033291000
7	1.465687000	-0.982974000	-1.230613000
7	-1.943522000	-1.402802000	-0.726577000
6	-2.132279000	2.657316000	-0.405761000
1	-2.617815000	2.578284000	-1.394198000
1	-2.695393000	3.416895000	0.175346000
6	2.043415000	-1.239524000	-0.012625000
6	-0.508841000	0.464416000	2.044212000
6	-0.713908000	3.160927000	-0.632558000
6	3.852959000	-2.238265000	1.342549000
1	4.788415000	-2.801176000	1.398333000
6	1.419625000	2.713817000	-1.432341000
1	2.098495000	1.970789000	-1.859744000
6	1.395631000	-0.754213000	1.195854000
6	3.273218000	-1.978452000	0.074789000
6	-1.751708000	1.300990000	1.625902000
1	-1.533353000	2.327606000	1.964721000
1	-2.581260000	0.953435000	2.265987000
6	2.046569000	-1.417013000	-2.340436000
1	1.537979000	-1.181688000	-3.281478000
6	2.016764000	-1.047726000	2.422203000
1	1.534104000	-0.692258000	3.329872000
6	-1.694136000	-2.722446000	-0.785669000
1	-0.787223000	-3.013334000	-1.322826000
6	-3.342047000	0.512599000	-0.090808000
1	-4.192072000	0.723266000	0.590737000
1	-3.671287000	0.768190000	-1.113067000
6	1.848218000	4.015337000	-1.172950000
1	2.875468000	4.305128000	-1.404881000

6	-3.917625000	-1.881156000	0.557042000
1	-4.792256000	-1.502989000	1.092139000
6	-3.051867000	-0.981298000	-0.068541000
6	3.221425000	-1.774623000	2.481902000
1	3.662768000	-1.972716000	3.463546000
6	3.256977000	-2.146650000	-2.345162000
1	3.688042000	-2.476462000	-3.293399000
6	-3.655629000	-3.252892000	0.493111000
1	-4.321159000	-3.970208000	0.979594000
6	0.944095000	4.919222000	-0.611809000
1	1.242877000	5.945407000	-0.384000000
6	-0.353299000	4.477426000	-0.335875000
1	-1.090320000	5.150148000	0.109830000
6	3.863548000	-2.424108000	-1.139581000
1	4.800592000	-2.987357000	-1.097429000
6	-2.519781000	-3.680018000	-0.197419000
1	-2.265300000	-4.739168000	-0.277756000

Rh^{III}-H

45	-0.525493000	0.000631000	-0.749213000
7	-0.789562000	2.051510000	-0.677660000
8	-0.544300000	-0.000991000	3.405396000
7	-2.395498000	0.001839000	0.162671000
7	0.192722000	-0.000763000	1.200552000
7	1.509989000	-0.000787000	-1.133979000
7	-0.792853000	-2.049902000	-0.679040000
6	-3.056867000	1.249209000	-0.310851000
1	-3.409290000	1.065496000	-1.338677000
1	-3.932405000	1.505109000	0.309557000
6	2.258438000	-0.001720000	0.031509000
6	-0.718216000	-0.000367000	2.196735000
6	-2.064292000	2.389403000	-0.355106000
6	4.421454000	-0.003469000	1.194809000
1	5.513083000	-0.004128000	1.159903000
6	0.140315000	3.011222000	-0.802028000
1	1.149871000	2.681703000	-1.052383000
6	1.569278000	-0.001745000	1.298476000
6	3.688781000	-0.002582000	-0.020990000
6	-2.182921000	0.001191000	1.670233000
1	-2.681710000	0.881779000	2.102166000
1	-2.683514000	-0.878605000	2.101696000
6	2.114336000	-0.000726000	-2.318606000
1	1.464781000	0.000028000	-3.196609000
6	2.334862000	-0.002636000	2.467891000
1	1.817853000	-0.002658000	3.426968000
6	0.135510000	-3.011013000	-0.803900000
1	1.145573000	-2.682966000	-1.054147000
6	-3.058847000	-1.244185000	-0.311630000
1	-3.934788000	-1.499066000	0.308626000
1	-3.410981000	-1.059261000	-1.339339000
6	-0.159583000	4.360780000	-0.621361000
1	0.626936000	5.108493000	-0.737810000
6	-2.433487000	-3.714982000	-0.146234000
1	-3.463284000	-3.958645000	0.123405000
6	-2.068090000	-2.385933000	-0.356562000
6	3.745371000	-0.003485000	2.401755000
1	4.311936000	-0.004168000	3.336579000
6	3.519950000	-0.001566000	-2.444727000
1	3.966253000	-0.001469000	-3.440727000
6	-1.471671000	-4.719265000	-0.286911000
1	-1.739874000	-5.766578000	-0.129536000
6	-1.464211000	4.721767000	-0.284378000
1	-1.730784000	5.769430000	-0.126569000
6	-2.427613000	3.718933000	-0.144219000
1	-3.457049000	3.964091000	0.125442000
6	4.298053000	-0.002474000	-1.304598000
1	5.389212000	-0.003114000	-1.375879000
6	-0.166500000	-4.360181000	-0.623825000
1	0.618835000	-5.109078000	-0.740660000
1	-1.059579000	0.001654000	-2.252466000

Rh^{II}-H

45	-0.323521000	-0.095237000	-0.913835000
7	-0.454942000	2.307059000	-0.841652000
8	-0.399959000	0.617427000	3.175061000
7	-2.320624000	0.324432000	0.046153000
7	0.332994000	0.021617000	1.036779000
7	1.696364000	-0.415490000	-1.199094000
7	-1.400492000	-2.208972000	-0.716238000
6	-2.744213000	1.683544000	-0.345222000
1	-3.092214000	1.625717000	-1.389544000
1	-3.593208000	2.031984000	0.274361000
6	2.413871000	-0.317351000	-0.022644000
6	-0.590364000	0.331097000	1.997988000
6	-1.616829000	2.696683000	-0.295375000
6	4.503690000	-0.341992000	1.252694000
1	5.592158000	-0.447397000	1.279628000
6	0.575056000	3.155204000	-0.882074000
1	1.499012000	2.771967000	-1.326762000
6	1.700064000	-0.069279000	1.191029000
6	3.843943000	-0.458492000	0.001157000
6	-2.056175000	0.212899000	1.513068000
1	-2.653723000	0.949352000	2.077211000
1	-2.395372000	-0.780919000	1.848962000
6	2.382899000	-0.656502000	-2.375292000
1	1.762857000	-0.721611000	-3.270255000
6	2.413302000	0.042342000	2.433347000
1	1.844802000	0.238454000	3.340113000
6	-0.829538000	-3.416351000	-0.755589000
1	0.232186000	-3.438249000	-1.021242000
6	-3.279636000	-0.694389000	-0.422660000
1	-4.225459000	-0.665931000	0.153795000
1	-3.522035000	-0.454628000	-1.471171000
6	0.491049000	4.458615000	-0.382055000
1	1.356338000	5.122860000	-0.435434000
6	-3.474264000	-3.216148000	-0.078265000
1	-4.525060000	-3.095687000	0.197412000
6	-2.696456000	-2.094841000	-0.388294000
6	3.789518000	-0.095654000	2.443936000
1	4.334423000	-0.011283000	3.388337000
6	3.748573000	-0.802436000	-2.417912000
1	4.235489000	-0.993607000	-3.377426000
6	-2.885862000	-4.482707000	-0.128397000
1	-3.472339000	-5.374380000	0.107706000
6	-0.712191000	4.875200000	0.188269000
1	-0.815239000	5.882498000	0.599966000
6	-1.784300000	3.978463000	0.237464000
1	-2.738010000	4.267551000	0.685842000
6	4.513468000	-0.705013000	-1.224748000
1	5.600023000	-0.816323000	-1.239417000
6	-1.536831000	-4.587807000	-0.474181000
1	-1.034365000	-5.556365000	-0.521110000
1	-0.740688000	-0.112222000	-2.470322000

Rh^{II}-OAc

45	-0.542548000	0.102228000	-0.523677000
7	-1.929033000	1.648238000	0.632462000
8	1.205718000	-1.122194000	3.315657000
7	-1.723258000	-1.144832000	1.136791000
7	0.915414000	-0.109866000	1.233431000
7	1.445756000	1.098258000	-1.126088000
7	0.144393000	-2.179575000	-0.875093000
6	-3.020458000	-0.457990000	1.230713000
1	-3.556044000	-0.641722000	0.286626000
1	-3.635487000	-0.853076000	2.063356000
6	2.469451000	0.985761000	-0.230534000
6	0.534242000	-0.788874000	2.337451000
6	-2.876635000	1.047807000	1.373296000
6	4.819884000	1.363016000	0.411631000
1	5.812027000	1.757799000	0.177777000
6	-1.804845000	2.978142000	0.666471000
1	-1.015073000	3.403805000	0.039864000
6	2.208193000	0.328652000	1.032000000
6	3.772442000	1.504032000	-0.534864000
6	-0.969978000	-1.124902000	2.404108000
1	-1.409350000	-0.361226000	3.066208000
1	-1.064970000	-2.086118000	2.939418000
6	1.633463000	1.699319000	-2.291362000
1	0.758830000	1.754224000	-2.945436000
6	3.285193000	0.212314000	1.926135000
1	3.107718000	-0.285429000	2.876020000
6	1.260850000	-2.610017000	-1.467125000
1	1.772054000	-1.891870000	-2.115978000
6	-1.830235000	-2.486606000	0.540360000
1	-2.212214000	-3.220165000	1.277246000
1	-2.554360000	-2.416089000	-0.284956000
6	-2.626964000	3.791751000	1.449565000
1	-2.491096000	4.875314000	1.446495000
6	-0.081084000	-4.305370000	0.200002000
1	-0.638520000	-4.951342000	0.882320000
6	-0.528974000	-3.003246000	-0.051397000
6	4.561007000	0.724560000	1.609411000
1	5.363610000	0.608158000	2.344147000
6	2.884156000	2.239660000	-2.668838000
1	2.989636000	2.725060000	-3.641696000
6	1.082209000	-4.760809000	-0.425581000
1	1.450113000	-5.773262000	-0.239552000
6	-3.609973000	3.181262000	2.230310000
1	-4.269573000	3.778992000	2.865016000
6	-3.733232000	1.790453000	2.195860000
1	-4.486666000	1.279805000	2.800522000
6	3.944476000	2.139622000	-1.793479000
1	4.927435000	2.544667000	-2.052489000
6	1.769033000	-3.898307000	-1.279776000
1	2.685777000	-4.206325000	-1.787094000
6	-2.300976000	0.341465000	-2.604751000
8	-1.297080000	1.100209000	-2.520050000
8	-2.518969000	-0.553117000	-1.738434000
6	-3.272559000	0.499998000	-3.759610000
1	-2.959267000	1.306479000	-4.435717000
1	-3.340822000	-0.447692000	-4.316997000
1	-4.278747000	0.715442000	-3.365856000

Rh^{III}-OAc

45	-0.398345000	0.179664000	-0.140986000
7	0.197199000	2.155162000	-0.347338000
8	0.998414000	0.405126000	3.685110000
7	-1.719356000	1.001703000	1.235424000
7	0.840398000	-0.024883000	1.414688000
7	1.227698000	-0.675512000	-1.128485000
7	-1.379323000	-1.564398000	0.352275000
6	-1.970117000	2.387938000	0.745677000
1	-2.669467000	2.310245000	-0.099759000
1	-2.426550000	3.014637000	1.529240000
6	2.283013000	-0.882938000	-0.259645000
6	0.400649000	0.412345000	2.622818000
6	-0.692681000	3.006476000	0.220396000
6	4.570827000	-1.621047000	0.219199000
1	5.522030000	-2.034477000	-0.123399000
6	1.321628000	2.630273000	-0.903521000
1	1.999498000	1.894223000	-1.338429000
6	2.096038000	-0.545011000	1.120099000
6	3.522244000	-1.424884000	-0.717984000
6	-1.038908000	0.974399000	2.585309000
1	-1.002574000	1.993192000	2.999324000
1	-1.641019000	0.376401000	3.285642000
6	1.349663000	-0.995659000	-2.412688000
1	0.458893000	-0.868246000	-3.030021000
6	3.149104000	-0.757042000	2.010708000
1	3.008820000	-0.504383000	3.060296000
6	-0.998973000	-2.822314000	0.087641000
1	-0.080066000	-2.943151000	-0.485729000
6	-2.924312000	0.124852000	1.186510000
1	-3.571055000	0.280359000	2.065481000
1	-3.489914000	0.402676000	0.284578000
6	1.611790000	3.993328000	-0.928418000
1	2.534533000	4.341471000	-1.395833000
6	-3.305895000	-2.379828000	1.513755000
1	-4.217012000	-2.170427000	2.078017000
6	-2.520808000	-1.327483000	1.044603000
6	4.373160000	-1.291008000	1.547046000
1	5.180073000	-1.445090000	2.267906000
6	2.548128000	-1.529318000	-2.936932000
1	2.595246000	-1.773421000	-3.999646000
6	-2.915395000	-3.694054000	1.241986000
1	-3.519856000	-4.532451000	1.596160000
6	0.709418000	4.882709000	-0.343707000
1	0.910340000	5.956668000	-0.338784000
6	-0.455098000	4.380793000	0.243256000
1	-1.178824000	5.049782000	0.713385000
6	3.624296000	-1.739362000	-2.100050000
1	4.559738000	-2.153936000	-2.485838000
6	-1.746552000	-3.918217000	0.514292000
1	-1.408642000	-4.927780000	0.274702000
6	-2.128155000	-0.223119000	-2.575311000
8	-1.612058000	-1.322764000	-2.741116000
8	-1.815500000	0.601454000	-1.607538000
6	-3.193617000	0.314491000	-3.518466000
1	-3.496322000	-0.465948000	-4.227887000
1	-4.069752000	0.674508000	-2.957375000
1	-2.791495000	1.176530000	-4.075057000

$_{HEP}Rh^{TS}$

45	-0.232167000	0.324276000	-0.190080000
7	0.962124000	1.947455000	-0.780801000
8	1.831364000	0.766011000	3.260723000
7	-1.009793000	1.754477000	1.115249000
7	1.087553000	-0.013154000	1.223441000

7	0.822513000	-1.165283000	-1.150206000
7	-1.601834000	-0.872548000	0.778971000
6	-0.858328000	3.058233000	0.411067000
1	-1.661520000	3.126750000	-0.339893000
1	-0.978672000	3.904058000	1.107961000
6	1.906568000	-1.555385000	-0.393381000
6	1.036534000	0.769172000	2.340032000
6	0.481978000	3.120733000	-0.292289000
6	3.920230000	-2.898218000	-0.044684000
1	4.629507000	-3.651221000	-0.395217000
6	2.118990000	1.924708000	-1.460227000
1	2.454586000	0.951216000	-1.822155000
6	2.075967000	-0.945154000	0.887988000
6	2.819304000	-2.541717000	-0.868016000
6	-0.187453000	1.698435000	2.379549000
1	0.163337000	2.705796000	2.648639000
1	-0.824033000	1.356987000	3.209372000
6	0.575864000	-1.726853000	-2.328232000
1	-0.353222000	-1.416941000	-2.817114000
6	3.167268000	-1.321500000	1.669466000
1	3.304492000	-0.862830000	2.646752000
6	-1.658632000	-2.212338000	0.763887000
1	-0.922285000	-2.726931000	0.147580000
6	-2.428207000	1.342732000	1.348628000
1	-2.855807000	1.868813000	2.217345000
1	-3.007239000	1.566918000	0.433422000
6	2.856390000	3.083402000	-1.698265000
1	3.789574000	3.024533000	-2.261124000
6	-3.480505000	-0.797854000	2.256706000
1	-4.193270000	-0.204525000	2.832738000
6	-2.498065000	-0.156712000	1.501844000
6	4.076469000	-2.293073000	1.188244000
1	4.922304000	-2.569252000	1.822651000
6	1.439755000	-2.711813000	-2.860756000
1	1.205067000	-3.147567000	-3.833475000
6	-3.544325000	-2.192532000	2.249334000
1	-4.309710000	-2.711924000	2.830912000
6	2.379143000	4.296707000	-1.201104000
1	2.936218000	5.222537000	-1.363186000
6	1.179462000	4.313022000	-0.483919000
1	0.783166000	5.245181000	-0.075859000
6	2.550879000	-3.108764000	-2.144387000
1	3.228602000	-3.868459000	-2.543414000
6	-2.619050000	-2.911059000	1.489961000
1	-2.636344000	-4.001427000	1.451468000
6	-3.245956000	-0.309443000	-2.000290000
8	-2.190999000	-0.999822000	-1.930295000
8	-3.404847000	0.791150000	-1.393772000
6	-4.387079000	-0.830232000	-2.868307000
1	-4.809663000	-1.739986000	-2.410286000
1	-5.180000000	-0.078165000	-2.974959000
1	-4.004799000	-1.121309000	-3.859037000
1	-0.975407000	1.390267000	-1.983880000
1	-1.724166000	1.297889000	-1.744962000

$_{RPP}Rh^{TS}$

45	-0.112132000	0.064922000	0.340984000
7	0.673924000	-1.730459000	0.974898000
8	1.417492000	-0.321033000	-3.586246000
7	-1.121390000	-1.288755000	-1.120130000
7	1.291073000	0.205752000	-1.317449000
7	1.469121000	1.419773000	1.108754000
7	-1.258112000	1.483707000	-0.603074000
6	-1.265974000	-2.518095000	-0.311070000
1	-2.070356000	-2.337496000	0.441228000
1	-1.535184000	-3.396900000	-0.926075000
6	2.458858000	1.626441000	0.195380000
6	0.922518000	-0.437163000	-2.470753000
6	-0.010695000	-2.800136000	0.483742000
6	4.598583000	2.622757000	-0.496960000
1	5.469327000	3.240084000	-0.258361000
6	1.757152000	-1.918389000	1.746494000
1	2.258324000	-1.012426000	2.092928000
6	2.359768000	1.009925000	-1.112700000
6	3.606142000	2.438551000	0.506938000
6	-0.223401000	-1.455414000	-2.285866000
1	0.269039000	-2.440333000	-2.216488000
1	-0.798252000	-1.466331000	-3.227011000
6	1.579743000	1.989795000	2.359543000
1	0.753680000	1.783066000	3.045405000
6	3.405761000	1.247268000	-2.101025000
1	3.289105000	0.786496000	-3.079755000
6	-1.128928000	2.818545000	-0.507139000
1	-0.349482000	3.172965000	0.169839000
6	-2.367086000	-0.557708000	-1.433528000
1	-2.786346000	-0.879336000	-2.404615000
1	-3.151956000	-0.774646000	-0.656893000
6	2.214125000	-3.189345000	2.086443000
1	3.095006000	-3.296499000	2.721934000
6	-3.021956000	1.775416000	-2.190126000
1	-3.765628000	1.324494000	-2.849820000
6	-2.189169000	0.942113000	-1.433009000
6	4.484947000	2.031704000	-1.783916000
1	5.269736000	2.207043000	-2.524234000
6	2.649182000	2.768429000	2.730088000
1	2.686690000	3.195583000	3.734819000
6	-2.902111000	3.159401000	-2.083170000
1	-3.553178000	3.815657000	-2.666235000
6	1.526322000	-4.300310000	1.593657000
1	1.858194000	-5.313303000	1.834898000
6	0.410416000	-4.100294000	0.780451000
1	-0.146940000	-4.946752000	0.374249000
6	3.692669000	3.010914000	1.796359000
1	4.553681000	3.628256000	2.061579000
6	-1.936340000	3.694034000	-1.225981000
1	-1.801949000	4.771328000	-1.113908000
6	-4.208661000	-1.309335000	1.623050000
8	-4.579646000	-0.860509000	0.519546000
8	-3.010435000	-1.621268000	1.934887000
6	-5.257551000	-1.513576000	2.722444000
1	-5.209413000	-2.549807000	3.094592000
1	-5.022378000	-0.858896000	3.578117000
1	-6.268959000	-1.291273000	2.354776000
1	-1.208987000	0.188550000	1.871225000
1	-1.765624000	-0.398770000	1.726488000

$_{LCP}Rh^{TS1}$

45	-0.465945000	-0.453736000	-0.832146000
7	0.292169000	2.714294000	-1.500780000
8	0.538703000	1.686457000	2.658889000
7	-1.968397000	1.277633000	0.095988000
7	0.558390000	0.133262000	0.914561000
7	1.401166000	-1.737614000	-0.893952000
7	-2.332122000	-1.530077000	-0.235785000

6	-1.940795000	2.629500000	-0.511817000
1	-2.352102000	2.543576000	-1.522586000
1	-2.591599000	3.314160000	0.053922000
6	2.242592000	-1.414806000	0.140729000
6	0.001901000	1.079338000	1.736631000
6	-0.564334000	3.256122000	-0.613095000
6	4.371166000	-1.699978000	1.346617000
1	5.336915000	-2.188147000	1.438980000
6	1.513909000	3.223010000	-1.694652000
1	2.136695000	2.709203000	-2.421526000
6	1.828712000	-0.419137000	1.098276000
6	3.516779000	-2.050778000	0.269712000
6	-1.510091000	1.299753000	1.506779000
1	-1.789421000	2.236717000	2.005102000
1	-2.016766000	0.487241000	2.039572000
6	1.775553000	-2.643783000	-1.790426000
1	1.072375000	-2.846894000	-2.593931000
6	2.708755000	-0.107875000	2.135967000
1	2.414293000	0.639570000	2.858559000
6	-2.376565000	-2.868610000	-0.107026000
1	-1.502288000	-3.409139000	-0.455143000
6	-3.323096000	0.706704000	-0.048355000
1	-4.048458000	1.210289000	0.609866000
1	-3.646925000	0.890471000	-1.080728000
6	1.949256000	4.347863000	-0.999456000
1	2.941457000	4.747971000	-1.176663000
6	-4.495503000	-1.385383000	0.774239000
1	-5.316630000	-0.766648000	1.122405000
6	-3.376442000	-0.788701000	0.193000000
6	3.961896000	-0.747756000	2.251999000
1	4.609200000	-0.472535000	3.079595000
6	3.009544000	-3.316571000	-1.732727000
1	3.260386000	-4.049884000	-2.491660000
6	-4.546736000	-2.773977000	0.896830000
1	-5.412350000	-3.253169000	1.343856000
6	1.081923000	4.931225000	-0.076895000
1	1.388282000	5.804714000	0.490159000
6	-0.187874000	4.381071000	0.117461000
1	-0.878637000	4.819786000	0.829636000
6	3.873906000	-3.017231000	-0.701253000
1	4.837150000	-3.514381000	-0.621602000
6	-3.467727000	-3.532285000	0.444837000
1	-3.460650000	-4.613920000	0.524286000
1	-0.240573000	0.595893000	-2.334667000
1	-0.021245000	1.512830000	-2.006447000

Ir^{III}-Cl

77	-0.475080000	0.000354000	-0.484870000
7	-0.728266000	2.057231000	-0.372480000
8	-0.099425000	-0.000821000	3.596895000
7	-2.279241000	0.001411000	0.577288000
7	0.398361000	-0.000625000	1.333691000
7	1.508908000	-0.000816000	-1.092819000
7	-0.731150000	-2.056213000	-0.373287000
6	-2.975079000	1.255519000	0.155014000
1	-3.390440000	1.068655000	-0.848072000
1	-3.798092000	1.513269000	0.841136000
6	2.365194000	-0.001541000	-0.005580000
6	-0.417412000	-0.000312000	2.421145000
6	-1.983791000	2.394415000	0.028335000
6	4.617107000	-0.002989000	0.956583000
1	5.701634000	-0.003551000	0.827212000
6	0.186682000	3.018294000	-0.583965000
1	1.176697000	2.686042000	-0.898990000
6	1.790513000	-0.001477000	1.311328000
6	3.780712000	-0.002279000	-0.190606000
6	-1.925969000	0.000888000	2.055436000
1	-2.375254000	0.882214000	2.536649000
1	-2.376657000	-0.879865000	2.536386000
6	1.989218000	-0.000783000	-2.333044000
1	1.244492000	-0.000161000	-3.134002000
6	2.649357000	-0.002201000	2.410982000
1	2.220344000	-0.002165000	3.411989000
6	0.182449000	-3.018467000	-0.585169000
1	1.172939000	-2.687473000	-0.900034000
6	-2.976835000	-1.251559000	0.154530000
1	-3.800209000	-1.508403000	0.840558000
1	-3.391931000	-1.063718000	-0.848483000
6	-0.109493000	4.368928000	-0.414319000
1	0.661375000	5.117825000	-0.604097000
6	-2.341846000	-3.724052000	0.231118000
1	-3.352811000	-3.970828000	0.561853000
6	-1.987148000	-2.391793000	0.027390000
6	4.049748000	-0.002948000	2.218231000
1	4.695768000	-0.003496000	3.099793000
6	3.378140000	-0.001479000	-2.587980000
1	3.727919000	-0.001407000	-3.621837000
6	-1.397263000	-4.728453000	0.001434000
1	-1.660946000	-5.778035000	0.151009000
6	-1.390615000	4.730255000	0.003379000
1	-1.652817000	5.780145000	0.153407000
6	-2.336614000	3.727087000	0.232630000
1	-3.347229000	3.975143000	0.563477000
6	4.263412000	-0.002220000	-1.528377000
1	5.342495000	-0.002755000	-1.704754000
6	-0.115629000	-4.368756000	-0.416103000
1	0.654183000	-5.118656000	-0.606202000
17	-1.522584000	0.001483000	-2.676758000

Ir^{II}-Cl

77	-0.211383000	-0.000308000	-0.699658000
7	-0.843624000	2.436854000	-0.420043000
8	-0.102029000	0.000403000	3.364642000
7	-2.159484000	-0.003086000	0.281190000
7	0.536321000	0.000941000	1.122674000
7	1.776471000	0.002392000	-1.183670000
7	-0.836550000	-2.439380000	-0.419710000
6	-2.916853000	1.213417000	-0.131681000
1	-3.143896000	1.085796000	-1.202397000
1	-3.866982000	1.286981000	0.427771000
6	2.576232000	0.003609000	-0.056308000
6	-0.359163000	-0.000183000	2.170506000
6	-2.107510000	2.483599000	0.027730000
6	4.741197000	0.006665000	1.063809000
1	5.833668000	0.008118000	1.013320000
6	-0.068944000	3.520706000	-0.348146000
1	0.954590000	3.410570000	-0.720927000
6	1.933617000	0.002903000	1.211070000
6	4.006159000	0.005504000	-0.151410000
6	-1.847189000	-0.002476000	1.753002000
1	-2.307159000	0.876621000	2.231790000
1	-2.304457000	-0.882903000	2.231935000
6	2.363182000	0.002985000	-2.430940000
1	1.668303000	0.001935000	-3.272098000

6	2.706375000	0.004137000	2.407871000
1	2.198183000	0.003604000	3.369335000
6	-0.058743000	-3.520977000	-0.347631000
1	0.964443000	-3.407958000	-0.720503000
6	-2.913286000	-1.221879000	-0.131459000
1	-3.863198000	-1.298126000	0.427998000
1	-3.140690000	-1.095118000	-1.202200000
6	-0.531017000	4.733990000	0.171356000
1	0.130568000	5.602084000	0.210324000
6	-2.636571000	-3.662079000	0.570867000
1	-3.667117000	-3.680196000	0.933906000
6	-2.100259000	-2.489681000	0.028183000
6	4.093486000	0.005996000	2.310955000
1	4.692550000	0.006957000	3.225822000
6	3.732385000	0.004768000	-2.582791000
1	4.151656000	0.005163000	-3.591834000
6	-1.831194000	-4.804108000	0.637906000
1	-2.225937000	-5.733080000	1.057401000
6	-1.845172000	4.798923000	0.636962000
1	-2.242629000	5.726846000	1.056218000
6	-2.647247000	3.654560000	0.570109000
1	-3.677876000	3.669794000	0.933047000
6	4.582603000	0.006070000	-1.449578000
1	5.669201000	0.007483000	-1.561163000
6	-0.517269000	-4.735480000	0.172175000
1	0.146829000	-5.601649000	0.211292000
17	-1.165554000	-0.001629000	-2.907190000

Ir^{II}

77	-0.161175000	-0.054263000	-0.476859000
7	-1.322293000	1.911326000	-0.502739000
8	0.950529000	-1.021616000	3.519435000
7	-1.787404000	-0.155594000	1.328588000
7	1.019974000	-0.448149000	1.268519000
7	1.892301000	0.216278000	-1.220384000
7	-1.599197000	-1.670326000	-0.992801000
6	-1.951571000	1.237240000	1.759412000
1	-2.818391000	1.362586000	2.435661000
1	-1.055199000	1.509824000	2.342116000
6	2.834294000	0.034501000	-0.234448000
6	0.415721000	-0.795747000	2.445908000
6	-2.056399000	2.198154000	0.592721000
6	5.180992000	-0.003100000	0.515964000
1	6.244029000	0.114485000	0.294088000
6	-1.319649000	2.764913000	-1.540688000
1	-0.701721000	2.485728000	-2.398226000
6	2.395474000	-0.313885000	1.093623000
6	4.229849000	0.186590000	-0.521576000
6	-1.115010000	-0.987712000	2.343047000
1	-1.536473000	-0.827727000	3.350862000
1	-1.281945000	-2.047005000	2.088594000
6	2.277063000	0.530871000	-2.456362000
1	1.486350000	0.658611000	-3.200832000
6	3.372010000	-0.490685000	2.077931000
1	3.053255000	-0.755762000	3.083365000
6	-1.320020000	-2.572456000	-1.954398000
1	-0.403782000	-2.402775000	-2.524790000
6	-3.009934000	-0.752678000	0.784830000
1	-3.662821000	-1.158574000	1.580318000
1	-3.587567000	0.040703000	0.279384000
6	-2.054482000	3.949026000	-1.538218000
1	-2.019320000	4.614142000	-2.403198000
6	-3.569090000	-2.913932000	-0.435127000
1	-4.453177000	-3.024232000	0.196974000
6	-2.711035000	-1.828759000	-0.239596000
6	4.745176000	-0.333546000	1.782048000
1	5.470751000	-0.481890000	2.585863000
6	3.627026000	0.692178000	-2.816711000
1	3.883720000	0.947346000	-3.846471000
6	-3.284840000	-3.845341000	-1.435884000
1	-3.946037000	-4.699361000	-1.601199000
6	-2.824465000	4.252492000	-0.413360000
1	-3.417706000	5.169279000	-0.373878000
6	-2.827089000	3.362124000	0.662793000
1	-3.418950000	3.567467000	1.557758000
6	4.598143000	0.521605000	-1.848409000
1	5.657170000	0.640714000	-2.093050000
6	-2.137512000	-3.669676000	-2.212392000
1	-1.867806000	-4.373500000	-3.001969000

Ir^{I}

77	-0.546550000	0.153921000	-0.770570000
7	0.294688000	2.027260000	-0.916552000
8	-0.219699000	0.855510000	3.344810000
7	-2.121783000	1.205893000	0.162536000
7	0.200062000	0.005769000	1.220866000
7	1.195789000	-0.912070000	-1.129269000
7	-1.857763000	-1.423350000	-0.521985000
6	-2.014549000	2.598563000	-0.359425000
1	-2.478795000	2.581381000	-1.363333000
1	-2.579080000	3.313229000	0.265033000
6	1.952677000	-1.084219000	0.026695000
6	-0.504357000	0.651248000	2.167751000
6	-0.571937000	3.016327000	-0.493404000
6	3.951403000	-1.921809000	1.180075000
1	4.925244000	-2.416546000	1.140669000
6	1.577556000	2.374206000	-1.191678000
1	2.228466000	1.563966000	-1.524403000
6	1.431565000	-0.611433000	1.285664000
6	3.227358000	-1.736853000	-0.024632000
6	-1.897608000	1.125449000	1.660635000
1	-2.115168000	2.099139000	2.124952000
1	-2.627409000	0.405330000	2.064293000
6	1.676529000	-1.343131000	-2.310394000
1	1.027586000	-1.192374000	-3.175534000
6	2.184050000	-0.824235000	2.452515000
1	1.782274000	-0.454755000	3.395410000
6	-1.587031000	-2.754363000	-0.487436000
1	-0.571159000	-3.037176000	-0.769391000
6	-3.363264000	0.477613000	-0.256390000
1	-4.245556000	0.783313000	0.333901000
1	-3.536269000	0.764826000	-1.309666000
6	2.049745000	3.669506000	-1.052989000
1	3.093661000	3.884449000	-1.290171000
6	-4.098747000	-1.914213000	0.246316000
1	-5.082696000	-1.541217000	0.542414000
6	-3.125249000	-1.006398000	-0.155415000
6	3.426950000	-1.474705000	2.386006000
1	3.996629000	-1.619304000	3.308638000
6	2.928837000	-1.961057000	-2.430098000
1	3.267937000	-2.286020000	-3.416255000
6	-3.819919000	-3.286294000	0.261307000
1	-4.577774000	-4.008622000	0.570122000
6	1.181823000	4.678083000	-0.592230000

1	1.530306000	5.703463000	-0.454800000
6	-0.142682000	4.327286000	-0.311571000
1	-0.856302000	5.076505000	0.040571000
6	3.704676000	-2.165700000	-1.297693000
1	4.679589000	-2.655261000	-1.364734000
6	-2.528128000	-3.701487000	-0.120278000
1	-2.250989000	-4.757720000	-0.124214000

Ir^{III}-H

77	-0.525428000	0.000587000	-0.772212000
7	-0.793335000	2.057055000	-0.679745000
8	-0.539774000	-0.000686000	3.400342000
7	-2.405990000	0.001808000	0.158971000
7	0.199294000	-0.000660000	1.195070000
7	1.525026000	-0.000804000	-1.143961000
7	-0.796668000	-2.055522000	-0.680996000
6	-3.068360000	1.254515000	-0.307901000
1	-3.423886000	1.071660000	-1.334736000
1	-3.939118000	1.513284000	0.317496000
6	2.270388000	-0.001641000	0.027890000
6	-0.713348000	-0.000213000	2.192013000
6	-2.071508000	2.394328000	-0.353167000
6	4.428720000	-0.003326000	1.199194000
1	5.520453000	-0.003983000	1.166844000
6	0.134043000	3.023050000	-0.803316000
1	1.143357000	2.696006000	-1.056809000
6	1.578276000	-0.001593000	1.293574000
6	3.700403000	-0.002504000	-0.019030000
6	-2.181895000	0.001112000	1.669957000
1	-2.678060000	0.881583000	2.104303000
1	-2.679512000	-0.878831000	2.103714000
6	2.139096000	-0.000825000	-2.325734000
1	1.492859000	-0.000140000	-3.205660000
6	2.339633000	-0.002437000	2.465603000
1	1.818537000	-0.002411000	3.422477000
6	0.129169000	-3.022933000	-0.805028000
1	1.139031000	-2.697363000	-1.058244000
6	-3.070398000	-1.249504000	-0.308746000
1	-3.941600000	-1.507244000	0.316456000
1	-3.425584000	-1.065379000	-1.335472000
6	-0.169725000	4.370395000	-0.618419000
1	0.615383000	5.119680000	-0.734569000
6	-2.440636000	-3.718344000	-0.140278000
1	-3.470338000	-3.959068000	0.132177000
6	-2.075404000	-2.390912000	-0.354688000
6	3.749920000	-0.003288000	2.404676000
1	4.313690000	-0.003929000	3.341145000
6	3.545069000	-0.001656000	-2.442886000
1	3.996184000	-0.001625000	-3.436697000
6	-1.481687000	-4.725901000	-0.280288000
1	-1.751894000	-5.772094000	-0.119617000
6	-1.474025000	4.728317000	-0.277485000
1	-1.742534000	5.774854000	-0.116208000
6	-2.434582000	3.722225000	-0.137970000
1	-3.463876000	3.964448000	0.134698000
6	4.317375000	-0.002478000	-1.299329000
1	5.408878000	-0.003117000	-1.364106000
6	-0.176782000	-4.369891000	-0.620907000
1	0.607127000	-5.120375000	-0.737410000
1	-1.065834000	0.001534000	-2.311238000

Ir^{II}-H

77	-0.536586000	0.081475000	-0.798046000
7	-0.454153000	2.145596000	-0.757521000
8	-0.575494000	0.224693000	3.365572000
7	-2.405925000	0.421003000	0.125111000
7	0.176700000	-0.006693000	1.169503000
7	1.468631000	-0.276394000	-1.147626000
7	-1.164301000	-1.883648000	-0.705493000
6	-2.831451000	1.773880000	-0.365264000
1	-3.180185000	1.605811000	-1.401387000
1	-3.674628000	2.175090000	0.223565000
6	2.196415000	-0.384237000	0.019544000
6	-0.755344000	0.195420000	2.157199000
6	-1.657211000	2.709479000	-0.353133000
6	4.284743000	-0.734138000	1.244032000
1	5.360887000	-0.927927000	1.236000000
6	0.630598000	2.957980000	-0.878293000
1	1.551430000	2.473098000	-1.207557000
6	1.515271000	-0.237384000	1.276409000
6	3.608249000	-0.637109000	-0.004388000
6	-2.195215000	0.391144000	1.622357000
1	-2.573025000	1.326895000	2.061854000
1	-2.796042000	-0.427870000	2.047768000
6	2.119534000	-0.415855000	-2.362167000
1	1.484150000	-0.317706000	-3.242702000
6	2.252002000	-0.343252000	2.517213000
1	1.704409000	-0.224668000	3.450495000
6	-0.423181000	-3.021594000	-0.797884000
1	0.608178000	-2.889601000	-1.130139000
6	-3.271660000	-0.712172000	-0.346638000
1	-4.203835000	-0.785707000	0.240126000
1	-3.537008000	-0.455527000	-1.389103000
6	0.583265000	4.311091000	-0.601510000
1	1.486664000	4.911595000	-0.726433000
6	-3.035062000	-3.223165000	0.049730000
1	-4.076704000	-3.264316000	0.379973000
6	-2.489592000	-1.992797000	-0.302217000
6	3.606264000	-0.587780000	2.479016000
1	4.170741000	-0.671366000	3.411235000
6	3.467619000	-0.659886000	-2.443944000
1	3.932187000	-0.762712000	-3.427074000
6	-2.268228000	-4.390046000	-0.040574000
1	-2.689703000	-5.360731000	0.226365000
6	-0.627898000	4.891938000	-0.155073000
1	-0.689553000	5.954436000	0.086589000
6	-1.746281000	4.060407000	-0.032901000
1	-2.707193000	4.463180000	0.298820000
6	4.245746000	-0.776374000	-1.259520000
1	5.319284000	-0.969727000	-1.308011000
6	-0.930857000	-4.269237000	-0.489769000
1	-0.287582000	-5.145527000	-0.593299000
1	-1.040898000	0.148633000	-2.336736000

Ir^{II}-OAc

77	-0.317252000	0.054060000	-0.289695000
7	-1.837884000	1.688802000	0.701565000
8	1.192247000	-1.234278000	3.255069000
7	-1.682248000	-1.115442000	0.958989000
7	0.962185000	-0.159432000	1.195745000
7	1.289297000	1.070111000	-1.085237000
7	0.217369000	-2.376134000	-0.851475000
6	-2.989518000	-0.412677000	1.074005000
1	-3.482929000	-0.517922000	0.095463000

1	-3.628783000	-0.890520000	1.838332000
6	2.398241000	0.995008000	-0.261224000
6	0.531336000	-0.885334000	2.288465000
6	-2.831715000	1.068488000	1.355229000
6	4.736336000	1.437509000	0.281813000
1	5.705249000	1.867033000	0.012047000
6	-1.681126000	3.009976000	0.807011000
1	-0.862034000	3.445629000	0.227612000
6	2.252292000	0.330962000	0.984998000
6	3.658939000	1.567398000	-0.634971000
6	-0.979387000	-1.197225000	2.281104000
1	-1.441350000	-0.464530000	2.962835000
1	-1.119407000	-2.187811000	2.743837000
6	1.393834000	1.748960000	-2.283211000
1	0.467821000	1.846508000	-2.848591000
6	3.356379000	0.224386000	1.881873000
1	3.222408000	-0.290692000	2.829916000
6	1.374256000	-2.883168000	-1.281248000
1	1.993470000	-2.224196000	-1.898460000
6	-1.885429000	-2.457027000	0.340391000
1	-2.454811000	-3.112599000	1.023563000
1	-2.483369000	-2.286877000	-0.568098000
6	-2.524789000	3.791709000	1.600327000
1	-2.374946000	4.871744000	1.660447000
6	-0.252405000	-4.423585000	0.296706000
1	-0.922622000	-5.005715000	0.933819000
6	-0.586849000	-3.117599000	-0.072229000
6	4.575962000	0.777391000	1.512385000
1	5.428024000	0.696904000	2.193176000
6	2.580899000	2.313058000	-2.694967000
1	2.605823000	2.842975000	-3.650162000
6	0.954174000	-4.965304000	-0.162177000
1	1.242610000	-5.982654000	0.114220000
6	-3.553235000	3.159622000	2.303342000
1	-4.227895000	3.736905000	2.940798000
6	-3.710277000	1.775106000	2.182942000
1	-4.507276000	1.251516000	2.716782000
6	3.742053000	2.225381000	-1.888622000
1	4.687405000	2.669344000	-2.208794000
6	1.784918000	-4.182857000	-0.963783000
1	2.738173000	-4.563142000	-1.337259000
6	-2.111061000	0.917607000	-2.627448000
8	-1.737736000	2.081359000	-2.572390000
8	-1.751612000	-0.031818000	-1.802969000
6	-3.063775000	0.436684000	-3.718092000
1	-3.479122000	1.293983000	-4.264269000
1	-2.511308000	-0.207256000	-4.422454000
1	-3.875461000	-0.173853000	-3.292742000

Ir^{III}-OAc

77	-0.398078000	0.172471000	-0.152008000
7	0.186438000	2.156188000	-0.361664000
8	1.005894000	0.402615000	3.689955000
7	-1.724330000	1.001949000	1.238385000
7	0.855855000	-0.029997000	1.416393000
7	1.247697000	-0.693219000	-1.131699000
7	-1.390662000	-1.574551000	0.346287000
6	-1.978736000	2.392355000	0.751977000
1	-2.686132000	2.312406000	-0.086942000
1	-2.425489000	3.020129000	1.539715000
6	2.302613000	-0.894124000	-0.254492000
6	0.413822000	0.408625000	2.624745000
6	-0.702812000	3.009656000	0.214302000
6	4.591279000	-1.624534000	0.235153000
1	5.544143000	-2.037005000	-0.103994000
6	1.308385000	2.638763000	-0.922825000
1	1.984746000	1.904686000	-1.362789000
6	2.113278000	-0.550988000	1.125110000
6	3.544445000	-1.435327000	-0.705451000
6	-1.027468000	0.977101000	2.587074000
1	-0.982997000	1.998547000	2.992916000
1	-1.628381000	0.386897000	3.294548000
6	1.379039000	-1.025842000	-2.414003000
1	0.487319000	-0.906732000	-3.032499000
6	3.164305000	-0.757866000	2.018986000
1	3.020369000	-0.501573000	3.067275000
6	-1.021460000	-2.838162000	0.078629000
1	-0.111105000	-2.962474000	-0.507421000
6	-2.929794000	0.119394000	1.207461000
1	-3.562739000	0.270734000	2.096615000
1	-3.507752000	0.399094000	0.313848000
6	1.594801000	4.001930000	-0.945953000
1	2.514942000	4.351912000	-1.417136000
6	-3.305682000	-2.384853000	1.536613000
1	-4.208028000	-2.171597000	2.113355000
6	-2.525660000	-1.334742000	1.056919000
6	4.390775000	-1.290099000	1.561456000
1	5.196603000	-1.439274000	2.284542000
6	2.581761000	-1.559648000	-2.927311000
1	2.633918000	-1.811979000	-3.987858000
6	-2.924477000	-3.701538000	1.261702000
1	-3.526637000	-4.537113000	1.626023000
6	0.694386000	4.889314000	-0.355487000
1	0.892854000	5.963616000	-0.349839000
6	-0.465952000	4.382958000	0.236812000
1	-1.188455000	5.049529000	0.712198000
6	3.655037000	-1.759047000	-2.084956000
1	4.594051000	-2.172754000	-2.462778000
6	-1.767279000	-3.929857000	0.516995000
1	-1.435670000	-4.940780000	0.274153000
6	-2.130245000	-0.188377000	-2.634369000
8	-1.564535000	-1.250582000	-2.863006000
8	-1.854156000	0.589961000	-1.616001000
6	-3.224718000	0.355578000	-3.537733000
1	-3.455472000	-0.368732000	-4.328845000
1	-4.134064000	0.576055000	-2.956855000
1	-2.894584000	1.304153000	-3.990647000

$_{HEP}Ir^{TS}$

77	-0.167113000	-0.274646000	0.183290000
7	0.939380000	-1.963043000	0.765750000
8	1.795490000	-0.714537000	-3.351281000
7	-1.014136000	-1.679933000	-1.132546000
7	1.146692000	0.028841000	-1.264786000
7	0.954474000	1.194747000	1.126931000
7	-1.506406000	0.990626000	-0.770298000
6	-0.968998000	-2.973287000	-0.387115000
1	-1.773651000	-2.938007000	0.365215000
1	-1.157997000	-3.831072000	-1.053384000
6	2.024860000	1.567074000	0.336054000
6	1.048464000	-0.735967000	-2.392004000
6	0.368602000	-3.115012000	0.315361000
6	4.044283000	2.885922000	-0.071854000
1	4.771671000	3.630422000	0.259192000

6	2.106215000	-2.008075000	1.431423000
1	2.513931000	-1.051412000	1.762527000
6	2.151079000	0.955942000	-0.952649000
6	2.962509000	2.543404000	0.781853000
6	-0.147491000	-1.707545000	-2.374398000
1	0.256351000	-2.720051000	-2.523075000
1	-0.764453000	-1.484994000	-3.257087000
6	0.747101000	1.774350000	2.306211000
1	-0.173368000	1.487517000	2.823191000
6	3.222158000	1.323390000	-1.765336000
1	3.322831000	0.866537000	-2.748018000
6	-1.536537000	2.332446000	-0.717301000
1	-0.814403000	2.809780000	-0.056751000
6	-2.397425000	-1.186897000	-1.426574000
1	-2.807375000	-1.671984000	-2.326321000
1	-3.021229000	-1.403994000	-0.541019000
6	2.760338000	-3.210278000	1.692511000
1	3.703305000	-3.203826000	2.241994000
6	-3.334053000	1.008744000	-2.316391000
1	-4.039948000	0.451297000	-2.935154000
6	-2.398689000	0.319404000	-1.545817000
6	4.157867000	2.281417000	-1.309762000
1	4.988333000	2.548544000	-1.967833000
6	1.638565000	2.749201000	2.808167000
1	1.434440000	3.196827000	3.782363000
6	-3.363513000	2.404210000	-2.272220000
1	-4.092510000	2.959256000	-2.867350000
6	2.188755000	-4.399221000	1.237915000
1	2.678322000	-5.358521000	1.421415000
6	0.981067000	-4.347461000	0.535147000
1	0.512753000	-5.259378000	0.159083000
6	2.737600000	3.122234000	2.061506000
1	3.437371000	3.873783000	2.437017000
6	-2.452060000	3.075690000	-1.456264000
1	-2.444981000	4.164431000	-1.383698000
6	-3.140833000	0.436545000	1.949469000
8	-2.127885000	1.179663000	1.972398000
8	-3.195087000	-0.665354000	1.314004000
6	-4.372166000	0.836596000	2.756305000
1	-4.291389000	1.867467000	3.126196000
1	-5.279124000	0.719287000	2.143278000
1	-4.478301000	0.151799000	3.614023000
1	-0.807401000	-1.166636000	2.190803000
1	-1.533923000	-1.127855000	1.885894000

$_{RPP}Ir^{TS}$

77	-0.167113000	-0.274646000	0.183290000
7	0.939380000	-1.963043000	0.765750000
8	1.795490000	-0.714537000	-3.351281000
7	-1.014136000	-1.679933000	-1.132546000
7	1.146692000	0.028841000	-1.264786000
7	0.954474000	1.194747000	1.126931000
7	-1.506406000	0.990626000	-0.770298000
6	-0.968998000	-2.973287000	-0.387115000
1	-1.773651000	-2.938007000	0.365215000
1	-1.157997000	-3.831072000	-1.053384000
6	2.024860000	1.567074000	0.336054000
6	1.048464000	-0.735967000	-2.392004000
6	0.368602000	-3.115012000	0.315361000
6	4.044283000	2.885922000	-0.071854000
1	4.771671000	3.630422000	0.259192000
6	2.106215000	-2.008075000	1.431423000
1	2.513931000	-1.051412000	1.762527000
6	2.151079000	0.955942000	-0.952649000
6	2.962509000	2.543404000	0.781853000
6	-0.147491000	-1.707545000	-2.374398000
1	0.256351000	-2.720051000	-2.523075000
1	-0.764453000	-1.484994000	-3.257087000
6	0.747101000	1.774350000	2.306211000
1	-0.173368000	1.487517000	2.823191000
6	3.222158000	1.323390000	-1.765336000
1	3.322831000	0.866537000	-2.748018000
6	-1.536537000	2.332446000	-0.717301000
1	-0.814403000	2.809780000	-0.056751000
6	-2.397425000	-1.186897000	-1.426574000
1	-2.807375000	-1.671984000	-2.326321000
1	-3.021229000	-1.403994000	-0.541019000
6	2.760338000	-3.210278000	1.692511000
1	3.703305000	-3.203826000	2.241994000
6	-3.334053000	1.008744000	-2.316391000
1	-4.039948000	0.451297000	-2.935154000
6	-2.398689000	0.319404000	-1.545817000
6	4.157867000	2.281417000	-1.309762000
1	4.988333000	2.548544000	-1.967833000
6	1.638565000	2.749201000	2.808167000
1	1.434440000	3.196827000	3.782363000
6	-3.363513000	2.404210000	-2.272220000
1	-4.092510000	2.959256000	-2.867350000
6	2.188755000	-4.399221000	1.237915000
1	2.678322000	-5.358521000	1.421415000
6	0.981067000	-4.347461000	0.535147000
1	0.512753000	-5.259378000	0.159083000
6	2.737600000	3.122234000	2.061506000
1	3.437371000	3.873783000	2.437017000
6	-2.452060000	3.075690000	-1.456264000
1	-2.444981000	4.164431000	-1.383698000
6	-3.140833000	0.436545000	1.949469000
8	-2.127885000	1.179663000	1.972398000
8	-3.195087000	-0.665354000	1.314004000
6	-4.372166000	0.836596000	2.756305000
1	-4.291389000	1.867467000	3.126196000
1	-5.279124000	0.719287000	2.143278000
1	-4.478301000	0.151799000	3.614023000
1	-0.807401000	-1.166636000	2.190803000
1	-1.533923000	-1.127855000	1.885894000

$_{LCP}Ir^{TS1}$

77	-0.612457000	-0.534514000	-0.549395000
7	0.956069000	2.218430000	-1.668705000
8	0.804345000	1.781157000	2.775554000
7	-1.594051000	1.709522000	0.106088000
7	0.587291000	0.169180000	1.105347000
7	1.223113000	-1.716255000	-0.712323000
7	-2.690357000	-0.874556000	-0.080618000
6	-1.117014000	2.922644000	-0.605236000
1	-1.555309000	2.906991000	-1.607190000
1	-1.489309000	3.823469000	-0.094465000
6	2.108060000	-1.508228000	0.317377000
6	0.190888000	1.236147000	1.860661000
6	0.385428000	3.036129000	-0.762531000
6	4.207350000	-2.026405000	1.480397000
1	5.124013000	-2.604171000	1.551253000
6	2.267628000	2.278870000	-1.921968000
1	2.647665000	1.574752000	-2.656928000

6	1.794473000	-0.504145000	1.293866000
6	3.315772000	-2.266388000	0.404923000
6	-1.233493000	1.731019000	1.541071000
1	-1.342559000	2.736556000	1.967824000
1	-1.926989000	1.070999000	2.074106000
6	1.495778000	-2.618918000	-1.654398000
1	0.765295000	-2.719689000	-2.450955000
6	2.705576000	-0.304420000	2.332178000
1	2.489431000	0.448694000	3.076028000
6	-3.130697000	-2.141475000	0.093581000
1	-2.414786000	-2.924856000	-0.127113000
6	-3.042294000	1.549325000	-0.112562000
1	-3.627037000	2.281207000	0.465705000
1	-3.239864000	1.745780000	-1.173887000
6	3.093409000	3.198833000	-1.279871000
1	4.152442000	3.234665000	-1.511043000
6	-4.825286000	-0.074513000	0.655553000
1	-5.468808000	0.771183000	0.876490000
6	-3.531590000	0.152781000	0.189377000
6	3.892832000	-1.064195000	2.414872000
1	4.569348000	-0.875155000	3.243363000
6	2.659809000	-3.404790000	-1.632841000
1	2.829652000	-4.131939000	-2.419447000
6	-5.279822000	-1.381049000	0.826350000
1	-6.286628000	-1.571081000	1.184823000
6	2.524068000	4.056364000	-0.339799000
1	3.136812000	4.780282000	0.188274000
6	1.153554000	3.976211000	-0.079262000
1	0.688078000	4.632289000	0.648501000
6	3.562207000	-3.228823000	-0.603544000
1	4.471797000	-3.821808000	-0.557085000
6	-4.414626000	-2.434796000	0.534169000
1	-4.717563000	-3.469219000	0.653384000
1	-0.346697000	0.302285000	-2.175939000
1	0.198973000	1.091760000	-2.019386000
